# Prismarenes: A New Class of Macrocyclic Hosts Obtained by Templation in a Thermodynamically Controlled Synthesis

**DOI:** 10.1021/jacs.9b12216

**Published:** 2020-01-03

**Authors:** Paolo Della Sala, Rocco Del Regno, Carmen Talotta, Amedeo Capobianco, Neal Hickey, Silvano Geremia, Margherita De Rosa, Aldo Spinella, Annunziata Soriente, Placido Neri, Carmine Gaeta

**Affiliations:** † Laboratory of Supramolecular Chemistry, Dipartimento di Chimica e Biologia, “A. Zambelli”, Università di Salerno, Via Giovanni Paolo II 132, I-84084 Fisciano (SA), Italy; ‡ Centro di Eccellenza in Biocristallografia, Dipartimento di Scienze Chimiche e Farmaceutiche, Università di Trieste, Via L. Giorgieri 1, I-34127 Trieste, Italy

## Abstract

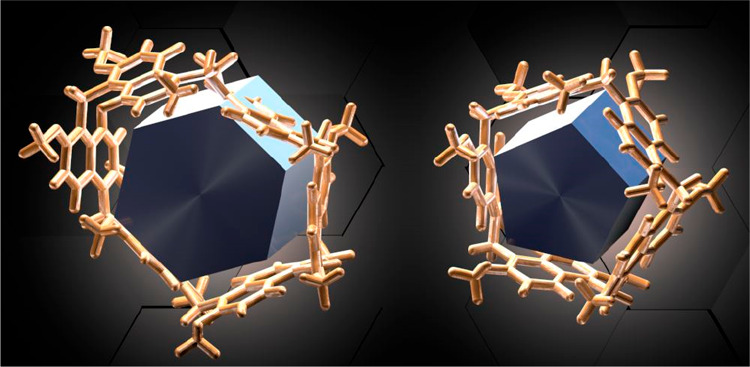

The novel title macrocycles, based on methylene-bridged 1,5-naphthalene units, have been obtained by template effect in a thermodynamically controlled synthesis. In detail, the prism[5]arene **1** or the prism[6]arene **3** was selectively removed from the equilibrium mixture by using the complementary ammonium-templating agent. When only the solvent 1,2-DCE was used, the 1,4-confused derivative **2** was obtained. The prism[5]arene here described shows a deep π-electron-rich aromatic cavity that exhibits a great affinity for the quaternary ammonium guests, originating from favorable cation···π and ^+^NC–H···π interactions. This recognition motif is the basis of the templated synthesis of the prism[*n*]arenes here reported.

Since 1967, when Charles Pedersen^1^ reported the first template synthesis of crown-ethers, a plethora of peculiar macrocyclic structures have been designed and obtained by guest-templated^2^ strategies. Even now, supramolecular chemists have never stopped imagining novel and intriguing macrocyclic structures. Among them, pillararenes^3^ and oxatubarenes^4^ have recently shown intriguing supramolecular functions and properties.^5^ Inspired by the attractive shapes of oxatubarenes and pillararenes and encouraged by their supramolecular performances,^5^ we have envisioned novel cyclo-structures (e.g., **1** and **3** in Scheme 1), based on methylene-bridged 1,5-naphthalene units.^6^ We were attracted by their deep π-electron-rich aromatic cavity and by their prism shape (*vide infra*), which has inspired the name *prismarene*.^7^ The prismarenes **1**–**3**, as well as pillararenes, can be classified as cyclophanes.^8^ The cyclophanes^8^ are generally obtained by a reversible acid- or base-catalyzed condensation of the respective monomers with an aldehyde. Interestingly, in some cases, under thermodynamically controlled macrocyclization conditions, the selectivity toward a specific cyclo-oligomer can be driven by a template effect.^9−12^ In this way, it can be possible to isolate a specific macrocycle from an equilibrium mixture by adding an appropriate complementary guest.^10−12^ In this regard, a well-known example of thermodynamically controlled synthesis concerns the pillararene macrocycles.^9,11,12^

**Scheme 1:**
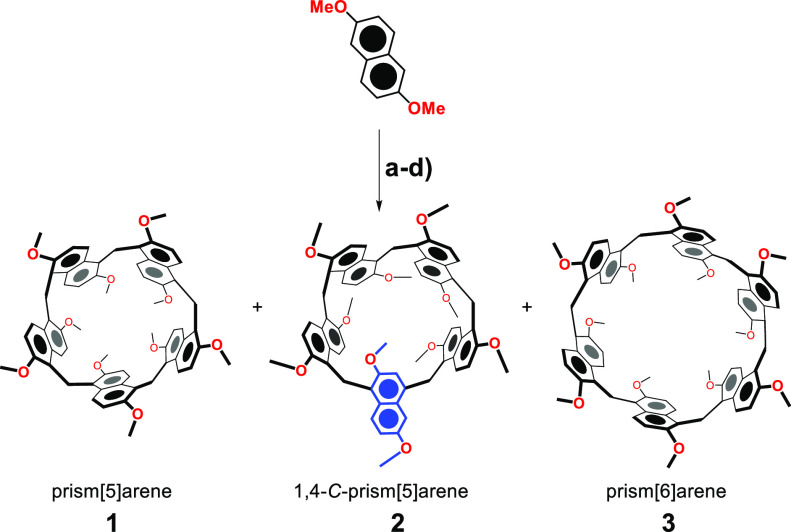
Synthesis of Prism[*n*]arenes **1**–**3** Reagents and conditions: (a) 1,2-DCE, TFA, paraformaldehyde, 70 °C, 22 h: **1** (0.3%), **2** (40%); (b) conditions *a* and **4**^2+^·2I^–^: **1** (47%), **2** (16%); (c) conditions *a* and **5**^+^·I^–^: **1** (32%), **2** (8%); (d) **6**^+^·I^–^, 1,2-DCE, TFA, 70 °C, 72 h: **1** (0.3%), **2** (6%), **3** (20%).

In analogy with other examples of cyclophanes,^3,13^ we started the synthetic approaches to prismarenes by using the monomer 2,6-dimethoxynaphthalene and formaldehyde in the presence of an acid catalyst. In initial tests, when 2,6-dimethoxynaphthalene (0.5 M)^13^ was reacted in 1,2-dichloroethane (1,2-DCE) as the solvent, with paraformaldehyde in the presence of BF_3_·Et_2_O at 30 °C, only linear oligomers were obtained.^14^ With the aim of obtaining cyclic structures, we resorted to different conditions, which included the use of trifluoroacetic acid (TFA) as the acid, higher reaction temperature, and dilution. Thus, when 2,6-dimethoxynaphthalene (2.5 mM) and paraformaldehyde (1.2 equiv) were reacted in the presence of TFA (15 equiv) in DCE at 70 °C, the envisioned prism[5]arene macrocycle **1** was obtained in 0.3% yield after 22 h (Scheme 1), while its 1,4-confused^15^ isomer (1,4-*C*-prism[5]arene) **2** was obtained in higher yield (40%).

The possible role of the solvent for the formation of prism[5]arene **1** and 1,4-*C*-prism[5]arene **2** was then explored. Thus, **2** was obtained in lower yield when solvents such as *o*-dichlorobenzene or chloroform were used (Table S1). Temperature was also found to be crucial for the formation of **2**. Indeed, only 11% of 1,4-*C*-prism[5]arene was collected when the reaction was performed at room temperature. The HR-ESI mass spectrum confirmed the molecular mass of **2** (found: 1000.4218 *m*/*z*, calculated for [M]^+^ 1000.4186). Detailed 1D and 2D NMR studies (SI) at 213 K clearly indicated that 4/5 of the naphthalene rings of **2** were bridged through their 1,5-positions, while the naphthalene group in blue in Scheme 1 showed a 1,4-bridging pattern (*confused*-naphthalene ring), as confirmed by X-ray crystallographic analysis (Figure 1). Interestingly, when the TFPB^–^ salt^16^ of dication **4**^**2+**^ (TFPB: tetrakis[3,5-bis(trifluoromethyl)phenyl]borate) was added to a CD_2_Cl_2_ solution of **2**, its ^1^H NMR spectrum at 183 K (600 MHz, SI) showed dramatic changes indicative of the formation of a pseudo[2]rotaxane.^16^ In detail, the formation of the **4**^**2+**^ ⊂ **2** pseudorotaxane was ascertained by the presence of shielded ^1^H NMR signals at negative value of chemical shifts (from 0 to −2 ppm, SI) attributable to the protons of the guest inside the aromatic cavity of the host.^16^ An association constant value of 470 M^–1^ was calculated by direct integration of the slowly exchanging ^1^H NMR signals^17^ of the threaded **4**^**2+**^ ⊂ **2** and the free species (SI, Figure 2).

**Figure 1:**
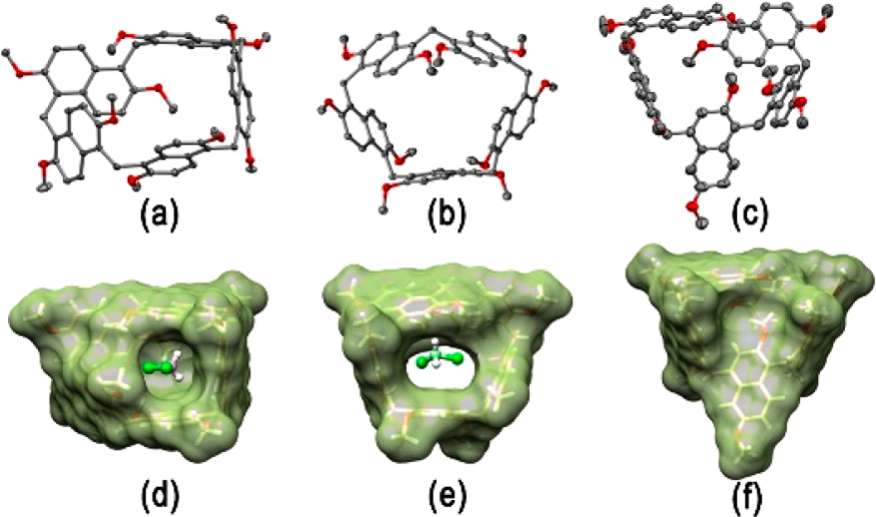
Ellipsoid representation (50% probability) of the (a) α and (b) γ forms of prism[5]arene **1** and  (c) β form of 1,4-*C*-prism[5]arene **2**. Solvent molecules, disordered atoms with low occupancy factors, and hydrogen atoms are not included for clarity. For each molecule the α and β forms have similar conformations. The solvent-excluded molecular surfaces (1.4 Å probe) of the (d) α and (e) γ forms of prism[5]arene and (f) β form of 1,4-*C*-prism[5]arene. The encapsulated CH_2_Cl_2_ solvent molecules are represented as a ball-and-stick model.

**Figure 2:**
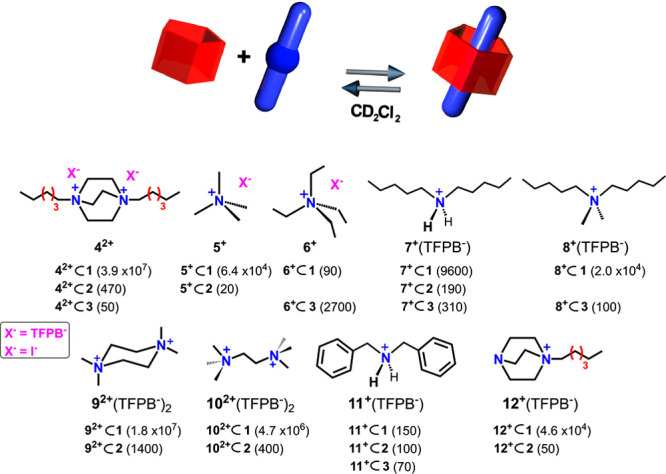
(Top) Schematic complexation equilibrium of the prism[5]arene **1** with guests **4**^2+^–**12**^**+**^. Binding constant values of their host–guest complexes with the prism[*n*]arenes **1**–**3**, determined by ^1^H NMR experiments in CD_2_Cl_2_ (600 MHz) (SI). Errors <15% calculated as mean values of three measures.

With this result in hand, we decided to perform the synthesis of prismarenes in the presence of **4**^**2+**^ as iodide salt (Figure 2), with the aim of investigating its possible template effect over the thermodynamic equilibrium^12^ distribution of the cyclo-oligomers in Scheme 1. When 2,6-dimethoxynaphthalene (2.5 mM) and paraformaldehyde (1.2 equiv) were reacted in the presence of TFA (15 equiv) in 1,2-DCE at 70 °C, and by adding the **4**^**2+**^ iodide salt, impressively, prism[5]arene **1** was obtained in 47% yield after 22 h. Interestingly, the yield of **1** was decreased to 32% when the tetramethylammonium cation **5**^**+**^ was instead used as potential templating agent. The HR-ESI mass spectrum of prism[5]arene **1** confirmed its molecular mass. 1D and 2D NMR spectra (CD_2_Cl_2_, 298 K, 600 MHz) were in accord with the *D*_5_ symmetry of **1**.

Small colorless single crystals of three pseudopolymorphic forms of prism[5]arene **1** and two pseudopolymorphic forms of 1,4-*C*-prism[5]arene **2**, suitable for X-ray structure determination, were analyzed using synchrotron radiation and cryo-cooling techniques. The three prism[5]arene pseudopolymorphs, α, β, and γ forms (Figure 1 and SI), are distinguished by a different amount of cocrystallized CH_2_Cl_2_ solvent molecules (three, two, or one solvent molecule for each prism[5]arene molecule, respectively). All three forms are composed of a racemic mixture of inherently chiral prism[5]arene molecules, in which all the naphthalene moieties show the same orientation of the 2,6-methoxy substitution pattern (Figure 1a,b). In the α and β forms, there is formation of a molecular cavity sealed on one site by a methoxy group (Figure 1d), while in the more symmetric γ form a central hole is present (Figure 1e) (see SI). In all three pseudopolymorphs, the CH_2_Cl_2_ molecules hosted inside each prism[5]arene hole/cavity are sealed by the neighboring molecules (void volume of 85–95 Å^3^, 67–60% filled by the vdW volume of a CH_2_Cl_2_ molecule). The two pseudopolymorphic forms of 1,4-*C*-prism[5]arene **2** (α = monoclinic form, β = triclinic form) are also composed of a racemic mixture of inherently chiral molecules. The 1,4-naphthalene ring assumes a conformation that completely fills the cavity of the prismarene (Figure 1f).

At this point of our study, we decided to perform a series of experiments in order to investigate a possible interconversion process between the two isomers, the prism[5]arene **1** and the 1,4-*C*-prism[5]arene **2**. When prism[5]arene **1** was heated at 70 °C in 1,2-DCE and in the presence of TFA, the conversion to 1,4-*C*-prism[5]arene **2** was complete after 16 h. This result clearly indicates that 1,4-*C*-prism[5]arene **2** is the thermodynamic isomer, while prism[5]arene **1** is the kinetic one. Density functional theory (DFT) calculations (SI) substantially agree with these results. The prism[5]arene **1** is predicted to be less stable than its confused-isomer **2** by 5.1 kcal/mol. Interestingly, this energy difference decreases to 2.6 kcal/mol when considering the equilibrium geometries of **1** and **2** hosting 1,2-DCE inside their cavity. We have also computed the equilibrium geometries of the most stable transition states (TS) for the macrocyclization steps of the intermediate carbocation. The TS (SI) for the 1,4 attack is predicted to lie 3.3 kcal/mol above the one involved in 1,5 attack. This energy difference increases to 4.3 kcal/mol when considering 1,2-DCE inside the cavity, thus confirming that formation of the 1,5 adduct occurs faster.

To add further support to our points, we examined the reaction in Scheme 1 by HPLC (SI). In accordance with the above conclusion, after 270 min of reaction, prism[5]arene **1** is clearly the favored product. In fact, the amount of **1** is larger than that of its *C*-confused-isomer **2**. After 330 min the 1,4-*C*-isomer **2** prevails over **1** (SI). Differently, when the reaction was conducted in the presence of guest **4**^**2+**^, the HPLC monitoring evidenced that prism[5]arene **1** was the favored product over time.

Finally, when 1,4-*C*-prism[5]arene **2** was treated in the presence of **4**^**2+**^ in 1,2-DCE at 70 °C for 22 h, derivative **1** was obtained in 20% yield. In summary, these results clearly indicate that the formation of **1** and **2** occurs through a thermodynamically controlled templated process (Figure 3) in which the solvent 1,2-DCE and the cationic guest **4**^**2+**^ act as templating agent in the formation of 1,4-*C*-prism[5]arene **2** and prism[5]arene **1**, respectively.^12^ It is worth mentioning here that this result is also an uncommon example of template control over the regiochemistry of a macrocyclic product.^12^

**Figure 3:**
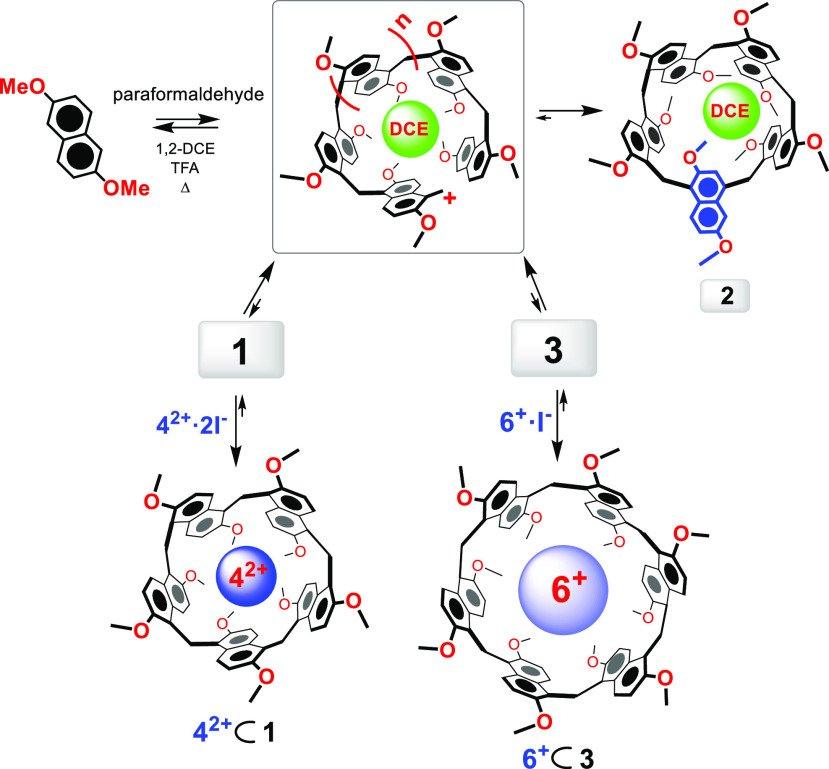
Thermodynamically controlled templated synthesis of prism[*n*]arenes.

With this results in hand, we investigated the use of a different templating agent. Thus, when the tetraethylammonium cation **6**^**+**^ was used as iodide salt (Scheme 1 and Figure 3), the prism[6]arene **3** was isolated in 20% yield, in addition to 1,4-*C*-prism[5]arene **2** (6%) and prism[5]arene **1** (0.3%). This result clearly indicates that the equilibrium distribution of the prism[*n*]arenes **1**–**3** can be controlled by using the appropriate complementary templating agent able to remove its host from the equilibrium mixture (Figure 3). Significantly, prism[6]arene **3** was quantitatively converted to 1,4-*C*-prism[5]arene **2** after treatment with TFA at 70 °C, for 16 h in 1,2-DCE, thus confirming that **2** is the thermodynamic product, while **1** and **3** are the kinetic ones in the equilibrium mixture.

Our attention then turned to the recognition ability of prism[*n*]arenes **1**–**3** (Figures 2 and 4 and SI). In detail, when 1,4-dihexyl-DABCO **4**^**2+**^, as TFPB^–^ salt, was added to a CD_2_Cl_2_ solution of prism[5]arene **1** (in equimolar ratio), the formation of pseudo[2]rotaxane **4**^**2+**^ ⊂ **1** (Figure 4e,f) was observed, as confirmed by 1D and 2D NMR studies and HR-MS-CID spectrum (SI). An association constant value of 3.9 × 10^7^ M^–1^ (298 K, CD_2_Cl_2_) was determined for the **4**^**2+**^ ⊂ **1** complex by a series of competition experiments^18^ (Figure 2 and SI). This value is significantly higher than that observed for the formation of **4**^**2+**^ ⊂ **2** pseudorotaxane (470 M^–1^). This result is in accord with the findings summarized in Figure 3.

**Figure 4:**
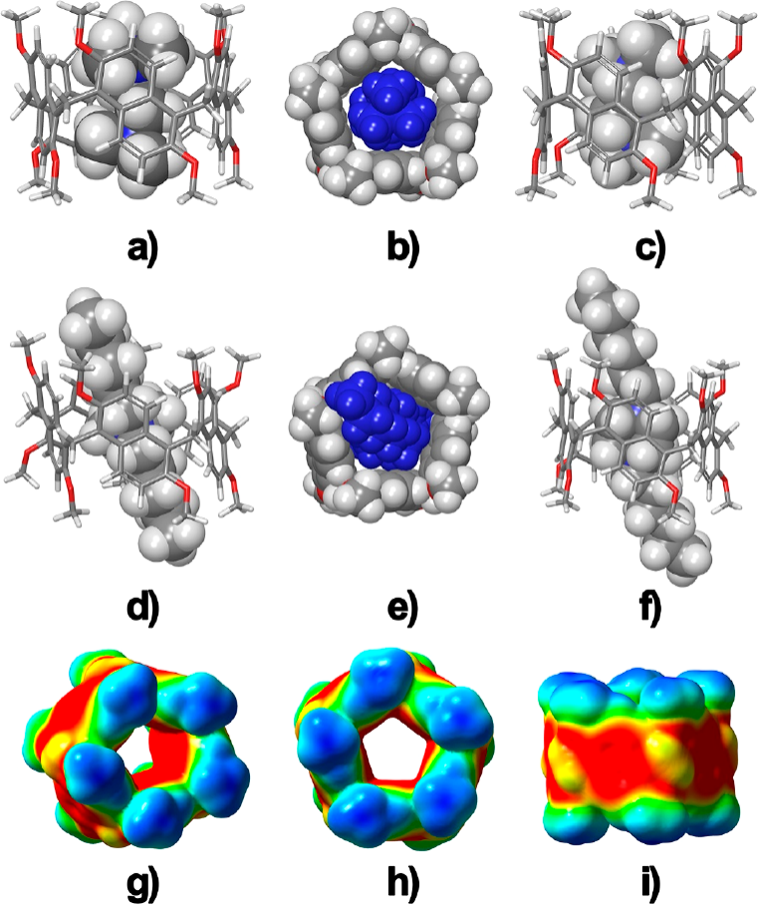
DFT-optimized structures (B97D3/SVP/SVPFIT) of complexes (a, b) **10**^2+^ ⊂ **1**; (c) **9**^2+^ ⊂ **1**; (d) **8**^+^ ⊂ **1**; (e, f) **4**^2+^ ⊂ **1**. (g–i) ESPs mapped onto electron density isosurfaces (ρ = 0.004) for the prism[5]arene **1**.

In fact, the guest **4**^**2+**^ shows a greater thermodynamic affinity for prism[5]arene **1** with respect to its *C*-isomer **2**, and therefore it plays the templating agent role in its synthesis. Analogous conclusions are inferred by comparing the association constant values of the two *endo*-complexes of the tetramethylammonium **5**^**+**^ cation (as TFPB^–^ salt), **5**^**+**^ ⊂ **1** (6.4 × 10^4^ M^–1^) and **5**^**+**^ ⊂ **2** (20 ± 5 M^–1^). A close inspection of the DFT-optimized structure of the pseudo[2]rotaxane **4**^**2+**^ ⊂ **1** (Figure 4) evidenced the crucial role played by C–H···π and cation···π interactions in the stabilization of the complex. The prism[5]arene macrocycle presents an extended π-electron-rich aromatic cavity (Figures 4g–i) in which these interactions are enhanced. In fact, when the *N,N*-dimethyl-*N,N*-dipentylammonium **8**^**+**^·TFPB^–^ salt was mixed with **1** in CD_2_Cl_2_, the pseudo[2]rotaxane **8**^**+**^ ⊂ **1** was formed (Figure 4d) with an association constant of 2.0 × 10^4^ M^–1^, a value significantly higher than that found for the analogous pseudo[2]rotaxane **7**^**+**^ ⊂ **1** (9600 M^–1^) obtained with the secondary dipentylammonium **7**^**+**^ axle. This result confirms the crucial role played by the ^+^NC–H···π interactions for the stabilization of the complex between ammonium axles and the prism[5]arene. Finally, the prism[5]arene **1** forms ammonium-based complexes thermodynamically more stable than its *C*-confused isomer **2**.

In conclusion, a novel class of macrocyclic hosts, named prismarenes, have been obtained by a templated approach of a thermodynamically controlled synthesis. The prism[*n*]arenes here described show a good affinity for ammonium guests and form pseudorotaxane architectures stabilized by cation···π and ^+^NC–H···π interactions. Considering the current enormous interest directed to the synthesis of novel macrocyclic hosts, which have already found interesting nanotechnological and supramolecular applications,^5^ and considering the peculiar features of the prismarenes, it is conceivable that the above results will pave the way to a quick expansion of this new area of research in supramolecular chemistry.

## Supporting Information Available

Detailed synthetic procedures, 1D and 2D NMR spectra of prismarenes and their complexes, HR mass spectra, details on stability constant determination, DFT calculation details. X-ray figures, and tables of crystal data (PDF)
X-ray crystallographic data for α form of prism[5]arene **1** (CIF)
X-ray crystallographic data for β form of prism[5]arene **1** (CIF)
X-ray crystallographic data for γ form of prism[5]arene **1** (CIF)
X-ray crystallographic data for α form of 1,4-confused-prism[5]arene **2** (CIF)
X-ray crystallographic data for β form of 1,4-confused-prism[5]arene **2** (CIF)

## Supplementary Material

ja9b12216_si_001.pdf

ja9b12216_si_002.cif

ja9b12216_si_003.cif

ja9b12216_si_004.cif

ja9b12216_si_005.cif

ja9b12216_si_006.cif
