# Supplementary material for: Prismarenes: A New Class of Macrocyclic Hosts Obtained by Templation in a Thermodynamically Controlled Synthesis
Source: J Am Chem Soc. 2020 Jan 3;142(4):1752–6. doi: 10.1021/jacs.9b12216 (PMC7993634; doi:10.1021/jacs.9b12216)
Supplement: Supplementary file 1 — ja9b12216_si_001.pdf [file ja9b12216_si_001.pdf]

Supplementary Information

Prismarenes: A New Class of Macrocyclic Hosts Obtained by Templation in a Thermodynamically Controlled Synthesis

Paolo Della Sala,[a] Rocco Del Regno,[a] Carmen Talotta,[a] Amedeo Capobianco,[a] Neal Hickey,[b] Silvano Geremia,[b] Margherita De Rosa,[a] Aldo Spinella,[a] Annunziata Soriente,[a] Placido Neri,[a] and Carmine Gaeta*,[a]

†Laboratory of Supramolecular Chemistry, Dipartimento di Chimica e Biologia, "A. Zambelli", Università di Salerno, Via Giovanni Paolo II 132, Fisciano (SA), I-84084, Italy. ‡Centro di Eccellenza in Biocristallografia, Dipartimento di Scienze Chimiche e Farmaceutiche, Università di Trieste, Via L. Giorgieri 1, I-34127 Trieste, Italy.

**Table of Contents**	**Pages**
General Experimental Details	**S2**
General procedure for the synthesis of the Prism[n]arenes	**S2**
Copies of NMR and HR Mass Spectra of Prism[n]arenes	**S5**
Copies of NMR and HR Mass Spectra of Prismarene Complexes	**S24**
^{1}H NMR determination of K_{ass} values	**S58**
HPLC analysis of the cyclocondensation kinetics	**S74**
Determination of the crystallographic structures of Prism[5]arene (1) and 1,4-confused-Prism[5]arene (2)	**S75**
Equilibrium geometries and electronic energies	**S82**
References	**S96**

General Experimental Details

HR MALDI mass spectra were recorded on a Bruker Solarix FT-ICR mass spectrometer equipped with a 7T magnet. The samples recorded in MALDI were prepared by mixing 10 μL of analyte in dichloromethane (1 mg/mL) with 10 μL of solution of 2,5-dihydroxybenzoic acid (10 mg/mL in Metanol). The mass spectra were calibrated externally, and a linear calibration was applied. All reaction solvents were dried by activated 3 Å molecular sieves[1]. All chemicals reagents grade was used without further purification and were used as purchased. Reaction temperatures were measured externally. Reactions were monitored by TLC silica gel plates (0.25 mm) and visualized by UV light 254 nm, or by spraying with H_2SO_4–$Ce(SO_4)_2$. NMR spectra were recorded on a Bruker Avance-600 [600 (^{1}H) and 150 MHz (^{13}C)], Avance-400 [400 (^{1}H) and 100 MHz (^{13}C)] or Avance-300 MHz [300 (^{1}H) and 75 MHz (^{13}C)] spectrometers. Chemical shifts are reported relative to the residual solvent peak[2]. Standard pulse programs, provided by the manufacturer, were used for 2D COSY-45, 2D HSQC, 2D HMBC and 2D NOESY experiments.

General procedure for the synthesis of Prism[n]arenes.

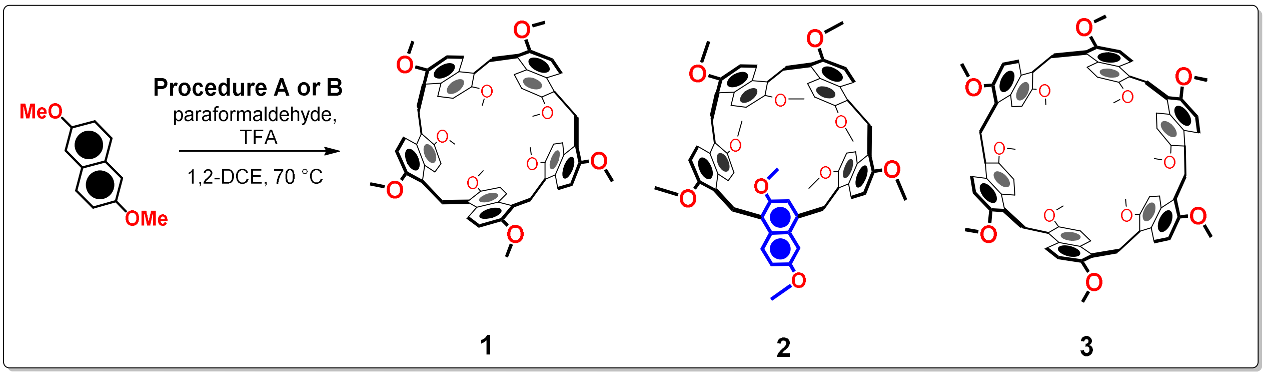

Procedure A. A solution of 2,6-dimethoxynaphthalene (250 mg, 1.33 mmol), paraformaldehyde (48 mg, 1.60 mmol, 1.2 equiv) in 530 mL of dry 1,2-dichloroethane was heated to 70°C, then trifluoroacetic acid (1.5 mL, 0.02 mol, 15 equiv) was added. The solution was stirred for 22 h at 70°C and subsequently the solvent evaporated under reduced pressure. The residue was dissolved in CH_2Cl_2 (30 mL) and the mixture was washed with an aqueous saturated solution of $NaHCO_3$ (30 mL). Finally, the organic layer was washed with brine (2x20 mL), and the organic phases were dried over sodium sulfate and concentrated to give a solid light brown. The crude product was purified by chromatographic column on silica gel (hexane/dichloromethane = 1/9). Macrocycle **1** was obtained in 0.3 % of yield (1 mg) and macrocycle **2** was obtained as a white solid (106 mg, 40 %).

Procedure B. A solution of 2,6-dimethoxynaphthalene (250 mg, 1.33 mmol), paraformaldehyde (48 mg, 1.60 mmol, 1.2 equiv), and templating agent, $\mathbf{4^{2+}{\cdot}2I^-}$, $\mathbf{5^{+}{\cdot}I^-}$ or $\mathbf{6^{+}{\cdot}I^-}$ (1.33 mmol, 1.0 equiv) in 530 mL of dry 1,2-dichloroetane was heated to 70° C, then trifluoroacetic acid (1.5 mL, 0.02 mol, 15 equiv) was added. The solution was stirred for 22 h (in presence of $\mathbf{4^{2+}{\cdot}2I^-}$ and $\mathbf{5^{+}{\cdot}I^-}$) or 72 h (in presence of $\mathbf{6^{+}{\cdot}I^-}$) at 70°C and subsequently the solvent evaporated under reduced pressure. The residue was dissolved in CH_2Cl_2 (30 mL) and the mixture was washed with an aqueous saturated solution of $NaHCO_3$ (30 mL). Finally, the organic layer was washed with brine (2x20 mL), and the organic phases were dried over sodium sulfate and concentrated to give a solid light brown. The crude product was purified by chromatographic column on silica gel (hexane/dichloromethane = 1/9).

Template synthesis in presence of $4^{2+}\cdot 2I^-$ salt: The macrocycle **1** was obtained in 47 % yield (125 mg) and the macrocycle **2** in 16 % yield (42 mg).

Template synthesis in presence of $5^{+}\cdot I^-$ salt: The macrocycle **1** was obtained in 32% of yield (85 mg) and the macrocycle **2** in 8% of yield (21 mg).

Template synthesis in presence of $6^{+}\cdot I^-$ salt: The macrocycle **1** was obtained in 0.3 % yield (1 mg), the macrocycle **2** in 6 % yield (16 mg) and the macrocycle **3** was obtained in 20 % yield (54 mg).

Derivative 1:

Mp: >374 °C dec.

^{1}H NMR (CD_2Cl_2, 600 MHz, 298 K): *δ* 7.95 (*d*, 10H, Ar-*H, J* = 9.4 Hz), 6.92 (*d*, 10H, Ar-*H, J* = 9.4 Hz), 4.68 (*s*, 10H, ArC*H*$_2$Ar), 3.63 (*s*, 30H, O*Me*).
^{13}C NMR (CD_2Cl_2, 150 MHz, 298 K): *δ* 153.1, 129.8, 125.0, 123.7, 113.9, 56.8, 21.8.
DEPT-135 (CD_2Cl_2, 150 MHz, 298 K): *δ* 125.0, 113.9, 56.8, 21.8.
HRMS (MALDI) m/z $[M]^+$ calcd for $C_{65}H_{60}O_{10}$: 1000.4186. found: 1000.4227.

Derivative 2:

Mp: >385 °C dec.

^{1}H NMR (CD_2Cl_2, 600 MHz, 298 K): *δ* 8.37-8.35 (*overlapped*, 2H, Ar-*H*), 8.19 (d, 1H, Ar-*H*, *J* = 9.4 Hz), 7.63 (d, 1H, Ar-*H*, *J* = 9.3 Hz), 7.58 (d, 1H, Ar-*H*, *J* = 9.4 Hz), 7.53 (d, 1H, Ar-*H*, *J* = 2.6 Hz), 7.46 (d, 1H, Ar-*H*, *J* = 9.4 Hz), 7.43 (d, 1H, Ar-*H*, *J* = 9.4 Hz), 7.34 (d, 1H, Ar-*H*, *J* = 9.4 Hz), 7.30-7.27 (*overlapped*, 2H, Ar-*H*), 7.26 (s, 1H, Ar-*H*), 7.18 (d, 1H, Ar-*H*, *J* = 9.4 Hz), 7.12 (d, 1H, Ar-*H*, *J* = 9.4 Hz), 6.97 (d, 1H, Ar-*H*, *J* = 9.4 Hz), 6.82 (d, 1H, Ar-*H*, *J* = 9.4 Hz), 6.78 (d, 1H, Ar-*H*, *J* = 9.4 Hz), 6.45 (d, 1H, Ar-*H*, *J* = 9.4 Hz), 6.29 (d, 1H, Ar-*H*, *J* = 9.4 Hz), 4.98-4.83 (*overlapped*, 6H, ArC*H*$_2$Ar and Ar-*H*), 4.70-4.64 (*overlapped*, 2H, ArC*H*$_2$Ar), 4.51-4.36 (*overlapped*, 3H, ArC*H*$_2$Ar), 4.21 (s, 3H, O*Me*), 4.20 (s, 3H, O*Me*), 4.04 (s, 3H, O*Me*), 4.01 (s, 3H, O*Me*), 4.00(s, 3H, O*Me*), 3.69 (s, 3H, O*Me*), 3.67 (s, 3H, O*Me*), 2.84 (s, 3H, O*Me*), 2.17 (s, 3H, O*Me*), -0.03 (s, 3H, O*Me*).
^{13}C NMR (CD_2Cl_2, 150 MHz, 213 K): *δ* 155.6, 153.1, 152.7, 152.0, 151.6, 151.5, 151.4, 151.1, 150.9, 150.5, 134.4, 129.2, 128.8, 128.7, 128.6, 128.2, 128.0, 127.9, 127.5, 125.1, 124.9, 124.7, 124.3, 124.0, 123.9, 123.7, 123.6, 123.4, 123.0, 122.8, 122.4, 120.9, 119.8, 117.9, 117.2, 116.9, 115.4, 112.5, 112.2, 111.7, 111.6, 110.9, 110.7, 102.1, 56.7, 56.5, 56.2, 56.1, 56.0, 55.8, 55.6, 55.2, 54.7, 53.3, 26.6, 23.4, 21.6, 20.3, 20.1.
HRMS (MALDI) m/z $[M]^+$ calcd for $C_{65}H_{60}O_{10}$: 1000.4186. found: 1000.4218.

Derivative 3:

Mp: >352 °C dec.

^{1}H NMR (TCDE, 300 MHz, 393 K): *δ* 7.64 (*d*, 12H, Ar-*H, J* = 9.3 Hz), 6.72 (*d*, 12H, Ar-*H, J* = 9.3 Hz), 4.61 (*s*, 12H, ArC*H*$_2$Ar), 3.27 (*s*, 36H, O*Me*).
^{13}C NMR (TCDE, 75 MHz, 298 K): *δ* 152.1, 129.2, 123.9, 123.7, 114.1, 56.5, 29.6.
HRMS (MALDI) m/z $[M]^+$ calcd for $C_{78}H_{72}O_{12}$: 1200.5024. found: 1200.5117.

Table S1: Procedure A. Yields of **1** and **2** in the presence different solvents.

Solvent	Yield of **1**	Yield of **2**
o-dichlorobenzene	----	9.4 %
Chloroform	----	9.6 %
1,1,2, -trichloroethane	----	----
1,1,2,2-tetrachloroethane	----	18 %

Copies of NMR and HR Mass Spectra of Prism[n]arenes

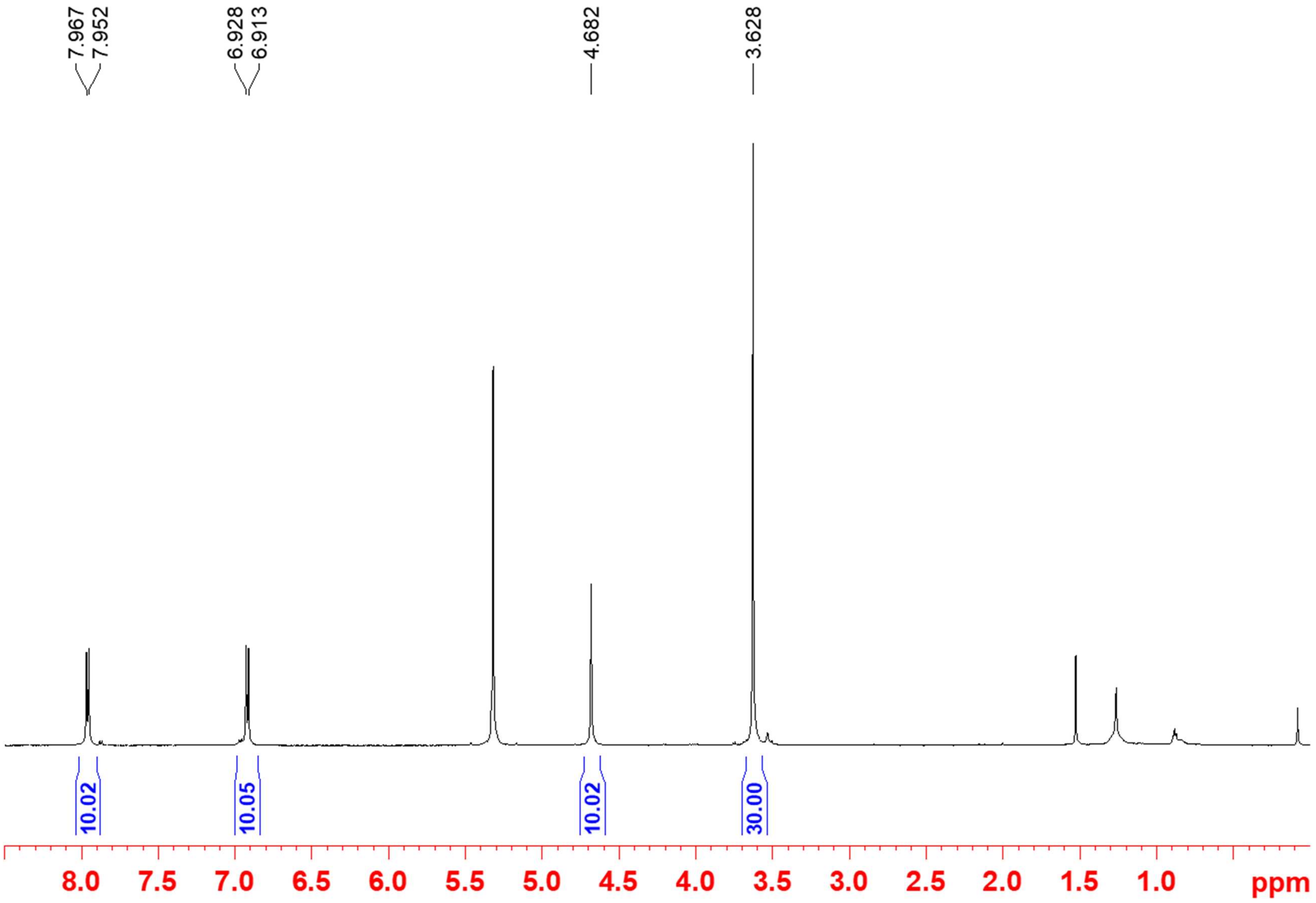

Figure S1: ^{1}H NMR spectrum of **1** (CD_2Cl_2, 600 MHz, 298 K).

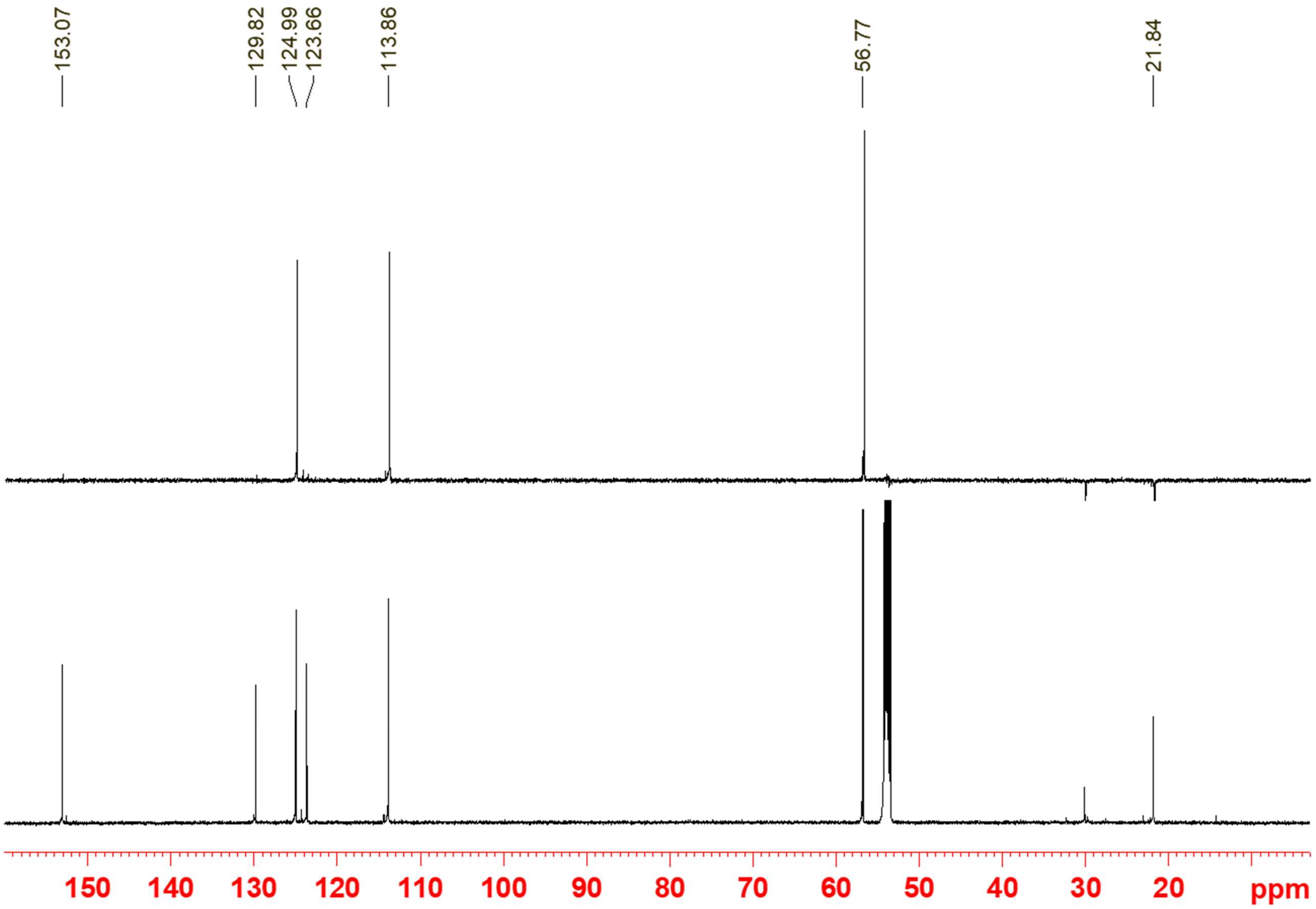

Figure S2: ^{13}C NMR and Dept-135 spectra of **1** (CD_2Cl_2, 150 MHz, 298 K).

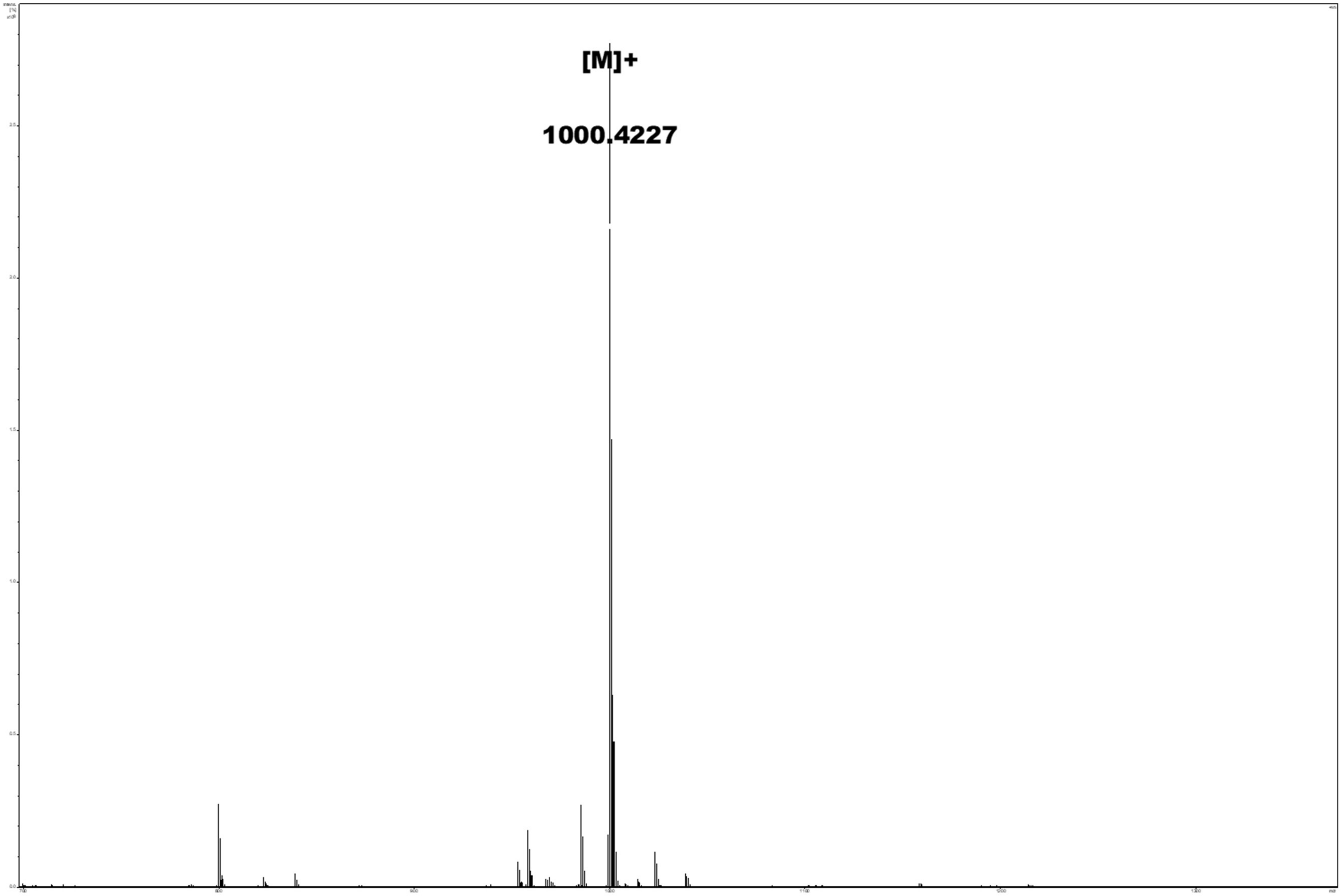

Figure S3: Significant portion of the HR MALDI FT-ICR mass spectrum of **1** $[M]^+$.

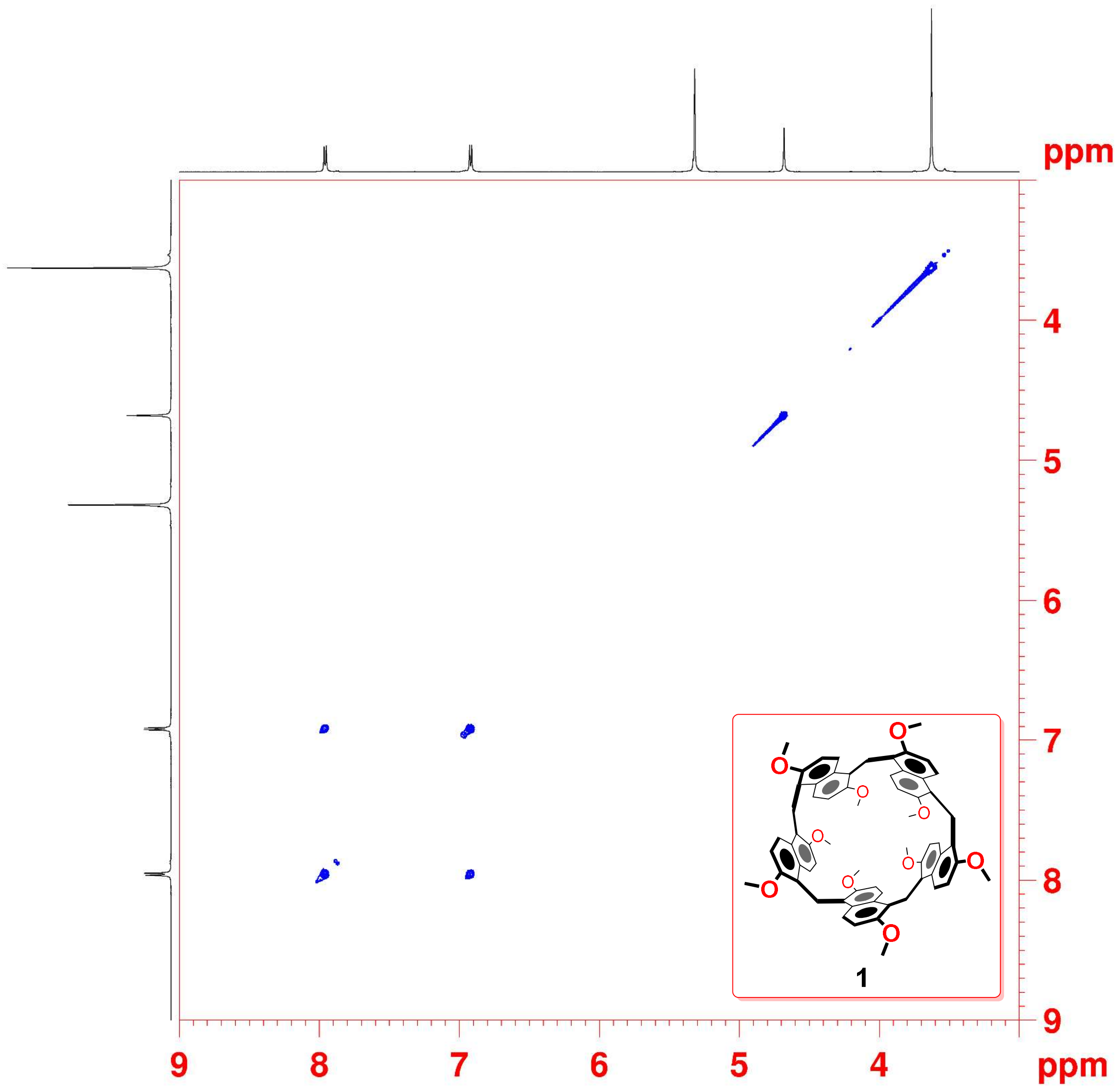

Figure S4: 2D-DQF COSY spectrum of **1** (CD_2Cl_2, 600 MHz, 298 K).

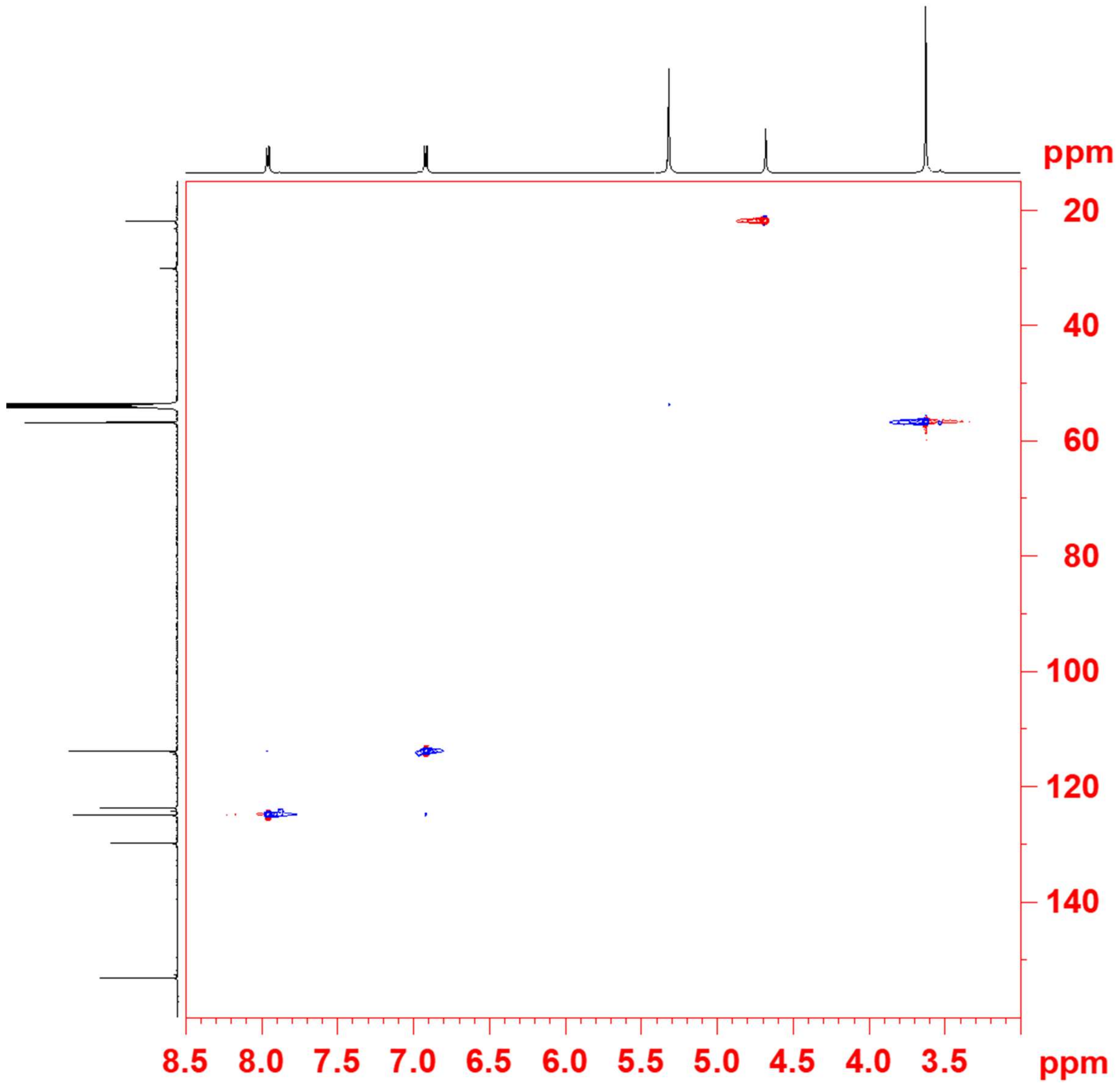

Figure S5: 2D-HSQC spectrum of **1** (CD_2Cl_2, 600 MHz, 298 K).

^{1}H NMR,^{13}C NMR and HR mass spectrum of derivatives 2

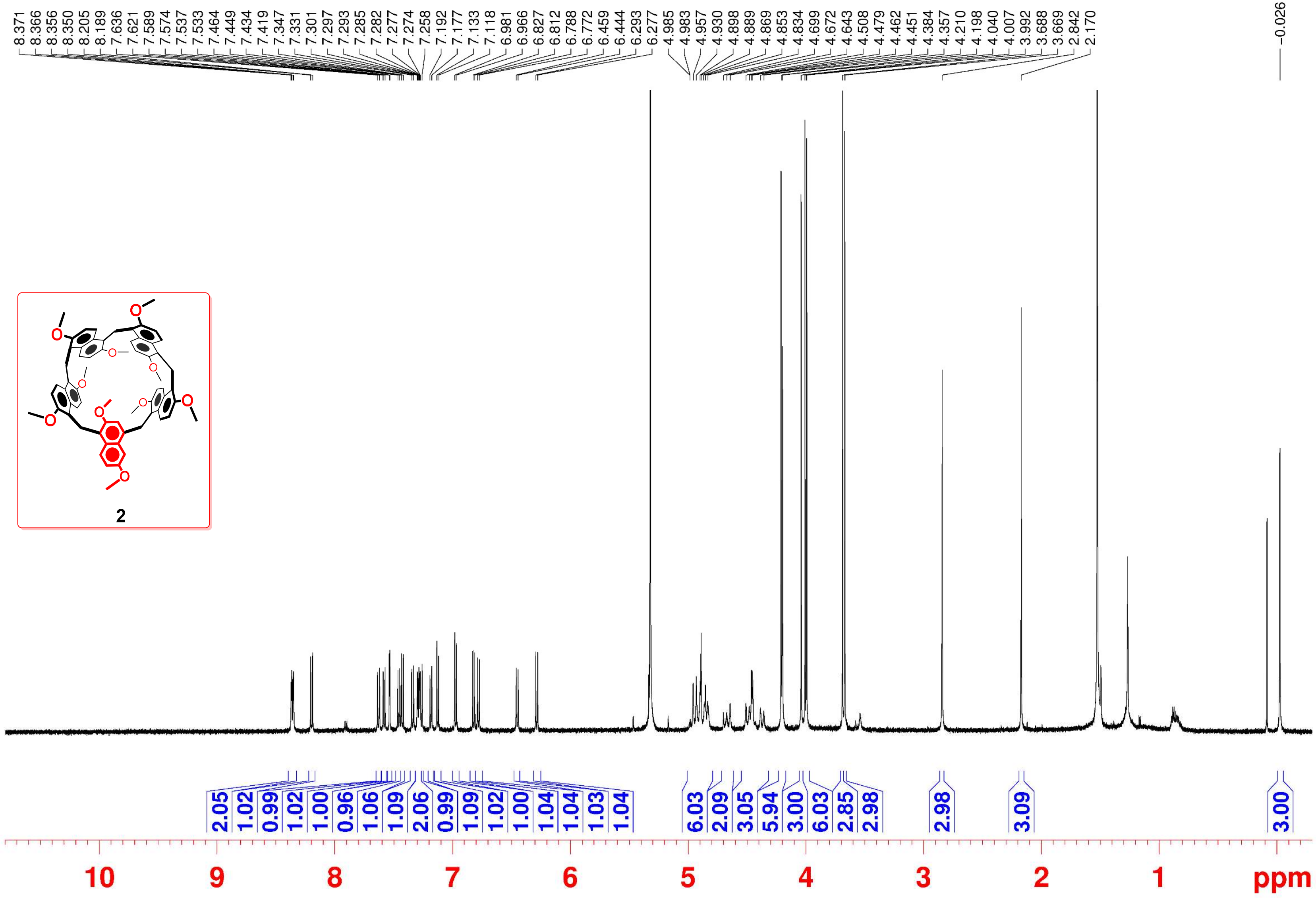

Figure S6: ^{1}H NMR spectrum of **2** (CD_2Cl_2, 600 MHz, 298 K).

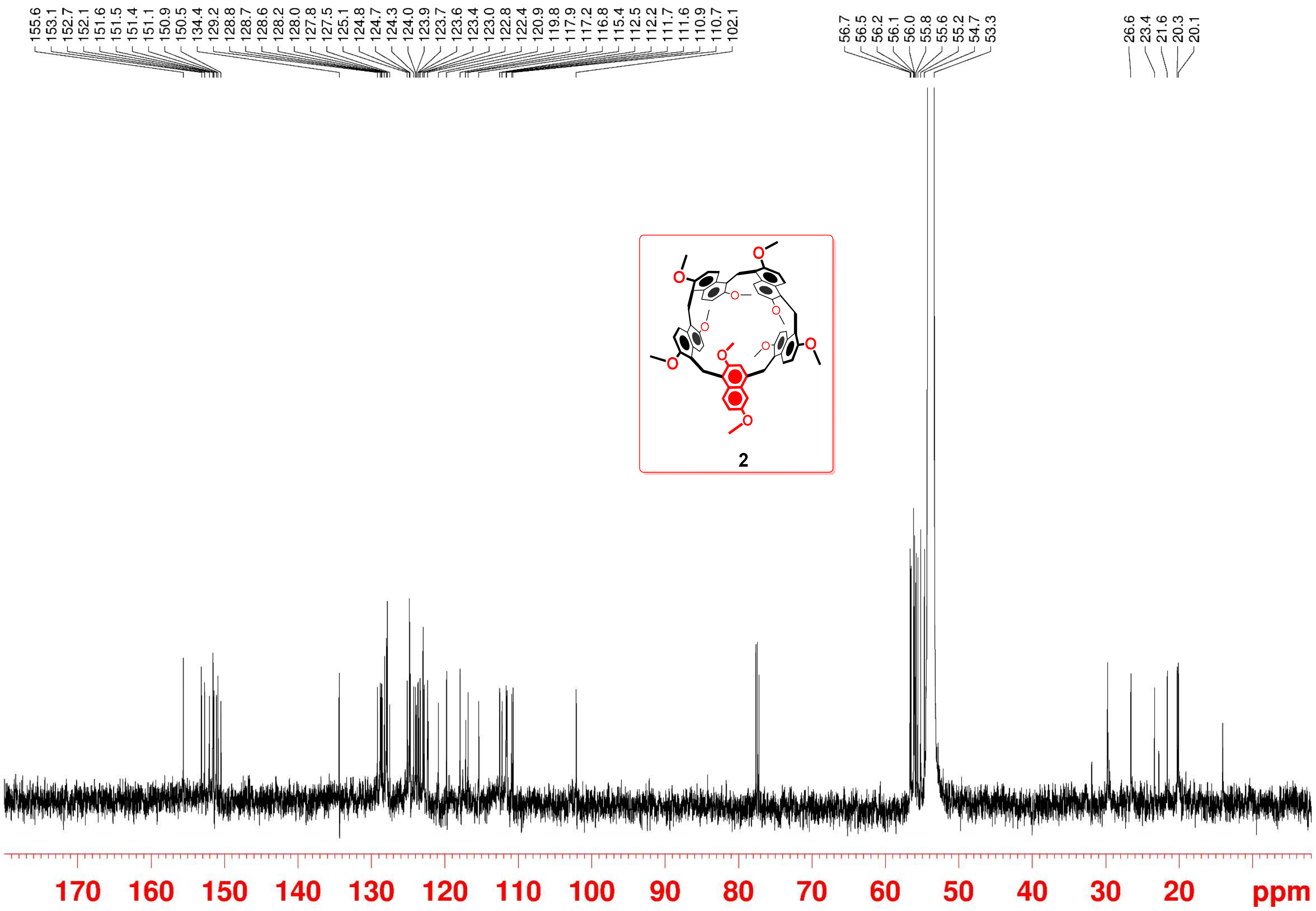

Figure S7: ^{13}C NMR spectrum of **2** (CD_2Cl_2, 150 MHz, 213 K).

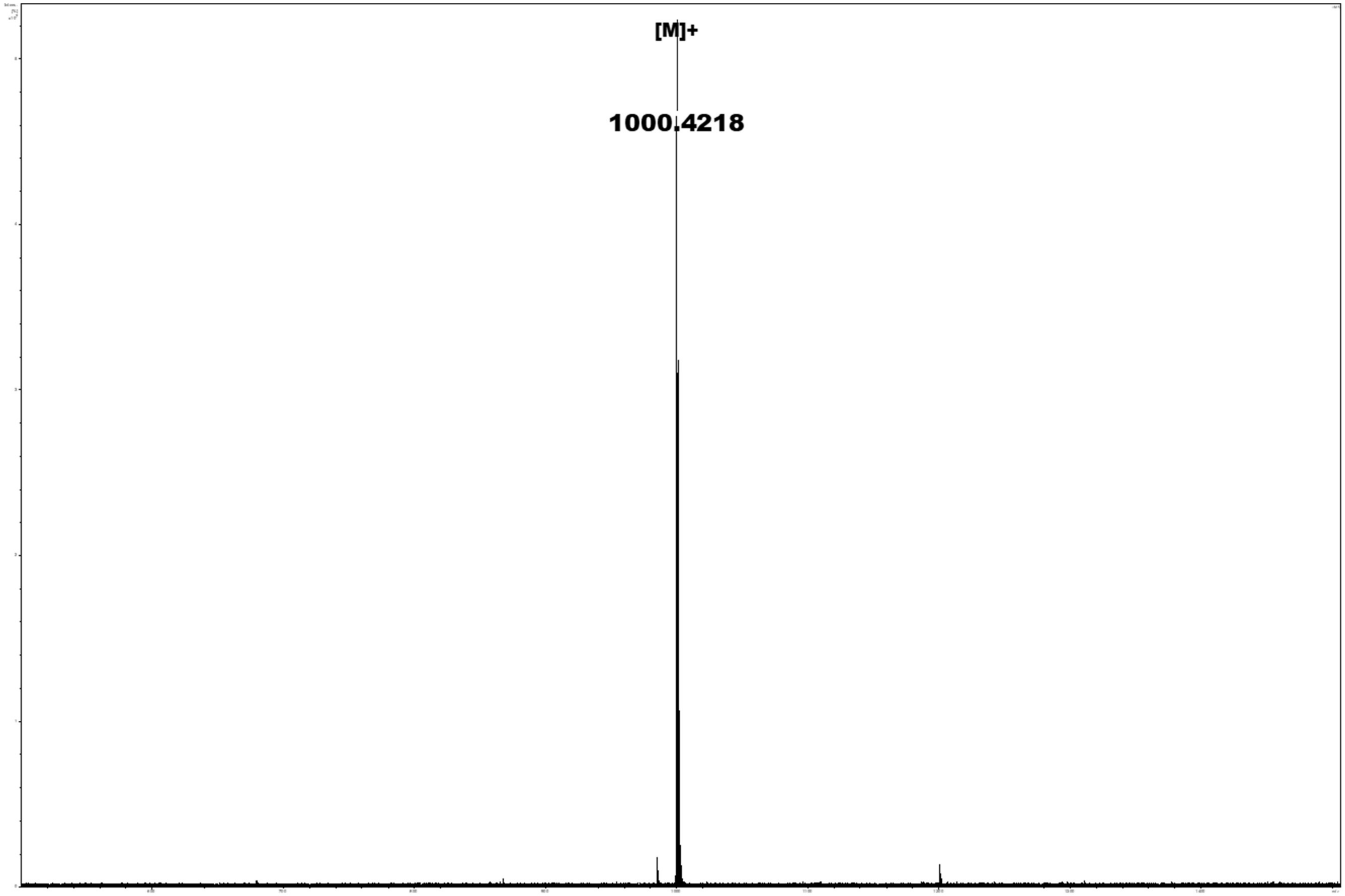

Figure S8: Significant portion of the HR MALDI FT-ICR mass spectrum of **2** $[M]^+$.

^{1}H VT NMR of 2

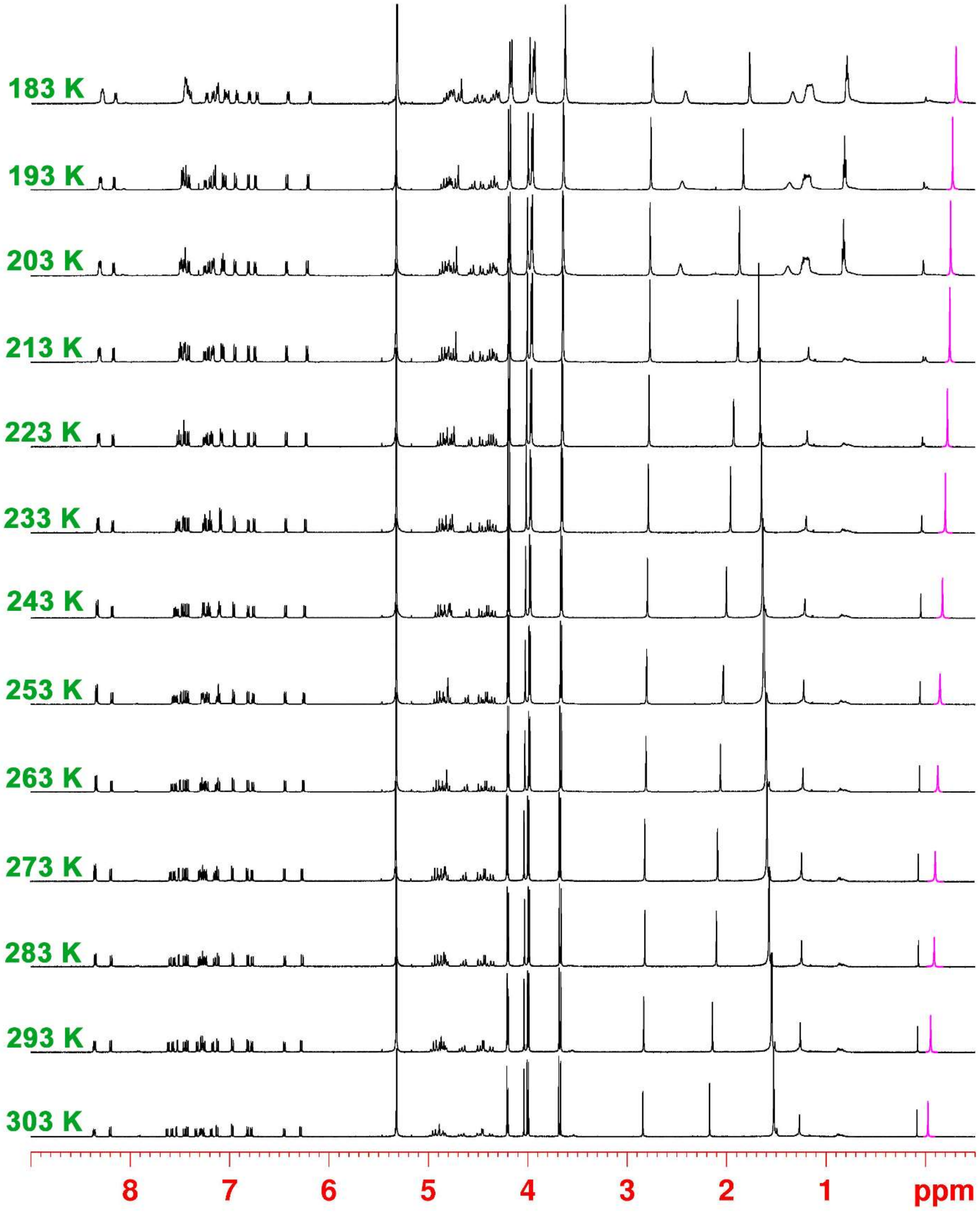

Figure S9: ^{1}H NMR spectrum of **2** (600 MHz, CD_2Cl_2) at (from bottom to top): 303, 293, 273, 263, 253, 243, 233, 223, 213, 203, 193, and 183 K.

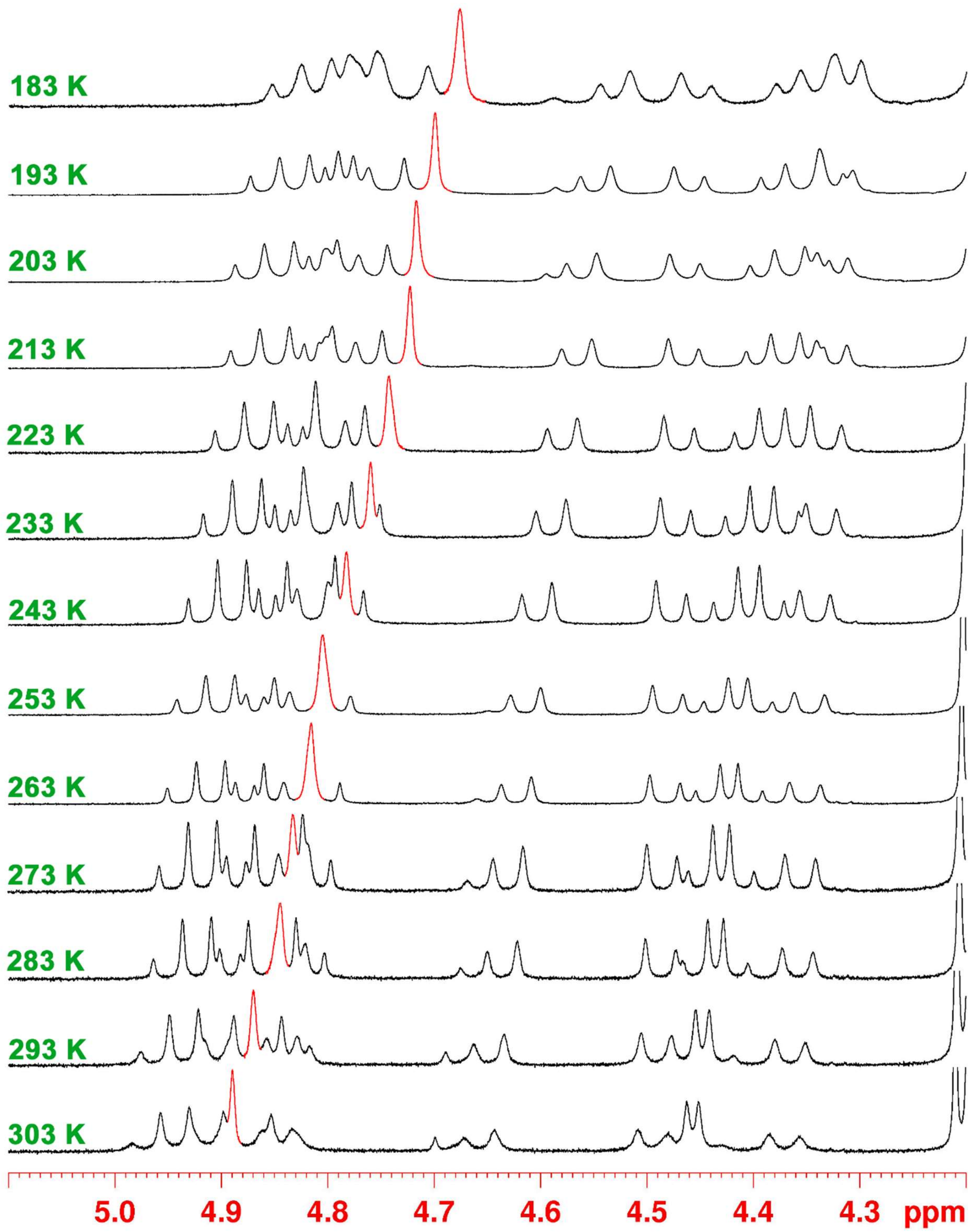

Figure S10: Relevant region of the ^{1}H NMR spectrum of **2** (600 MHz, CD_2Cl_2) at (from bottom to top): 303, 293, 273, 263, 253, 243, 233, 223, 213, 203, 193, and 183 K.

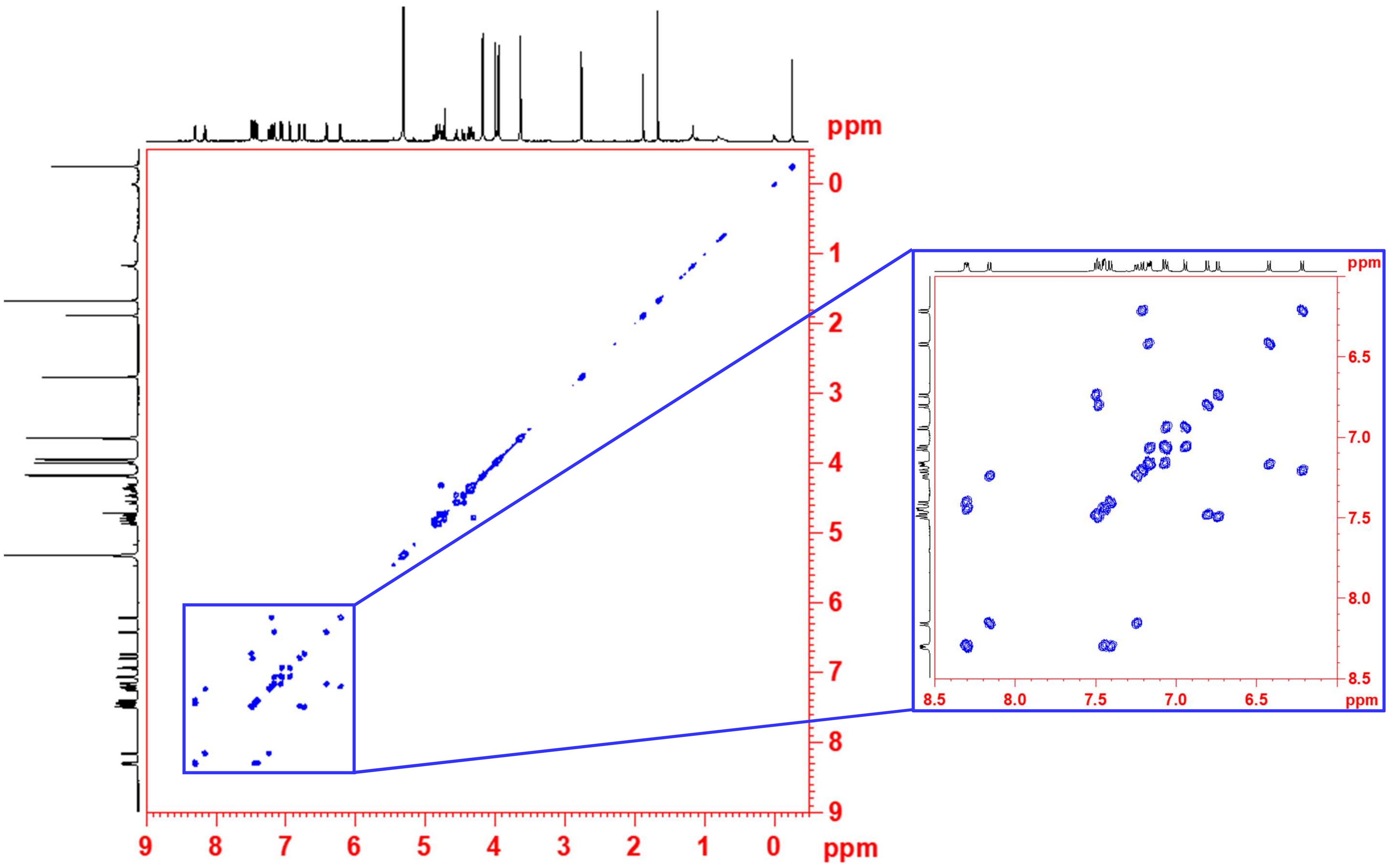

Figure S11: 2D-DQF COSY spectrum of **2** (CD_2Cl_2, 600 MHz, 213 K).

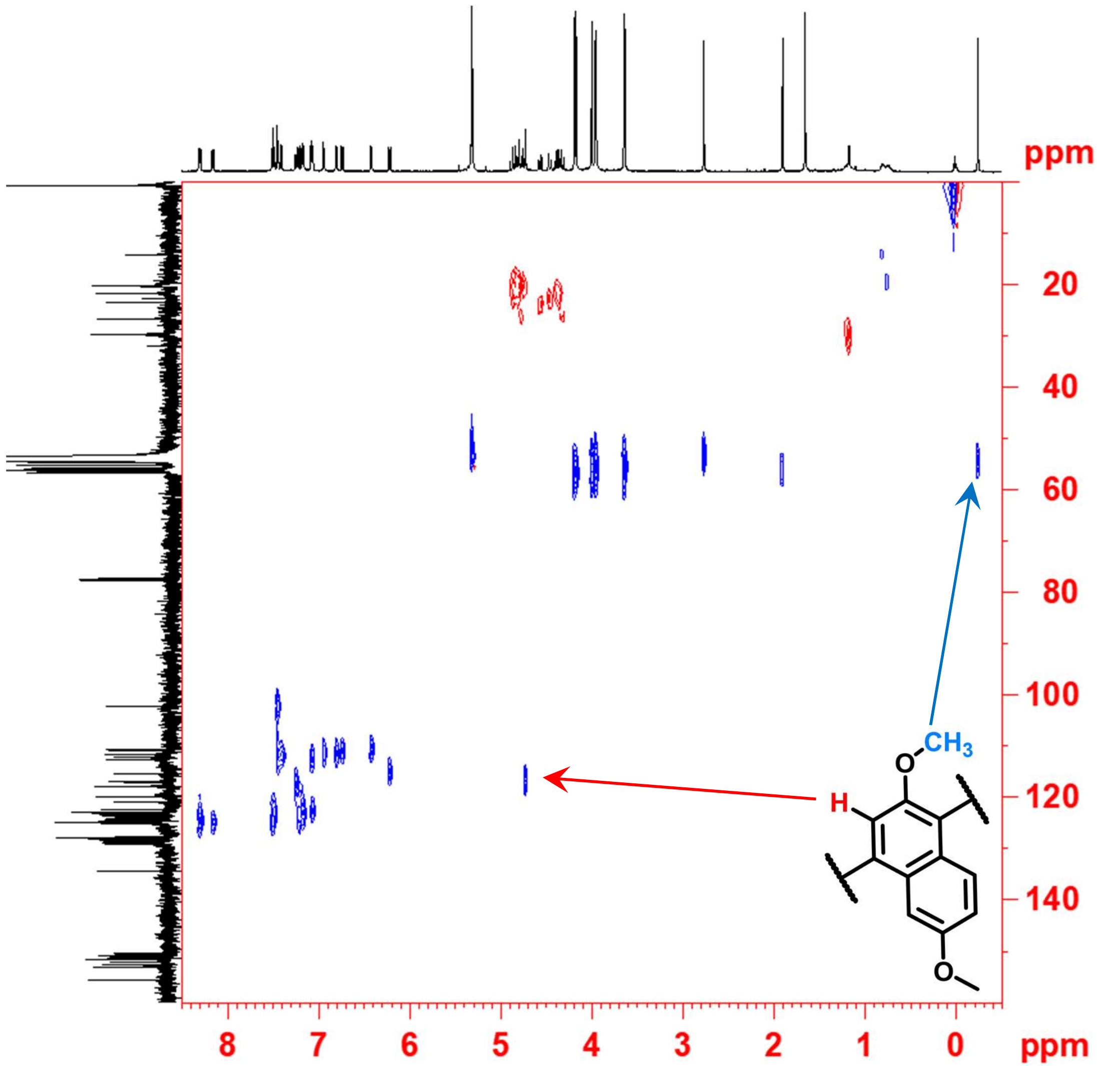

Figure S12: Significant portion of 2D-HSQC spectrum of **2** (CD_2Cl_2, 600 MHz, 213 K).

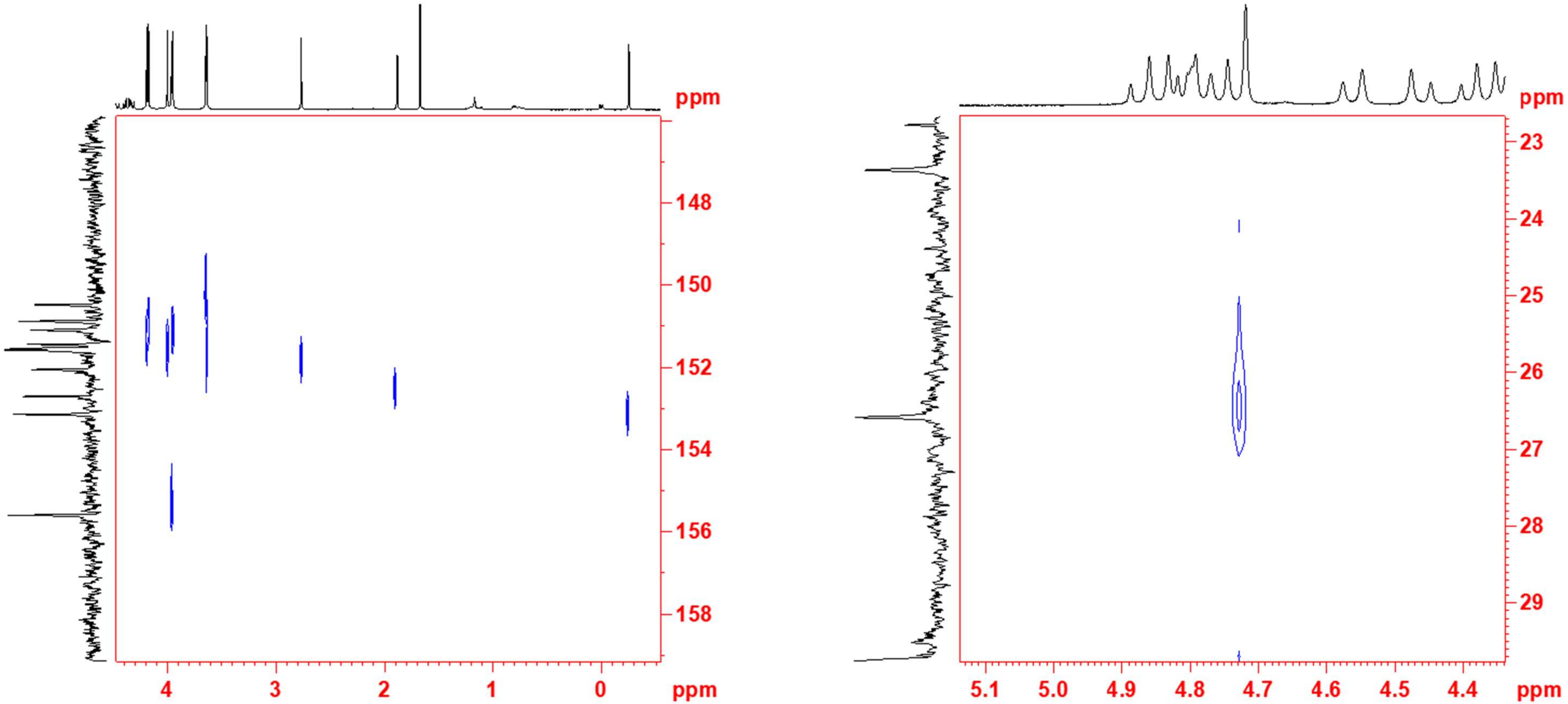

Figure S13: Significant portions of 2D-HMBC spectrum of **2** (CD_2Cl_2, 600 MHz, 213 K).

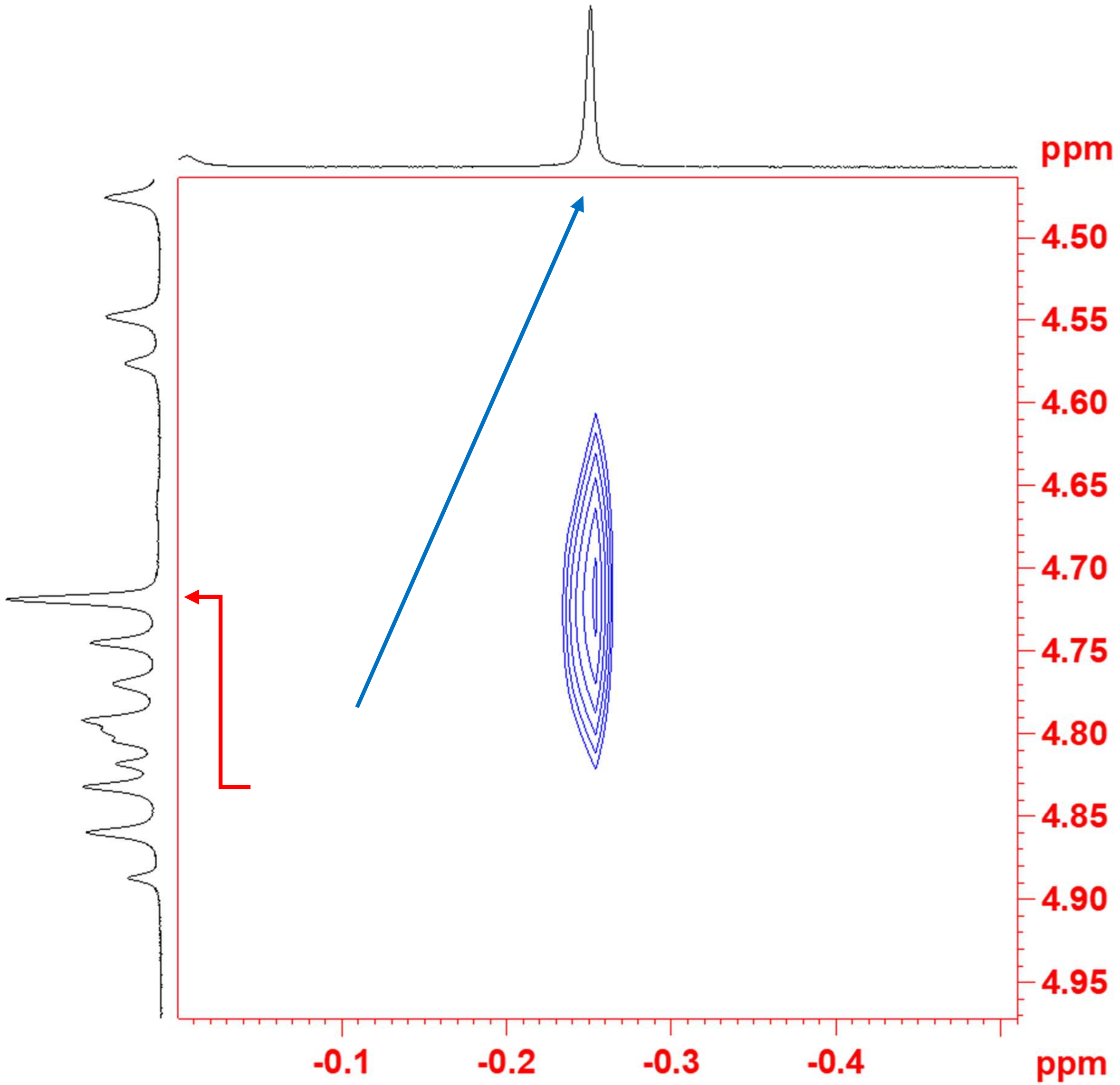

Figure S14: Significant portion of NOESY spectrum of **2** (CD_2Cl_2, 600 MHz, 213 K).

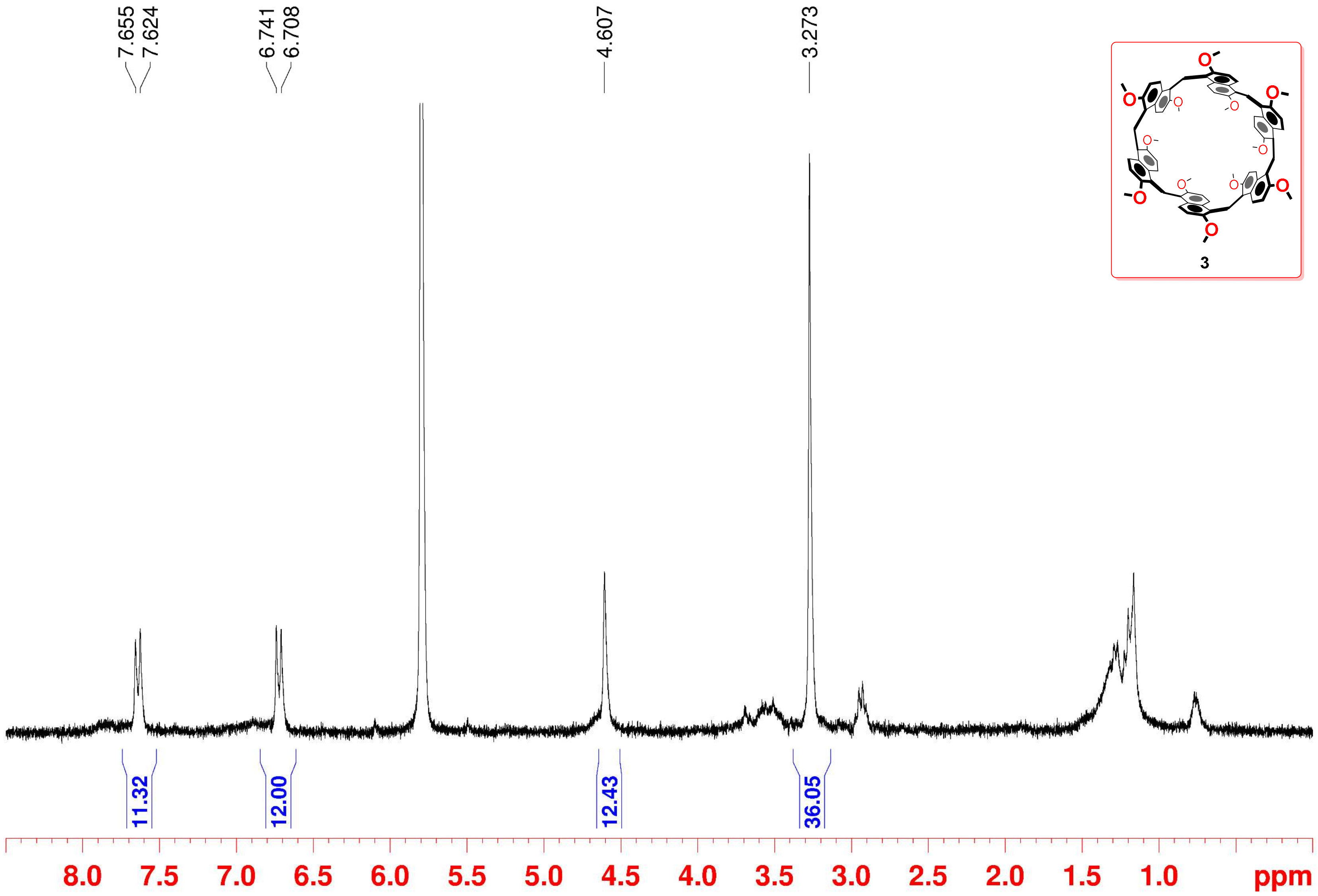

Figure S15: ^{1}H NMR spectrum of **3** (TCDE, 300 MHz, 393 K).

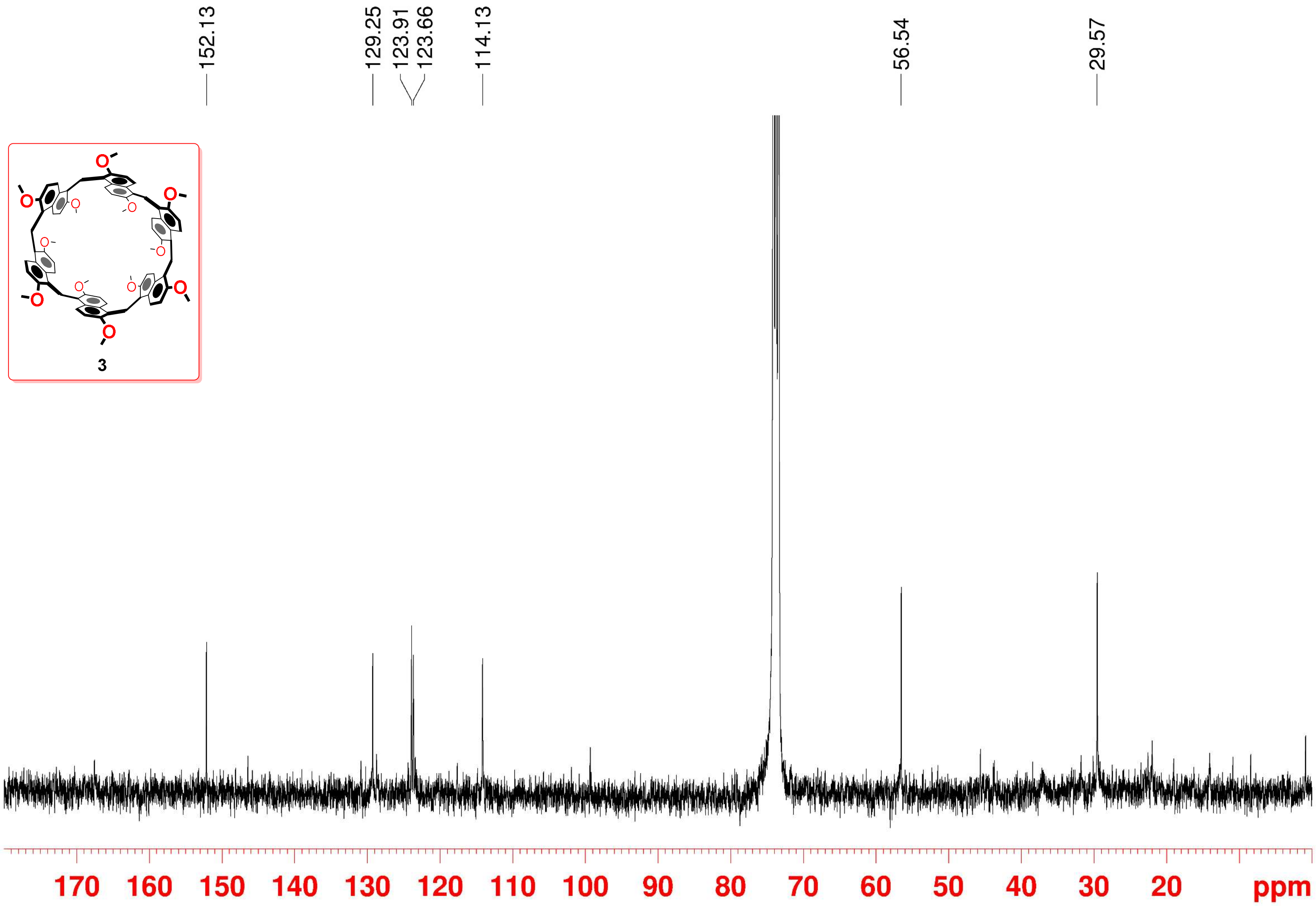

Figure S16: ^{13}C NMR spectrum of **3** (TCDE, 75 MHz, 298 K).

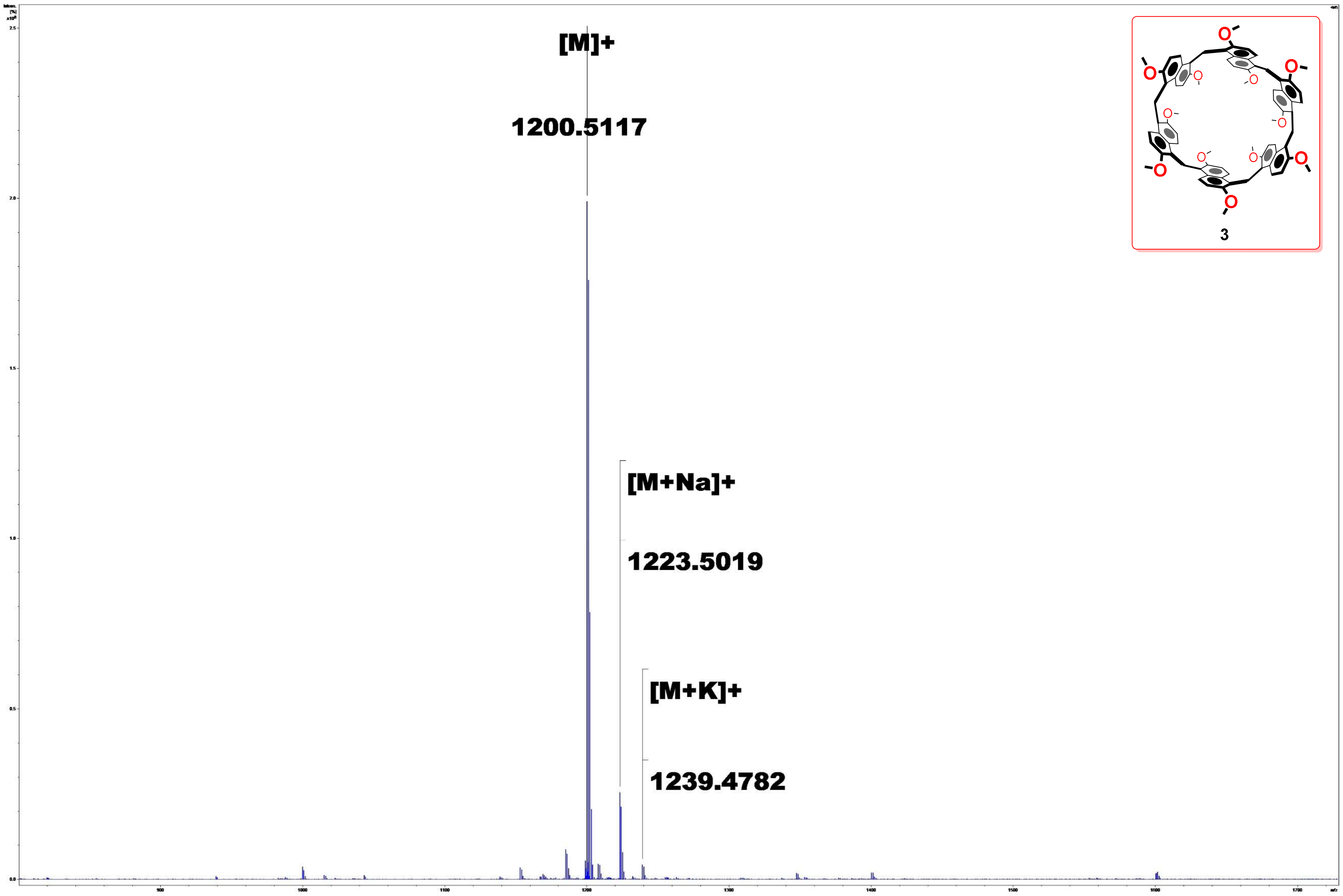

Figure S17: Significant portion of the HR MALDI FT-ICR mass spectrum of **3** $[M]^+$.

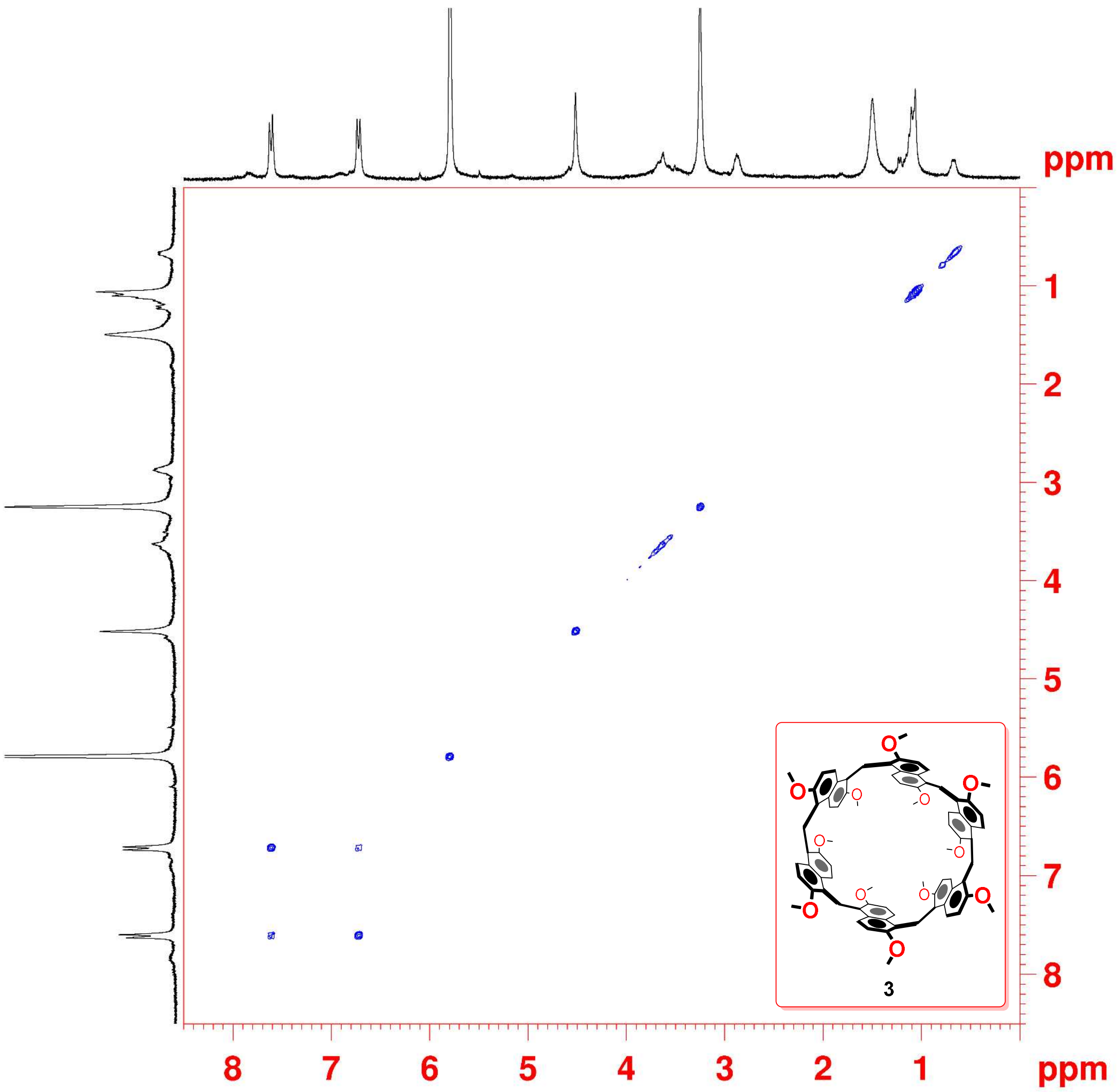

Figure S18: 2D-DQF COSY spectrum of **3** (TCDE, 400 MHz, 298 K).

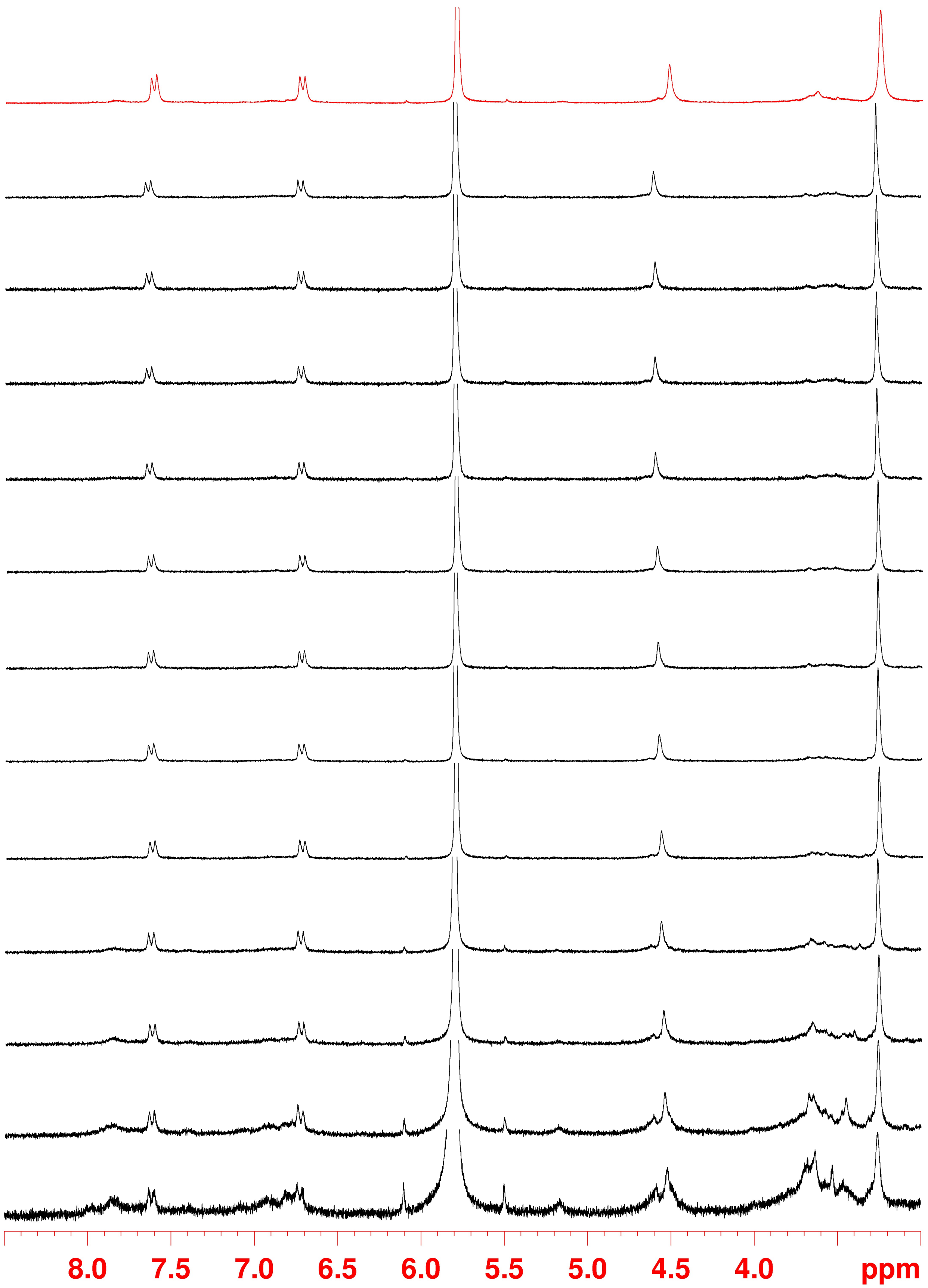

Figure S19: Relevant region of the ^{1}H NMR spectrum of **3** (300 MHz, TCDE) at (from bottom to top): 303, 313, 323, 333, 343, 353, 363, 373, 383 and 393 K; in red the spectrum at 298 K after high temperature acquisition.

Copies of 1D, 2D and HR Mass Spectra of Prismarene Complexes

Copies of NMR and HR Mass Spectra of $4^{2+}\subset$ 1

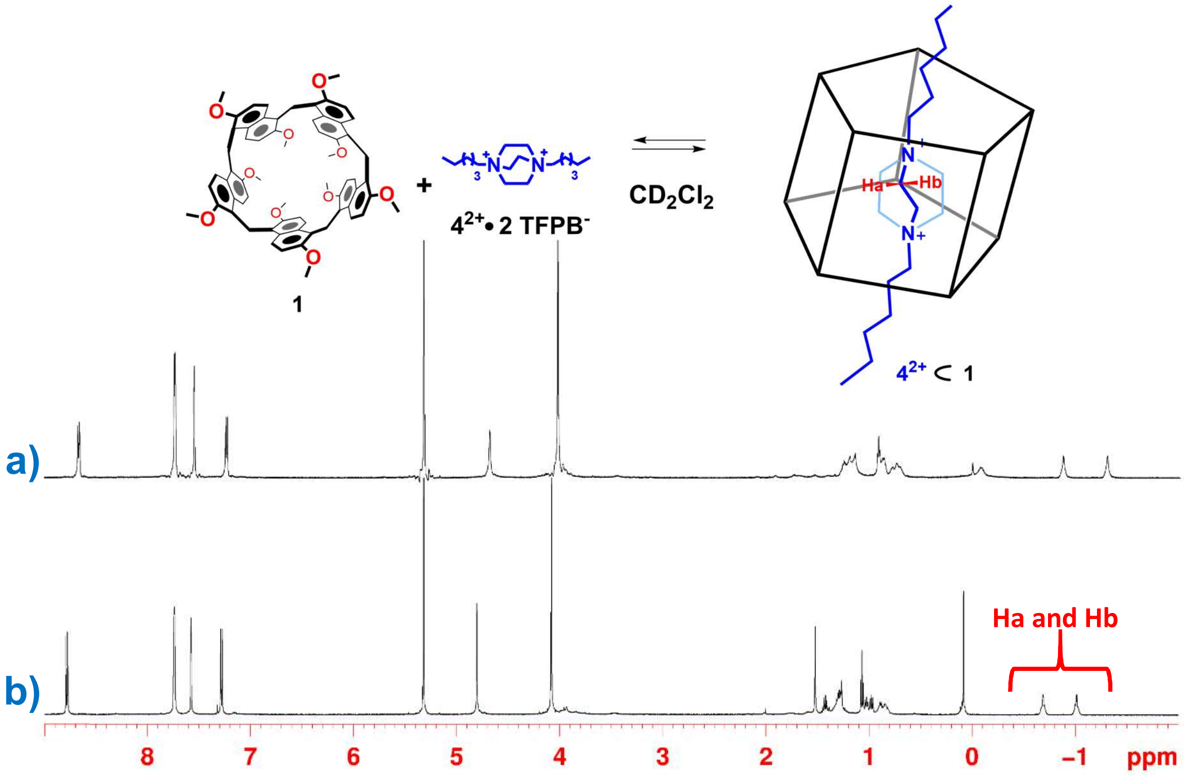

Figure S20: ^{1}H NMR spectra of an equimolar solution of **1** and **4^{2+}· 2 TFPB^{-}** (a) at 183 K and (b) at 298 K (600 MHz, CD_2Cl_2).

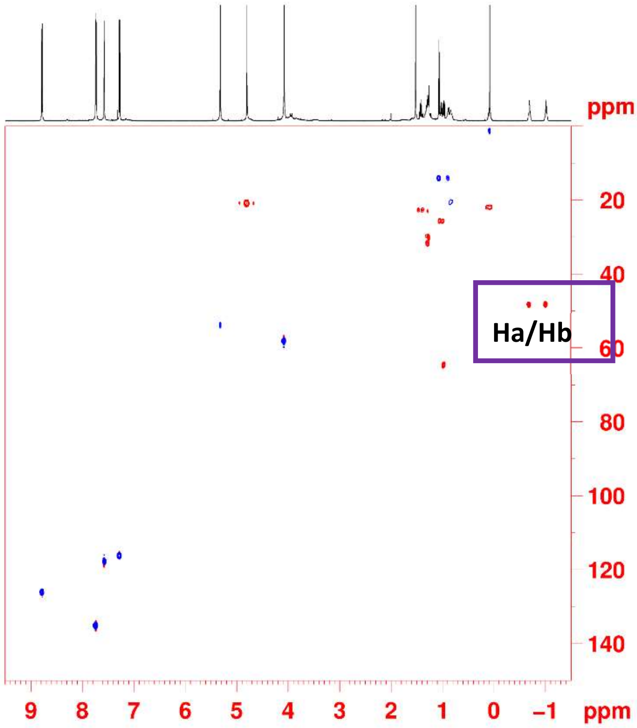

Figure S21: 2D-HSQC spectrum of **$4^{2+}\subset$ 1** (CD_2Cl_2, 600 MHz, 298 K).

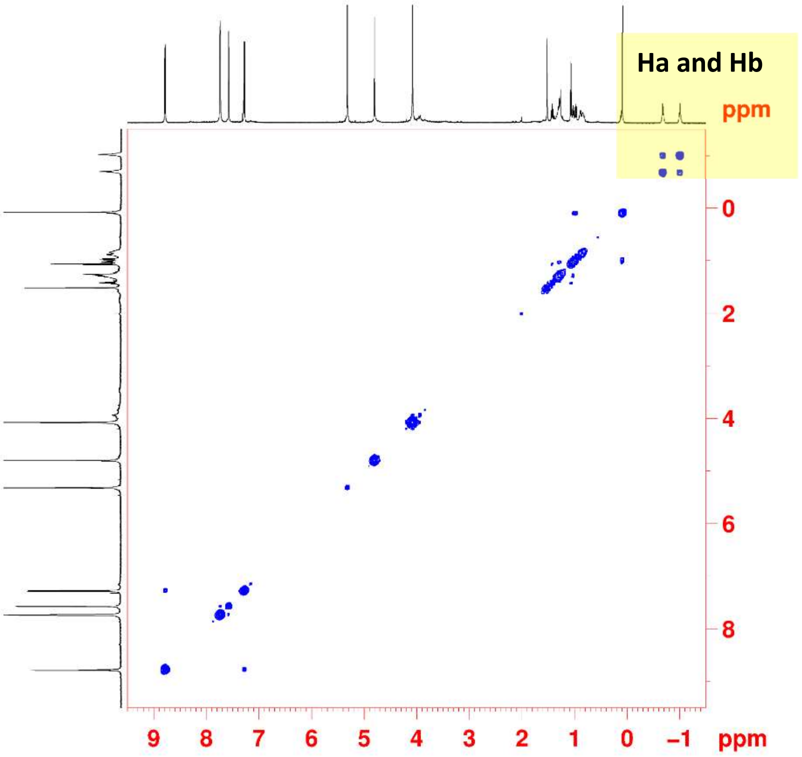

Figure S22: 2D-DQF COSY spectrum of **4**$^{2+}$$\subset$ **1** (CD_2Cl_2, 600 MHz, 298 K).

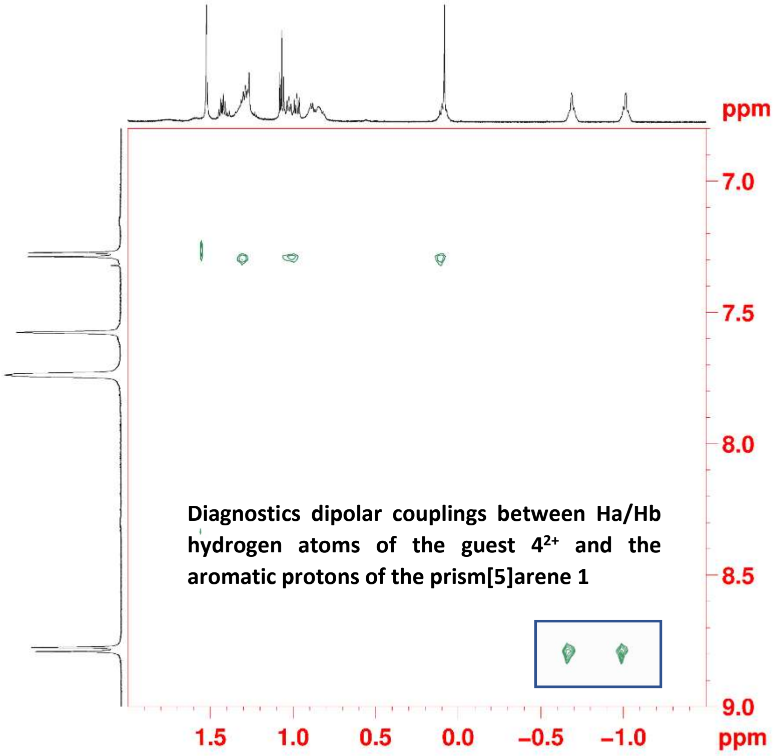

Figure S23: Significant portion of 2D-NOESY spectrum of **4**$^{2+}$$\subset$ **1** (CD_2Cl_2, 600 MHz, 298 K).

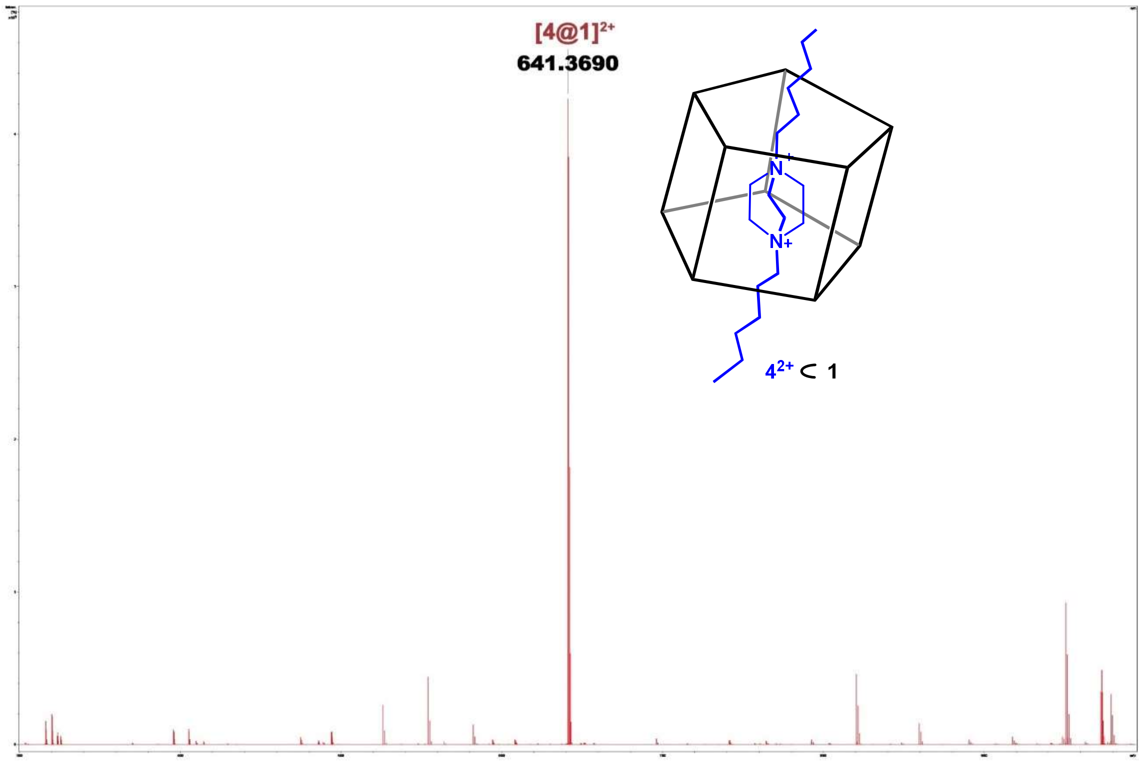

Figure S24: Significant portion of the HR ESI FT-ICR mass spectrum of **[4⊂ 1]** $^{2+}$.

Copies of NMR and HR Mass Spectra of $5^+ \subset 1$

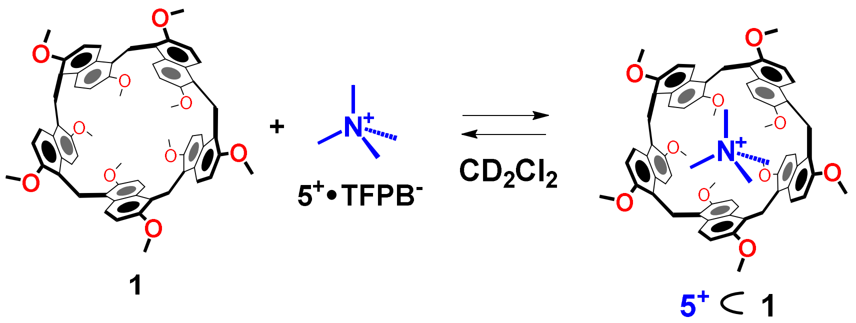

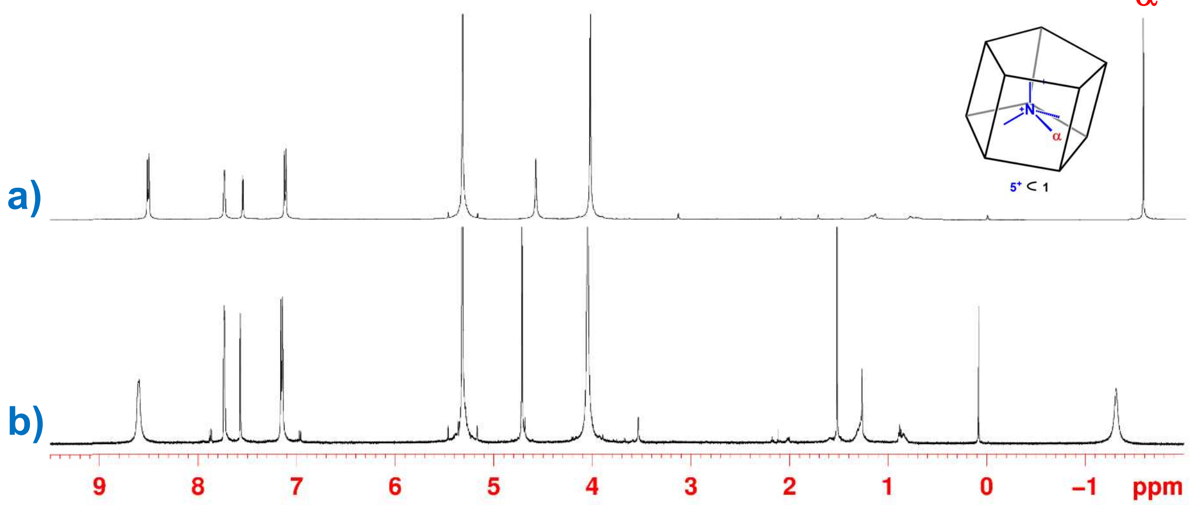

Figure S25: ^{1}H NMR spectra of an equimolar solution of **1** and $\mathbf{5^+\cdot}$ **TFPB**$^-$ (a) at 183 K and (b) at 298 K (600 MHz, CD_2Cl_2).

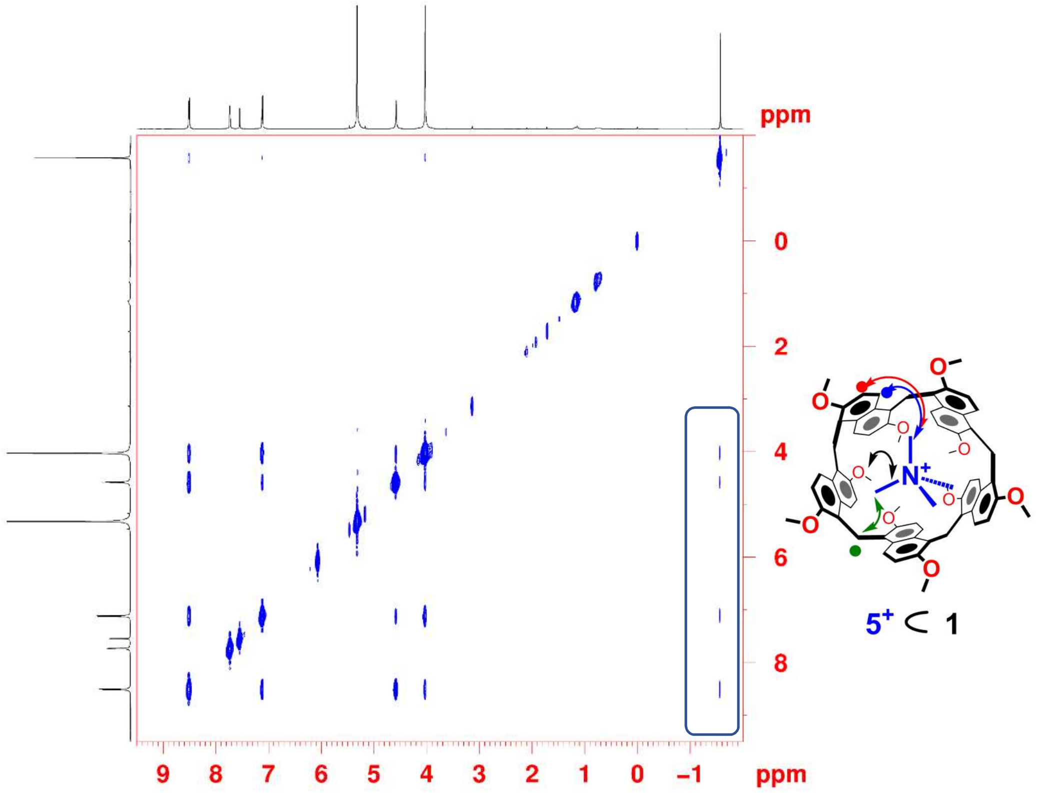

Figure S26: 2D-NOESY spectrum of $\mathbf{5^+ \subset 1}$ (CD_2Cl_2, 600 MHz, 183 K).

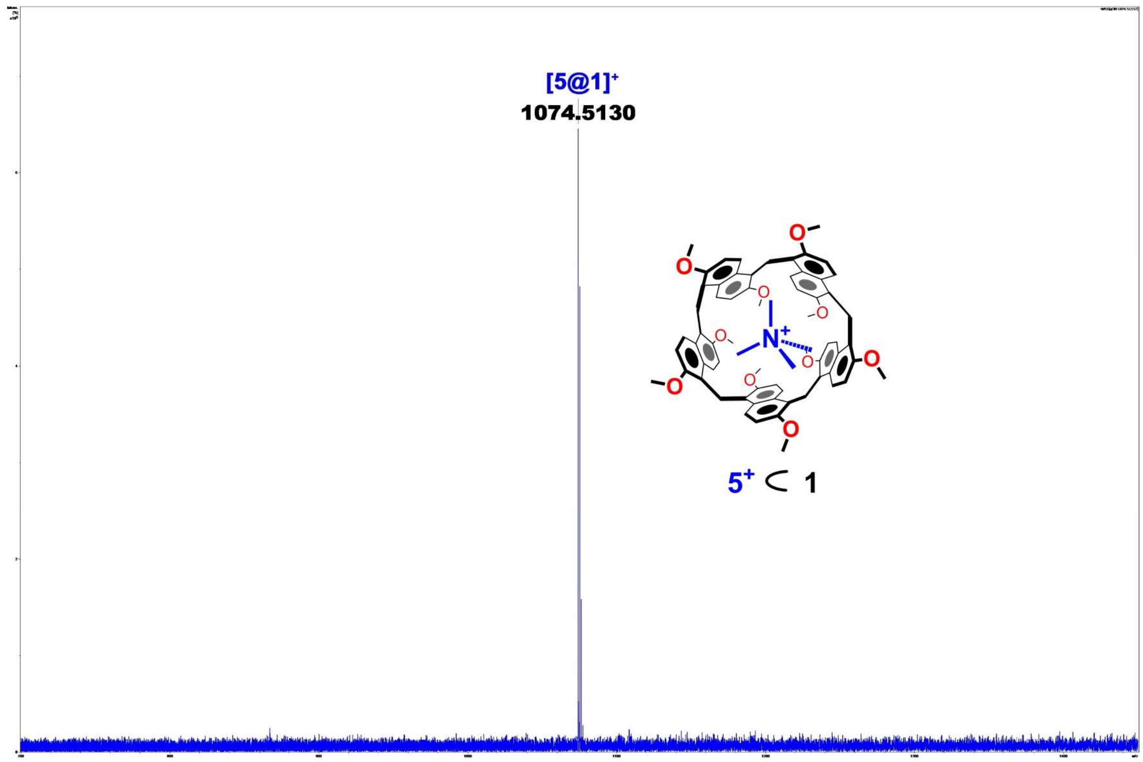

Figure S27: Significant portion of the HR ESI FT-ICR mass spectrum of **[5⊂1]** $^+$.

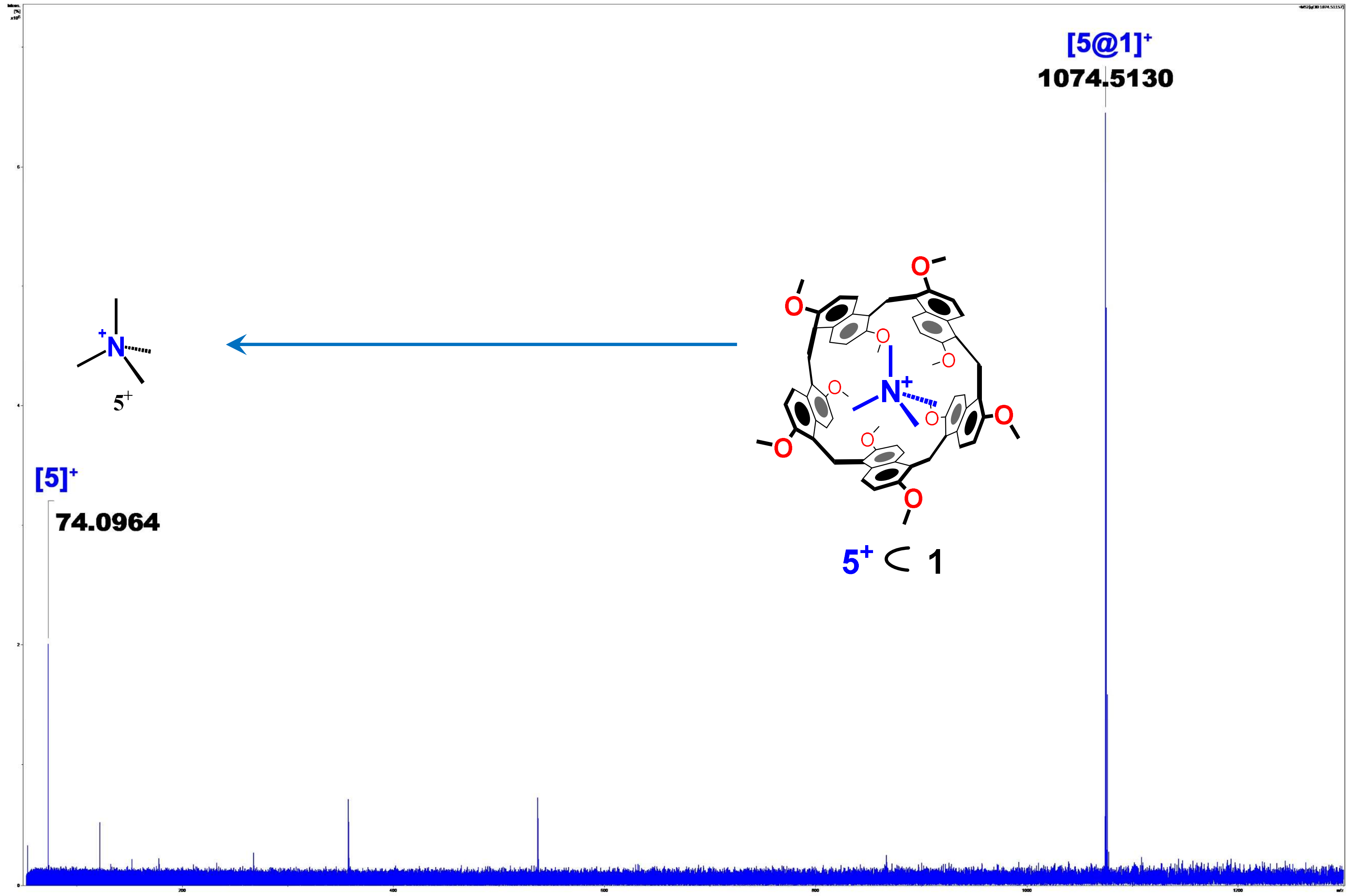

Figure S28: HR ESI-FT-ICR-CID mass spectrum of **[5⊂ 1]** ⁺.

Copies of NMR and HR Mass Spectra of $6^{+}\subset 1$

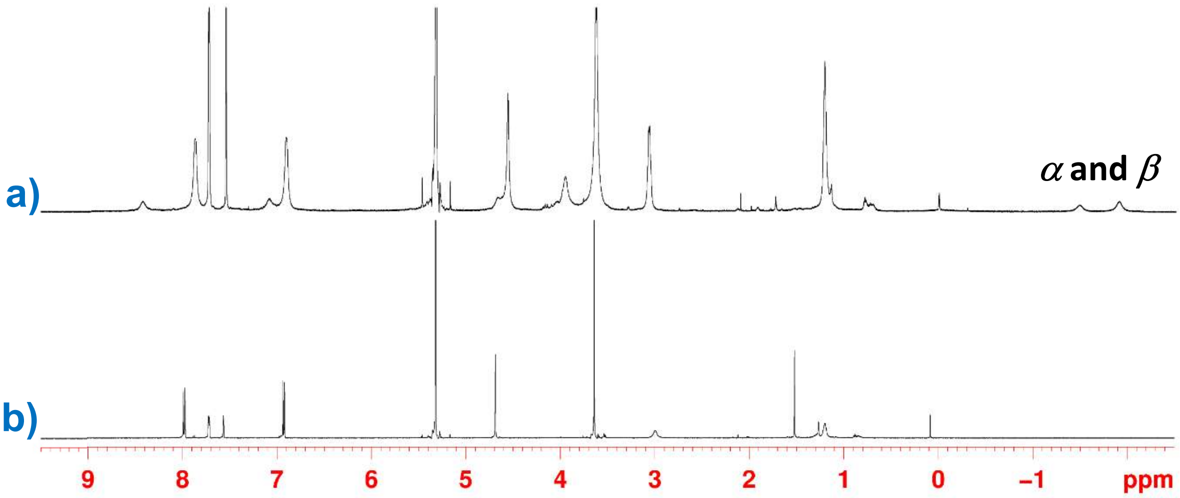

Figure S29: ^{1}H NMR spectra of an equimolar solution of **1** and **6^{+}· TFPB^{-}** (a) at 183 K and (b) at 298 K (600 MHz, CD_2Cl_2).

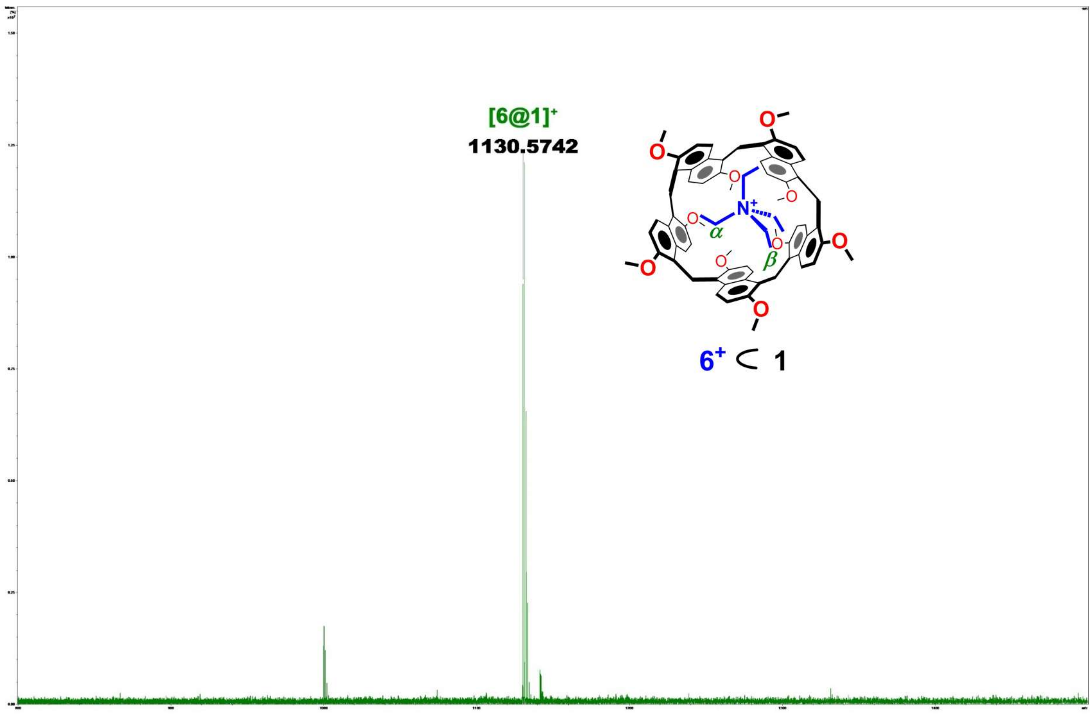

Figure S30: Significant portion of the HR ESI FT-ICR mass spectrum of **[6 ⊂ 1]**$^{+}$.

Copies of NMR and HR Mass Spectra of $7^{+} \subset 1$

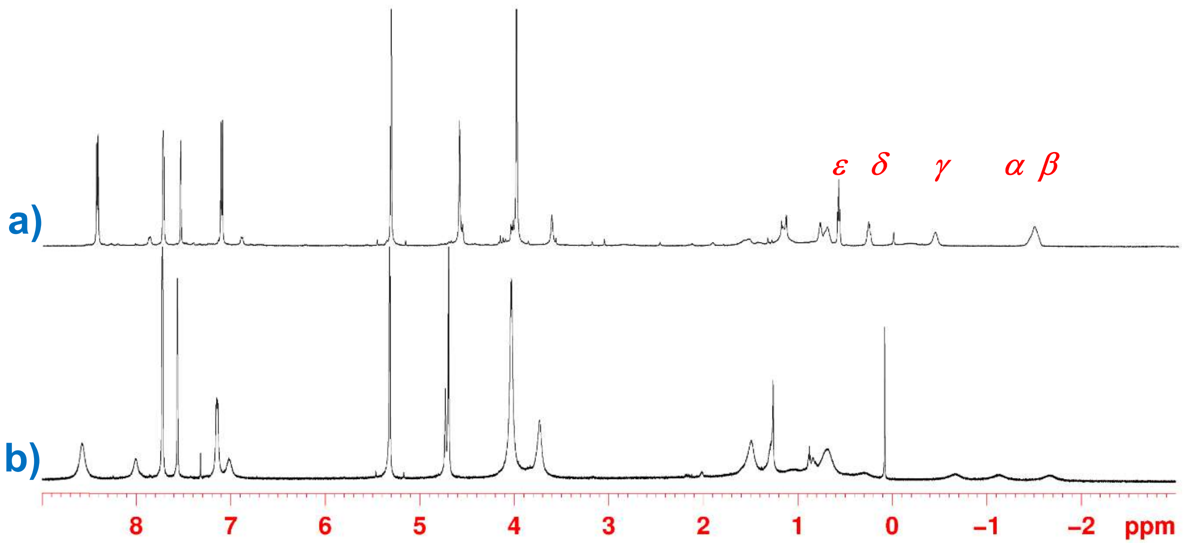

Figure S31: ^{1}H NMR spectra of an equimolar solution of **1** and **7^{+}· TFPB^{-}** (a) at 183 K and (b) at 298 K (600 MHz, CD_2Cl_2).

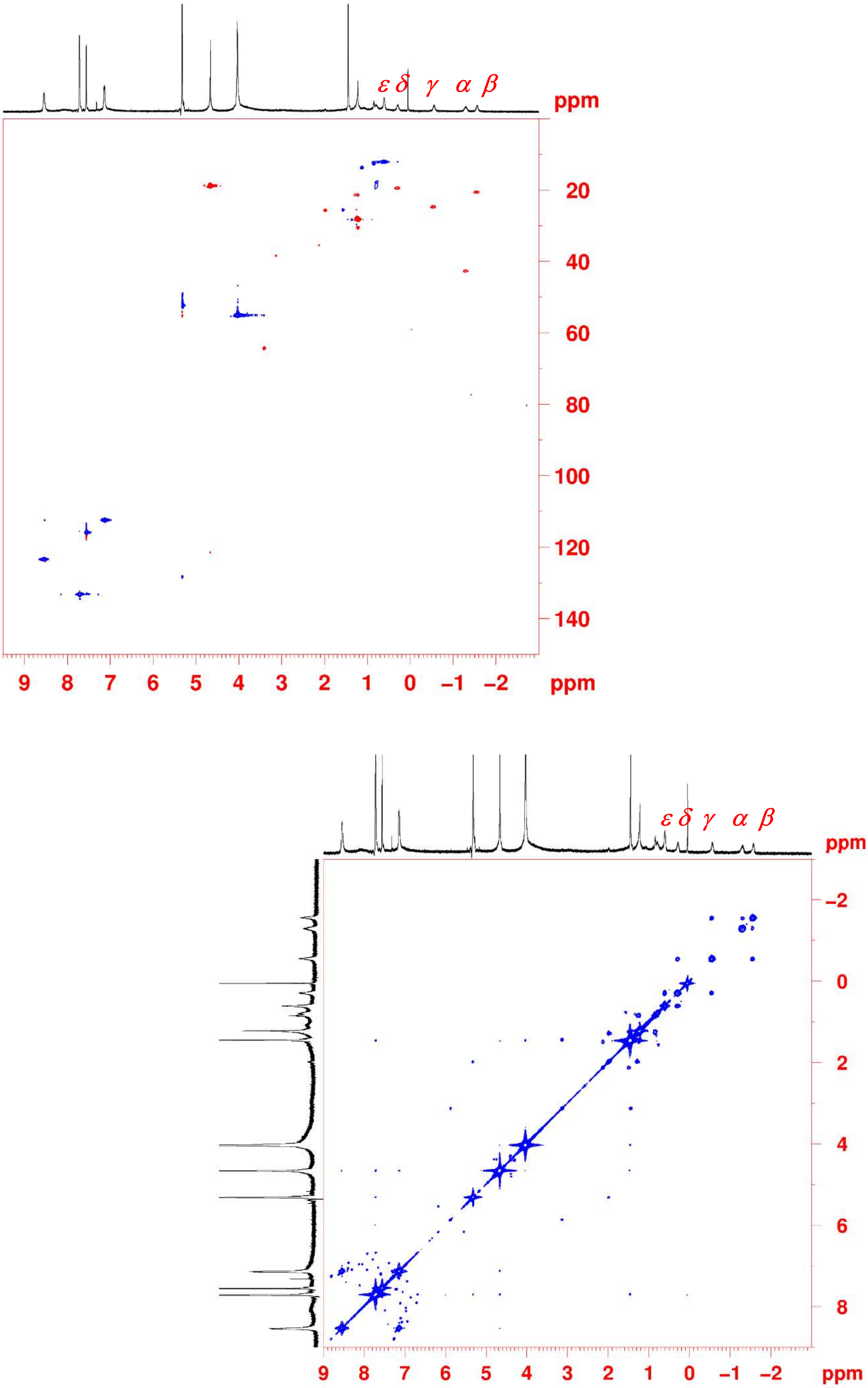

Figure S32: 2D-HSQC and 2D-DQF COSY spectra of $7^{+}\subset$ **1** (CD_2Cl_2, 600 MHz, 253 K).

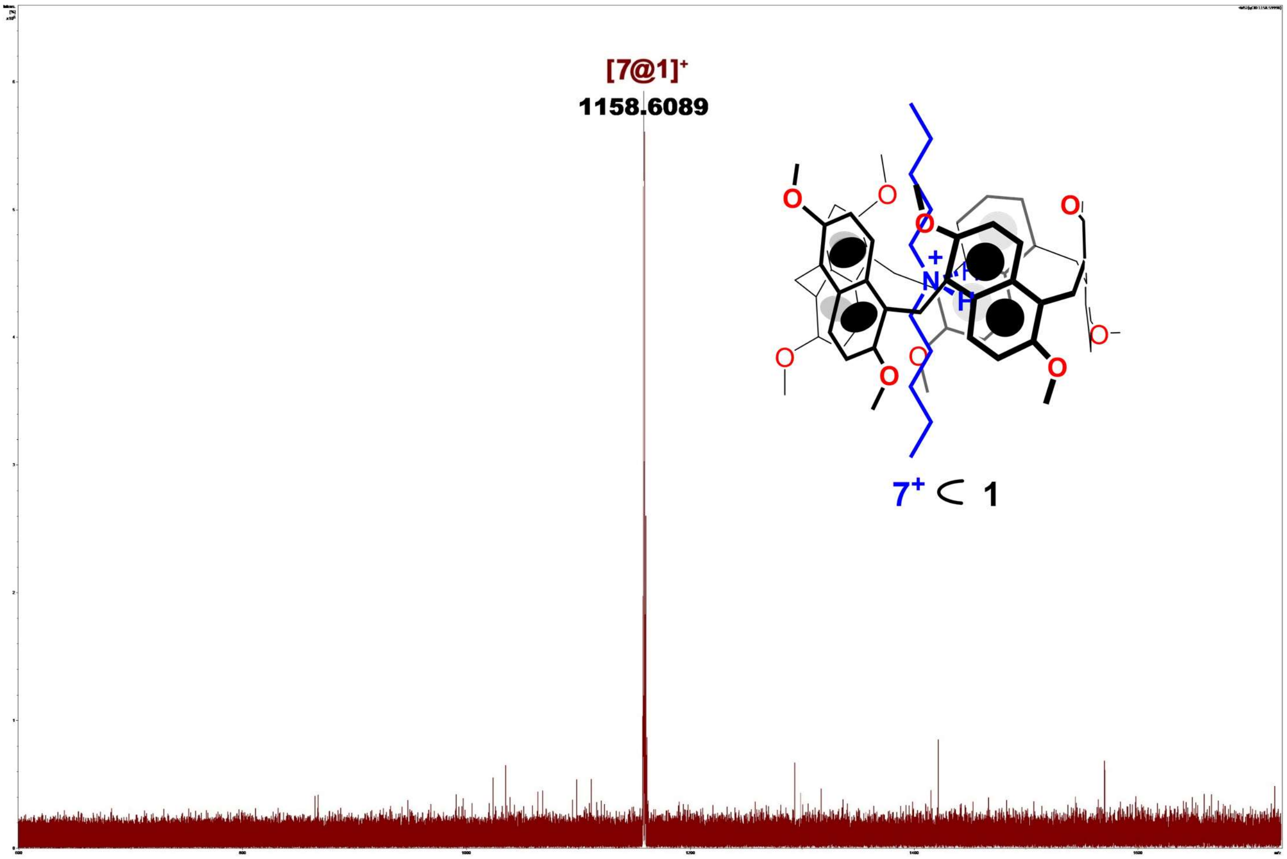

Figure S33: Significant portion of the HR ESI FT-ICR mass spectrum of **[7⊂1]** $^{+}$.

Copies of NMR and HR Mass Spectra of $8^{+}\subset 1$

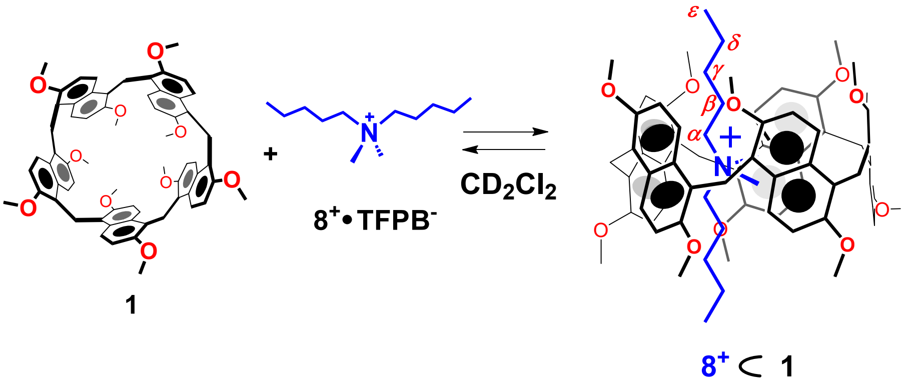

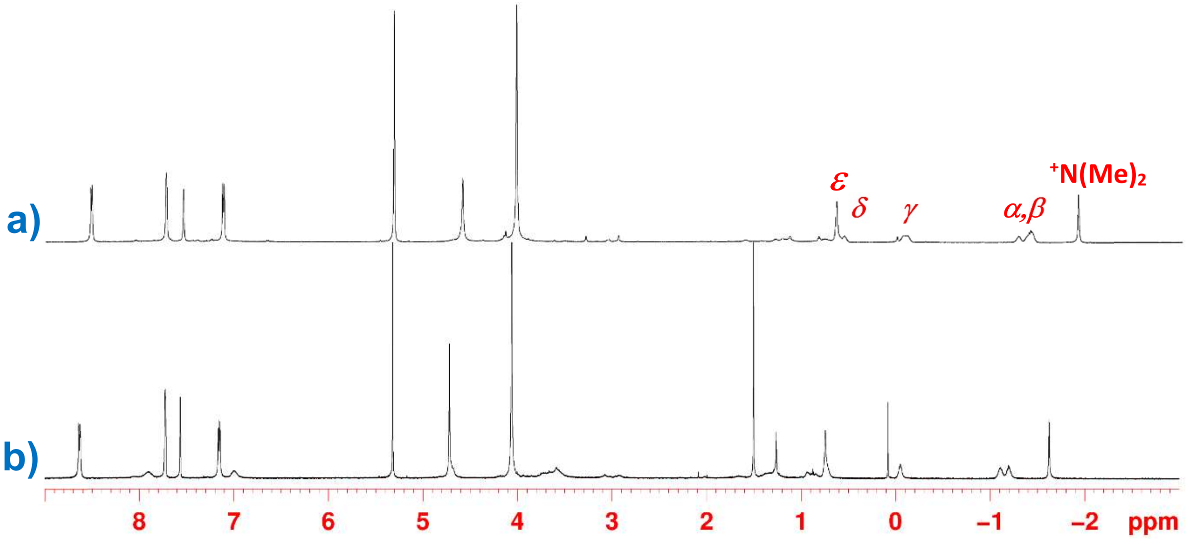

Figure S34: ^{1}H NMR spectra of an equimolar solution of **1** and **$8^{+}\cdot$ TFPB^{-}** (a) at 183 K and (b) at 298 K (600 MHz, CD_2Cl_2).

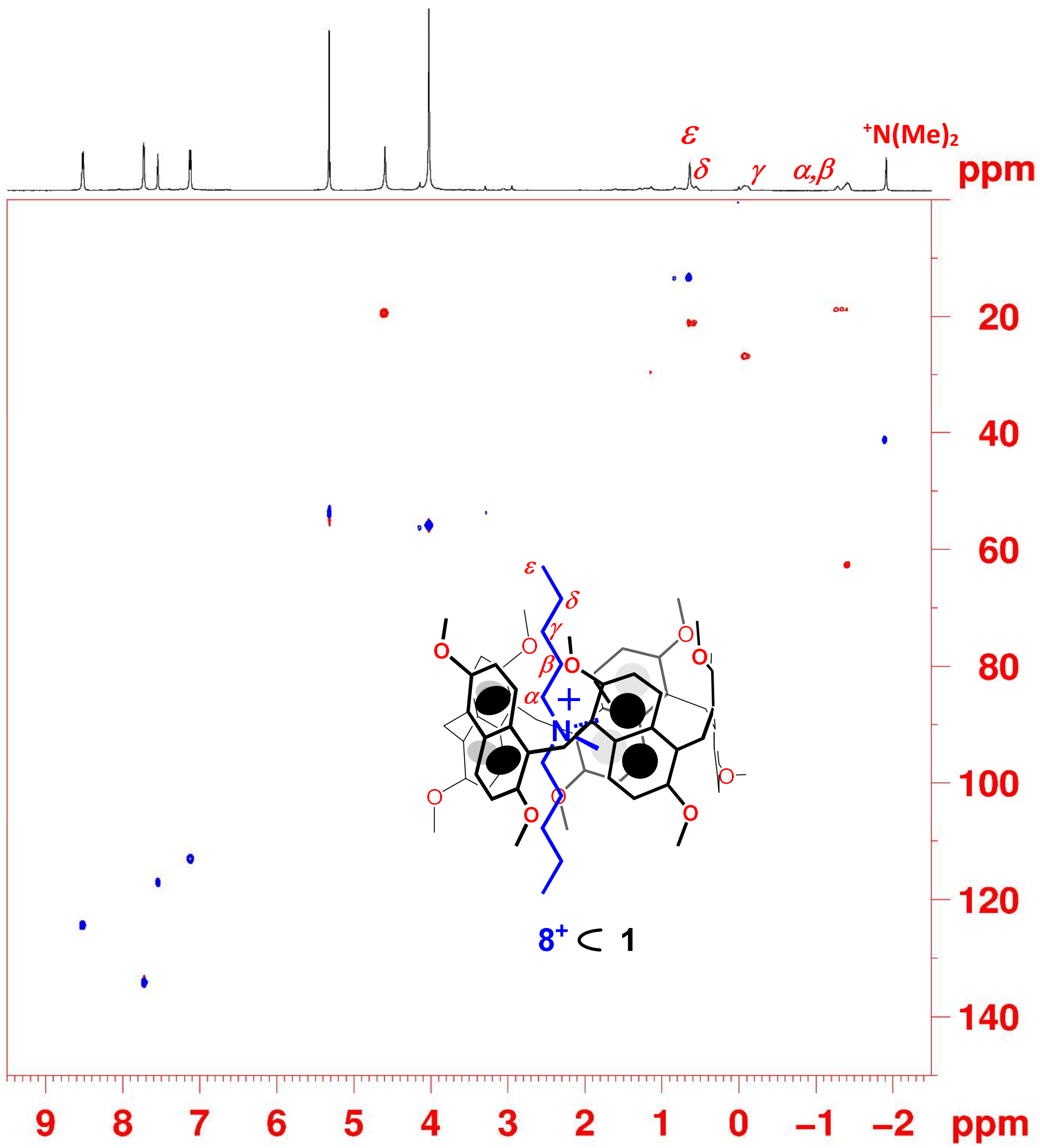

Figure S35: 2D-HSQC spectrum of **8**$^+$⊂ **1** (CD_2Cl_2, 600 MHz, 253 K).

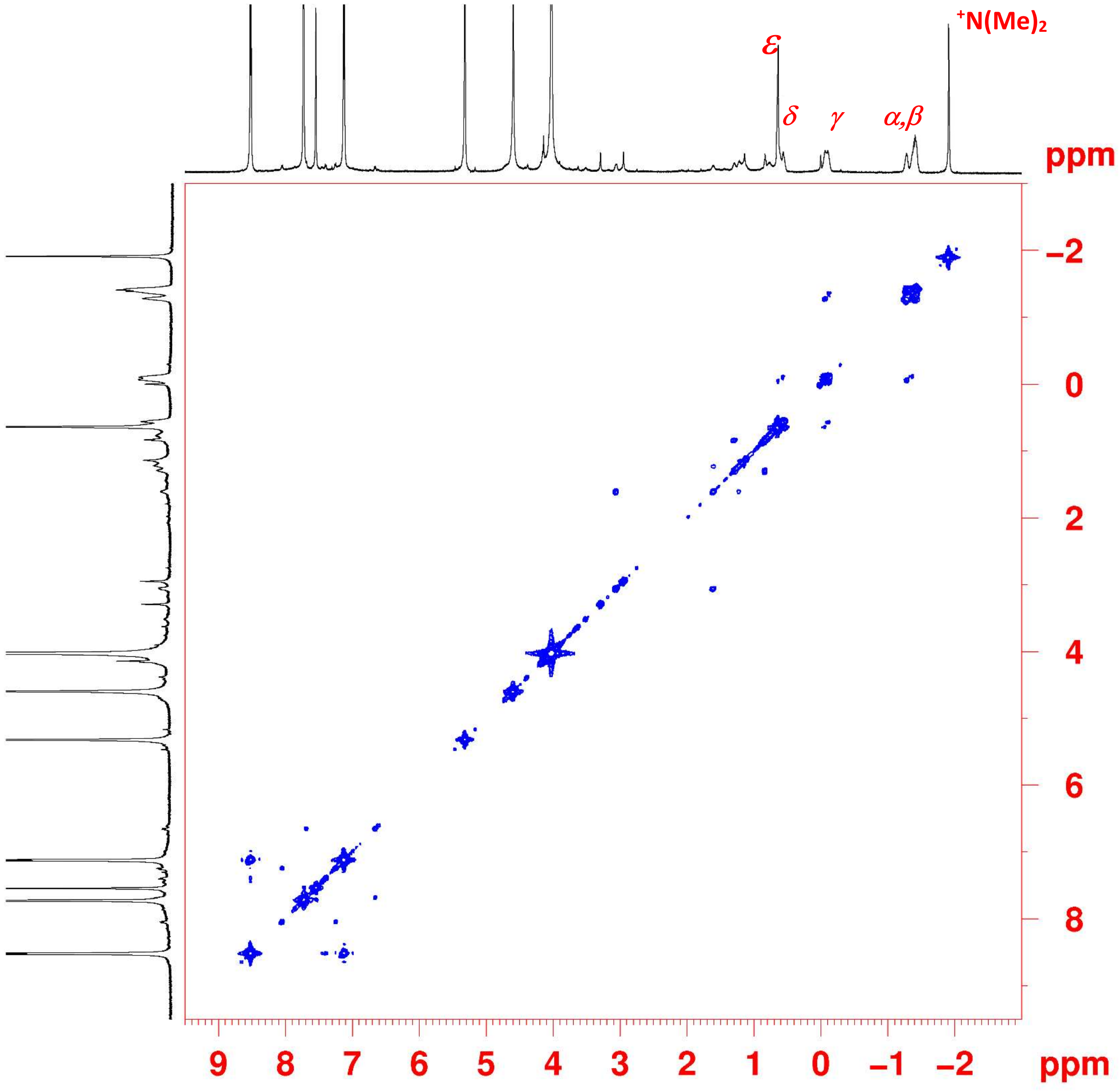

Figure S36: 2D-DQF COSY spectrum of **8**$^{+}$⊂ **1** (CD_2Cl_2, 600 MHz, 183 K).

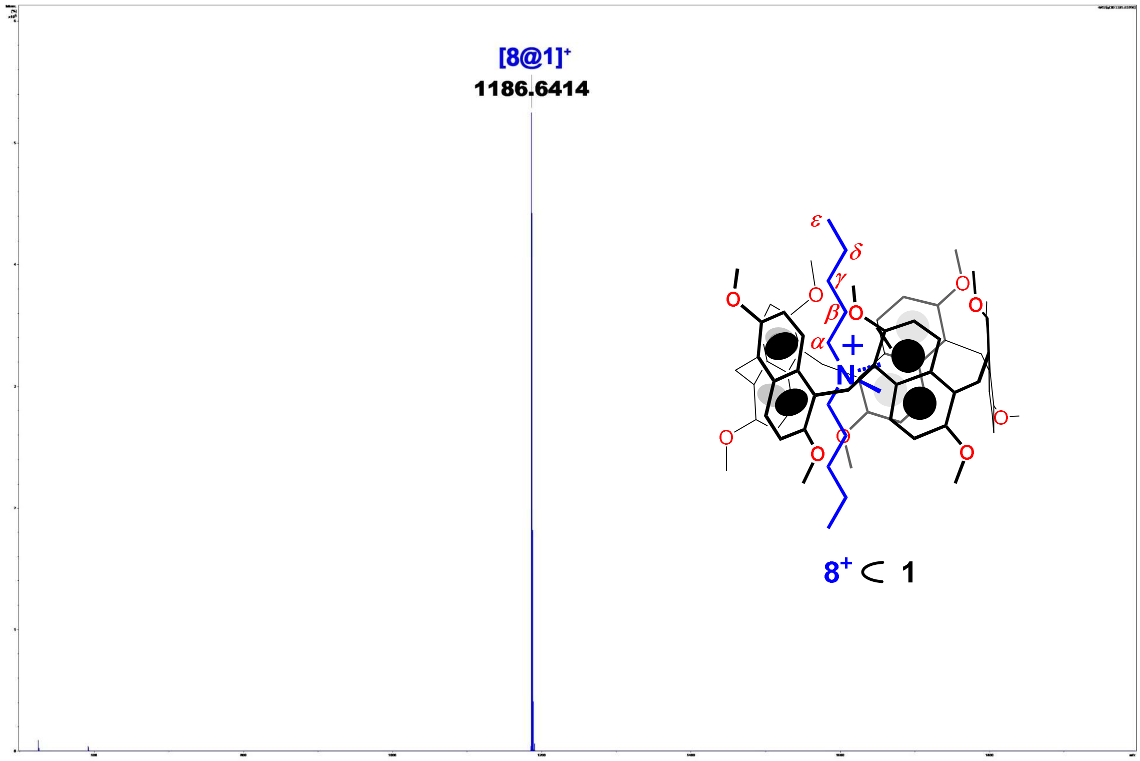

Figure S37: Significant portion of the HR ESI FT-ICR mass spectrum of **[8⊂1]** $^{+}$.

Copies of NMR and HR Mass Spectra of $12^{+}\subset 1$

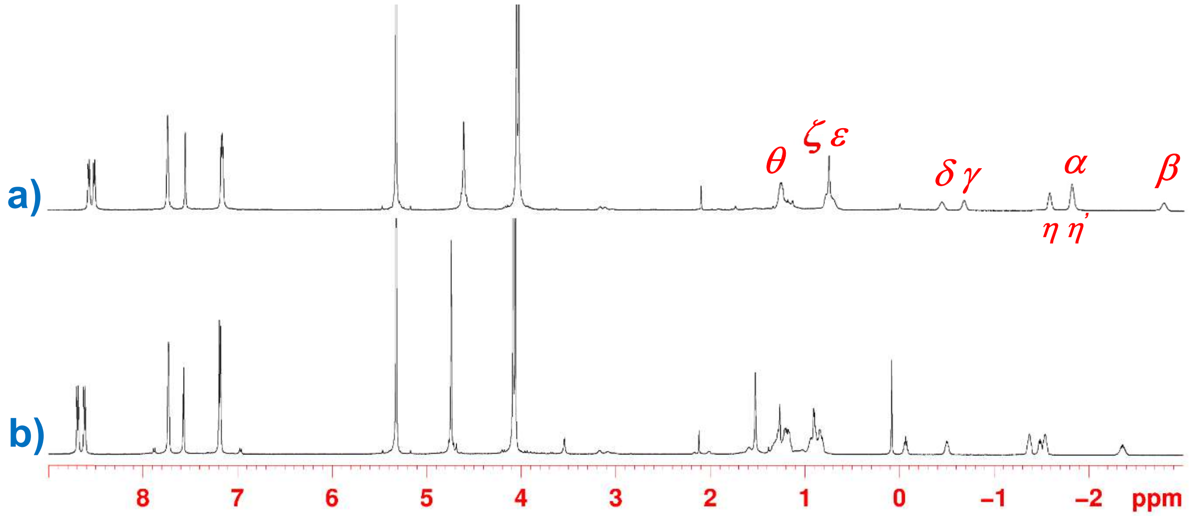

Figure S38: ^{1}H NMR spectra of an equimolar solution of **1** and **$12^{+}\cdot$ TFPB^{-}** (a) at 183 K and (b) at 298 K (600 MHz, CD_2Cl_2).

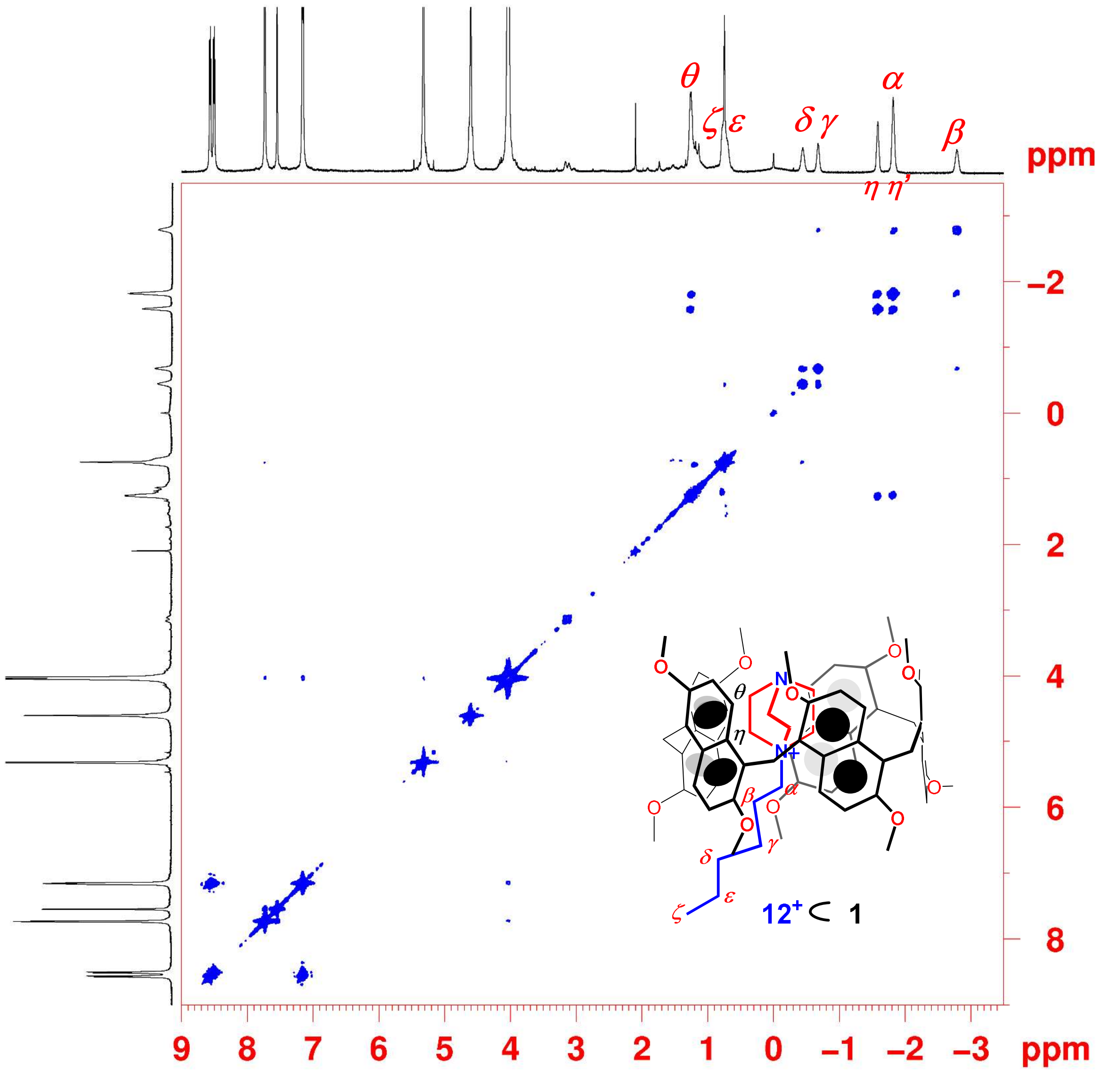

Figure S39: 2D-DQF COSY spectrum of **12^{+}⊂ 1** (CD_2Cl_2, 600 MHz, 183 K).

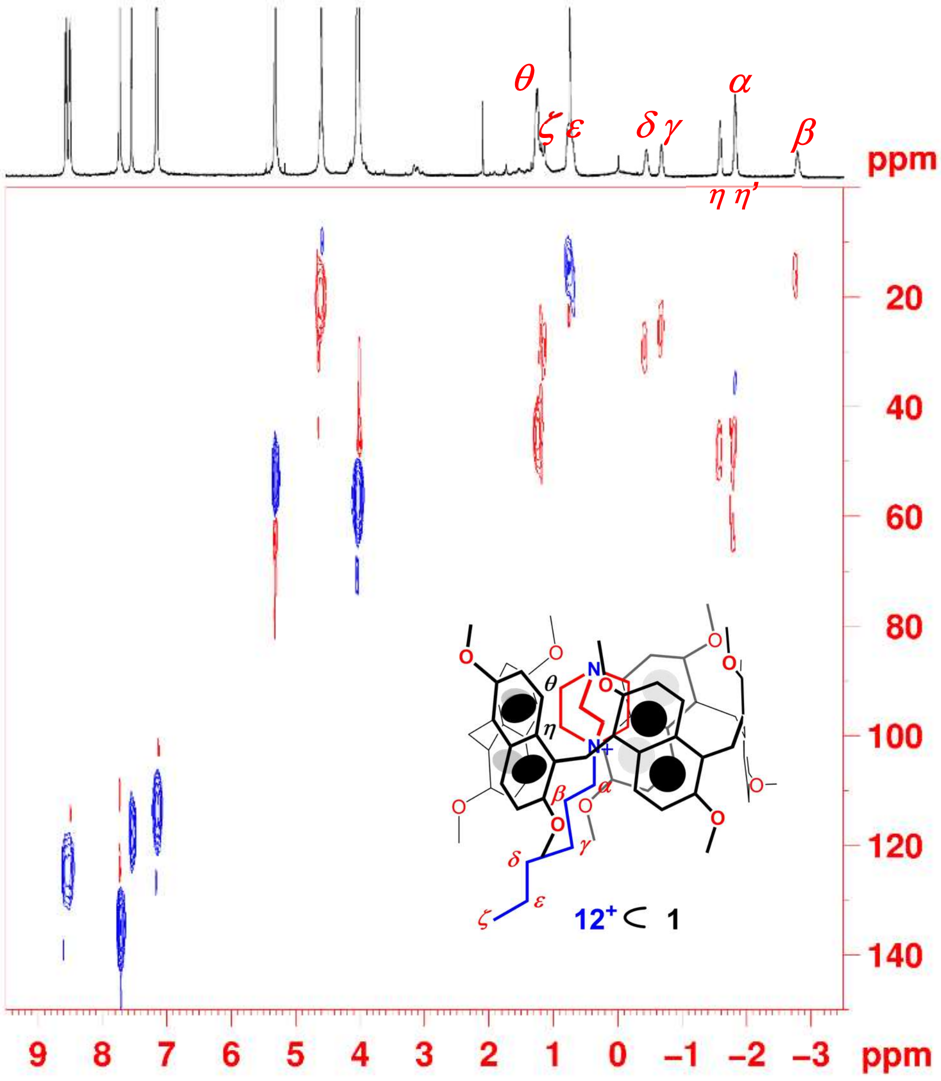

Figure S40: 2D-HSQC spectrum of **12^{+}⊂ 1** (CD_2Cl_2, 600 MHz, 183 K).

Copies of NMR and HR Mass Spectra of $10^{2+} \subset 1$

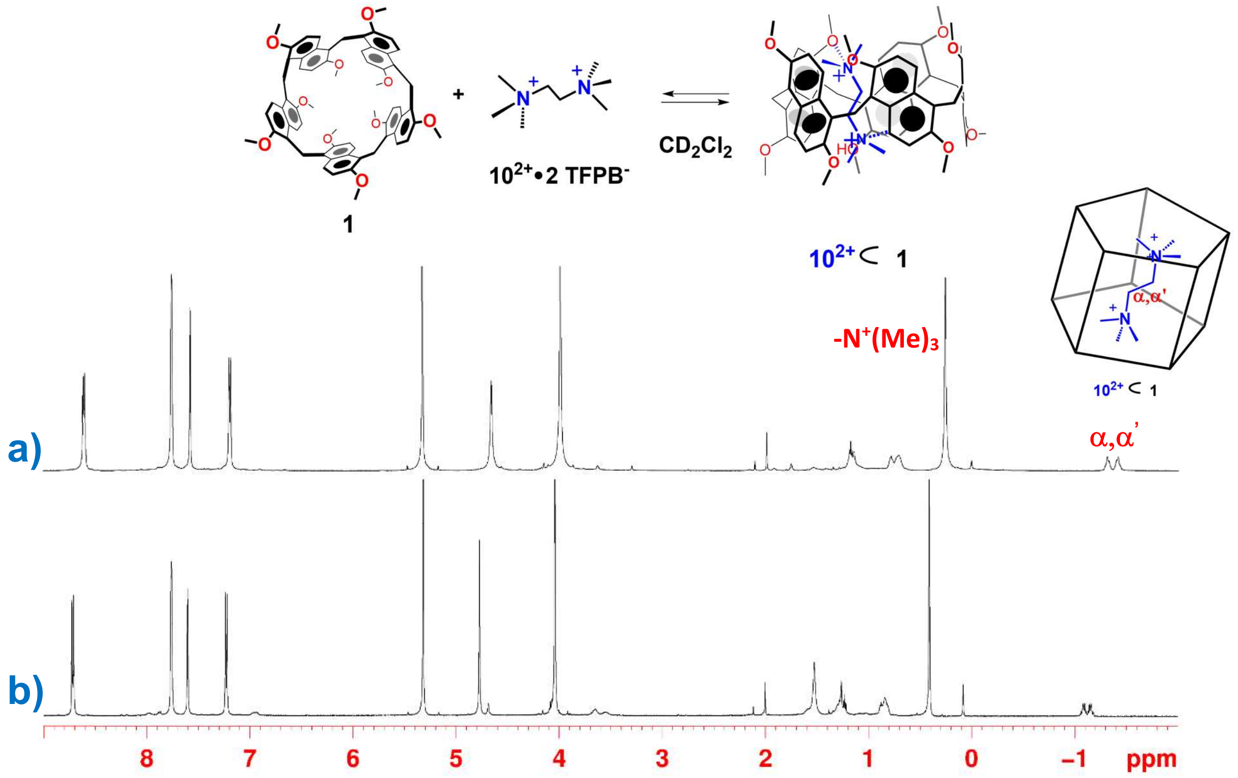

Figure S41: ^{1}H NMR spectra of an equimolar solution of **1** and **$10^{2+} \cdot 2$ TFPB$^-$** (a) at 183 K and (b) at 298 K (600 MHz, CD_2Cl_2).

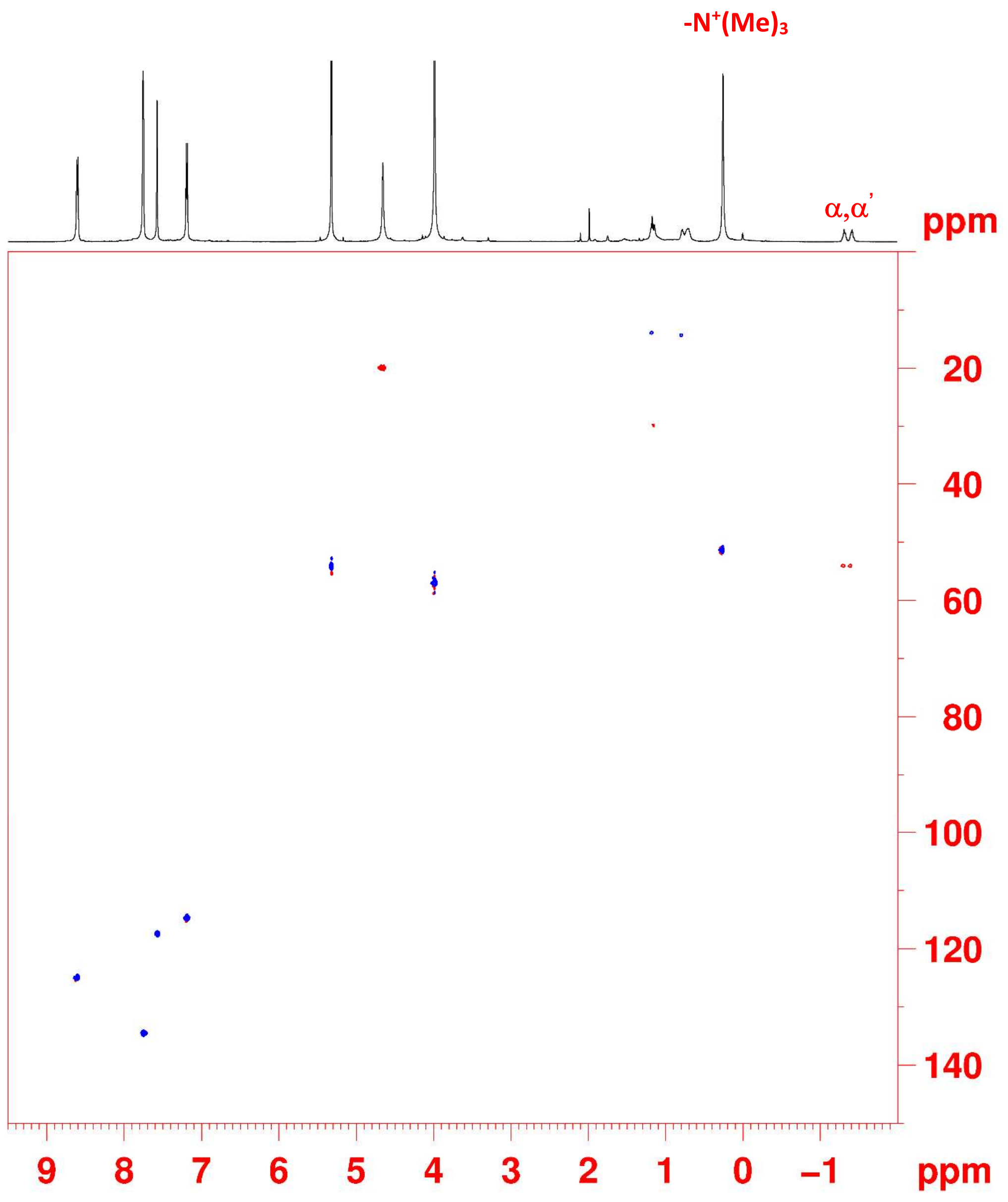

Figure S42: 2D-DQF COSY spectrum of **10**$^{2+}$⊂ **1** (CD_2Cl_2, 600 MHz, 183 K).

Copies of NMR and HR Mass Spectra of $9^{2+} \subset 1$

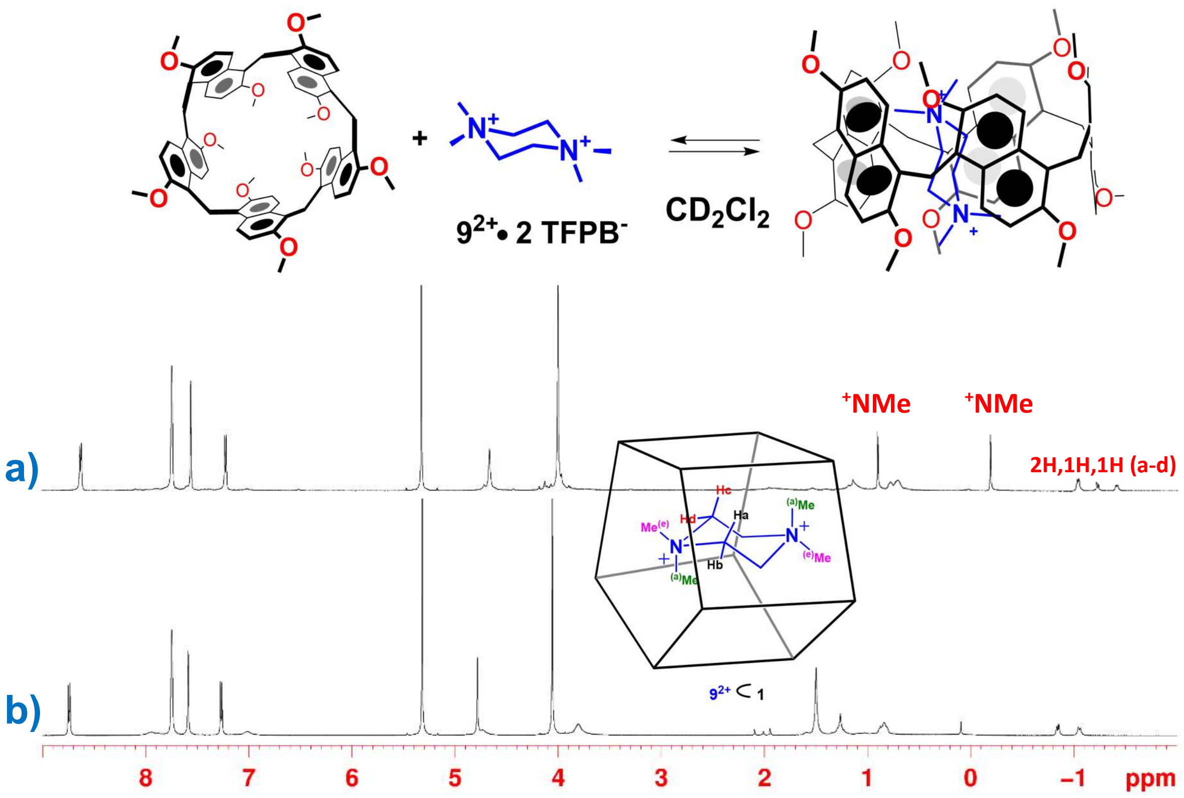

Figure S43: ^{1}H NMR spectra of an equimolar solution of **1** and **9^{2+} · 2 TFPB$^-$** (a) at 183 K and (b) at 298 K (600 MHz, CD_2Cl_2).

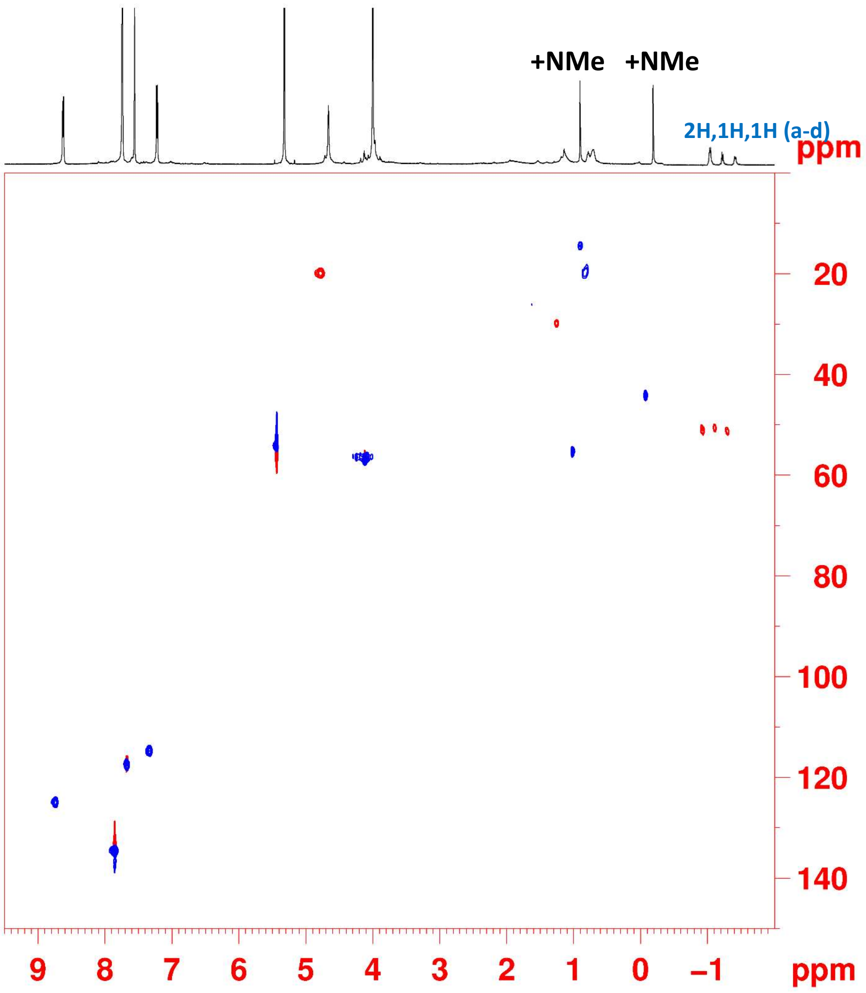

Figure S44: 2D-HSQC spectrum of $\mathbf{9}^{2+}\subset\mathbf{1}$ (CD_2Cl_2, 600 MHz, 183 K).

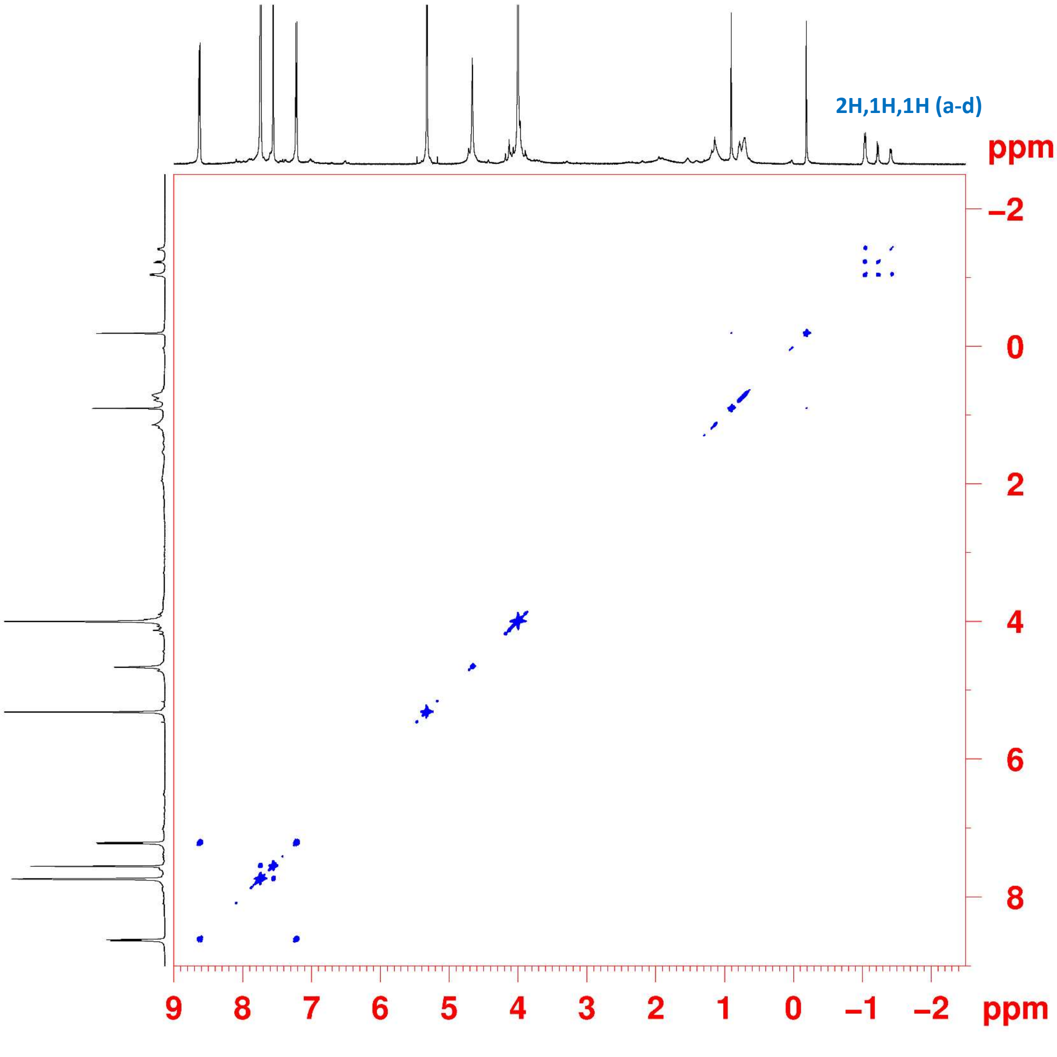

Figure S45: 2D-DQF COSY spectrum of $\mathbf{9}^{2+}\subset\mathbf{1}$ (CD_2Cl_2, 600 MHz, 183 K).

Copies of NMR and HR Mass Spectra of $5^{+} \subset 2$

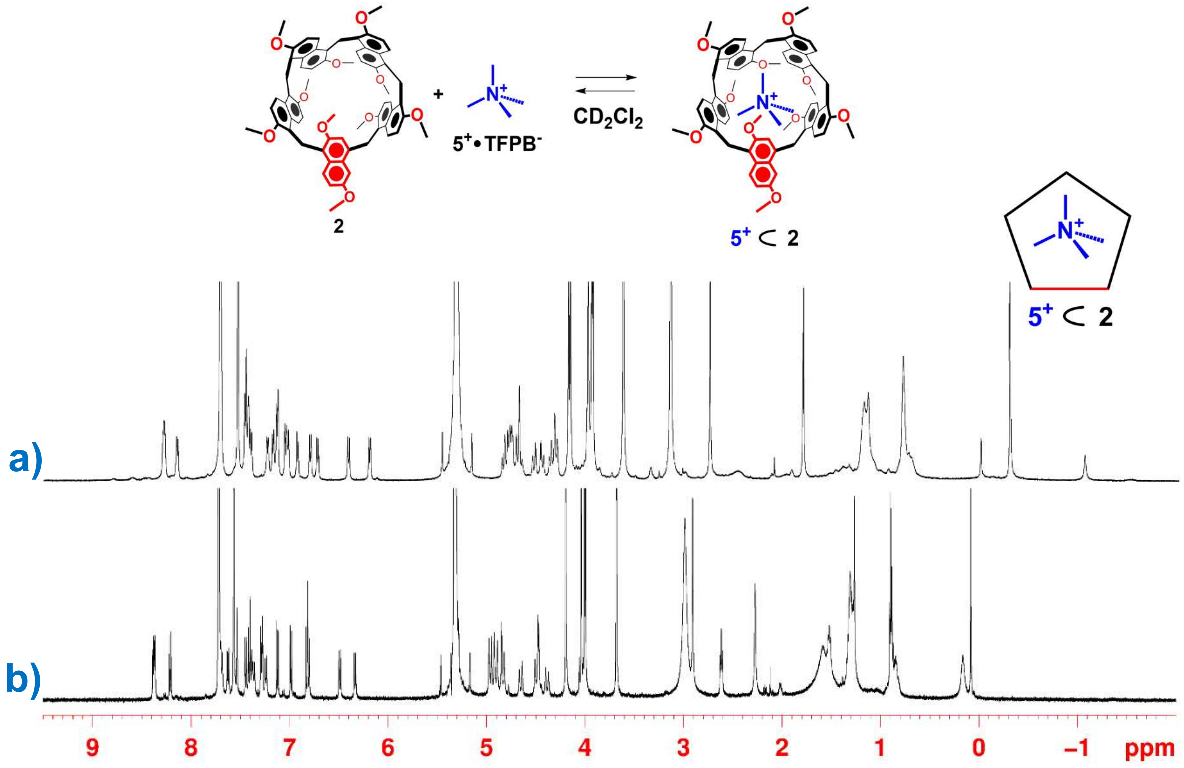

Figure S46: ^{1}H NMR spectra of an equimolar solution of **2** and **5**$^{+}$ (a) at 183 K and (b) at 298 K (600 MHz, CD_2Cl_2).

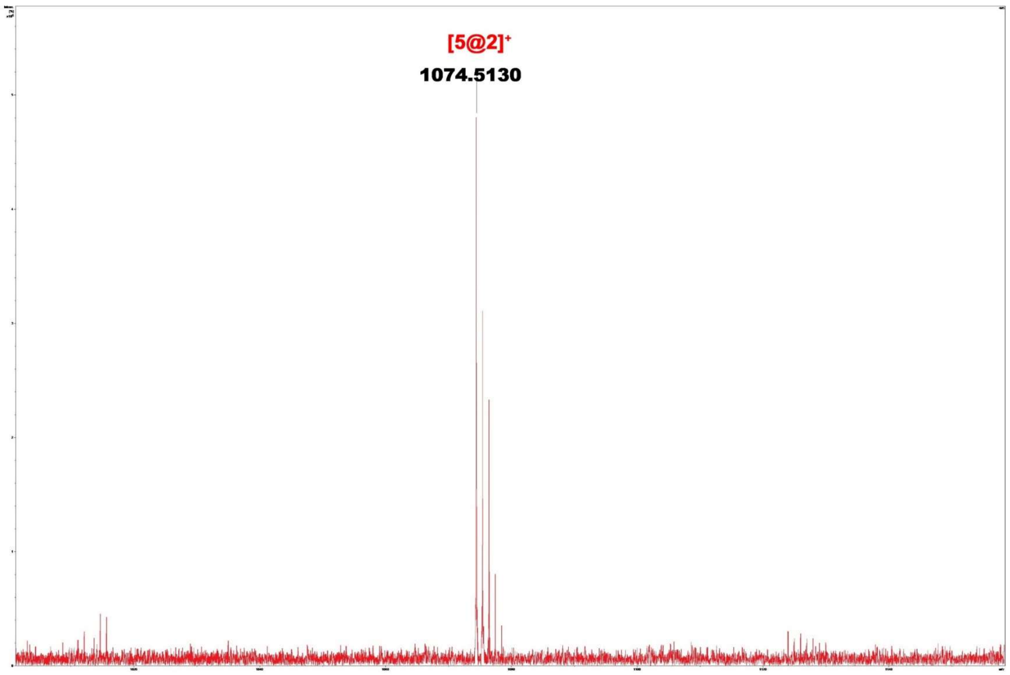

Figure S47: Significant portion of the HR ESI FT-ICR mass spectrum of **[5⊂ 2]**$^{+}$.

Copies of NMR and HR Mass Spectra of $7^+ \subset 2$

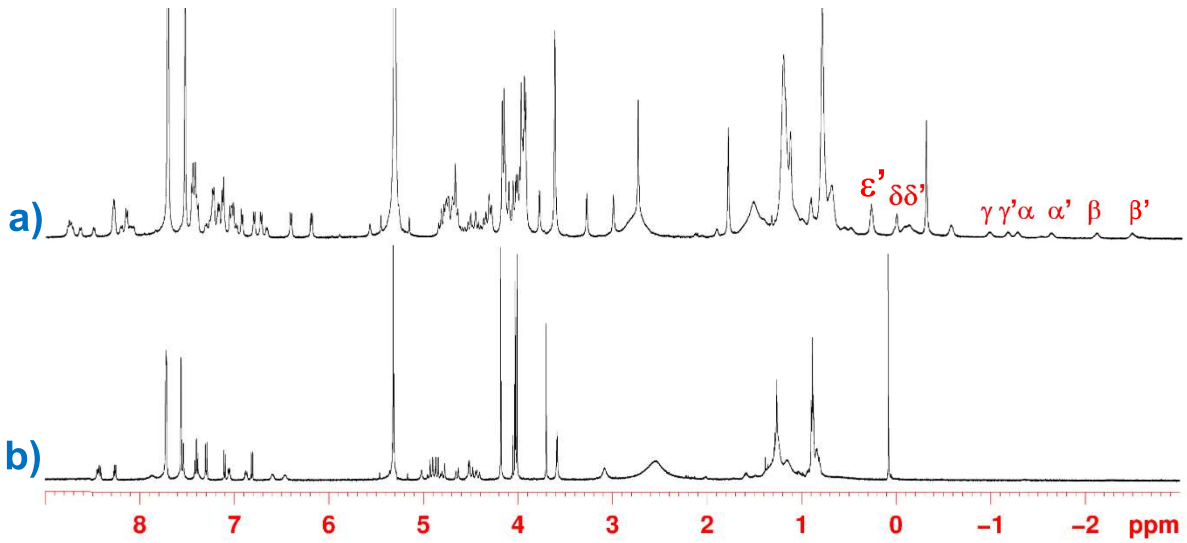

Figure S48: ^{1}H NMR spectra spectrum of an equimolar solution of **2** and **7**$^+$ (a) at 183 K and (b) at 298 K (600 MHz, CD_2Cl_2).

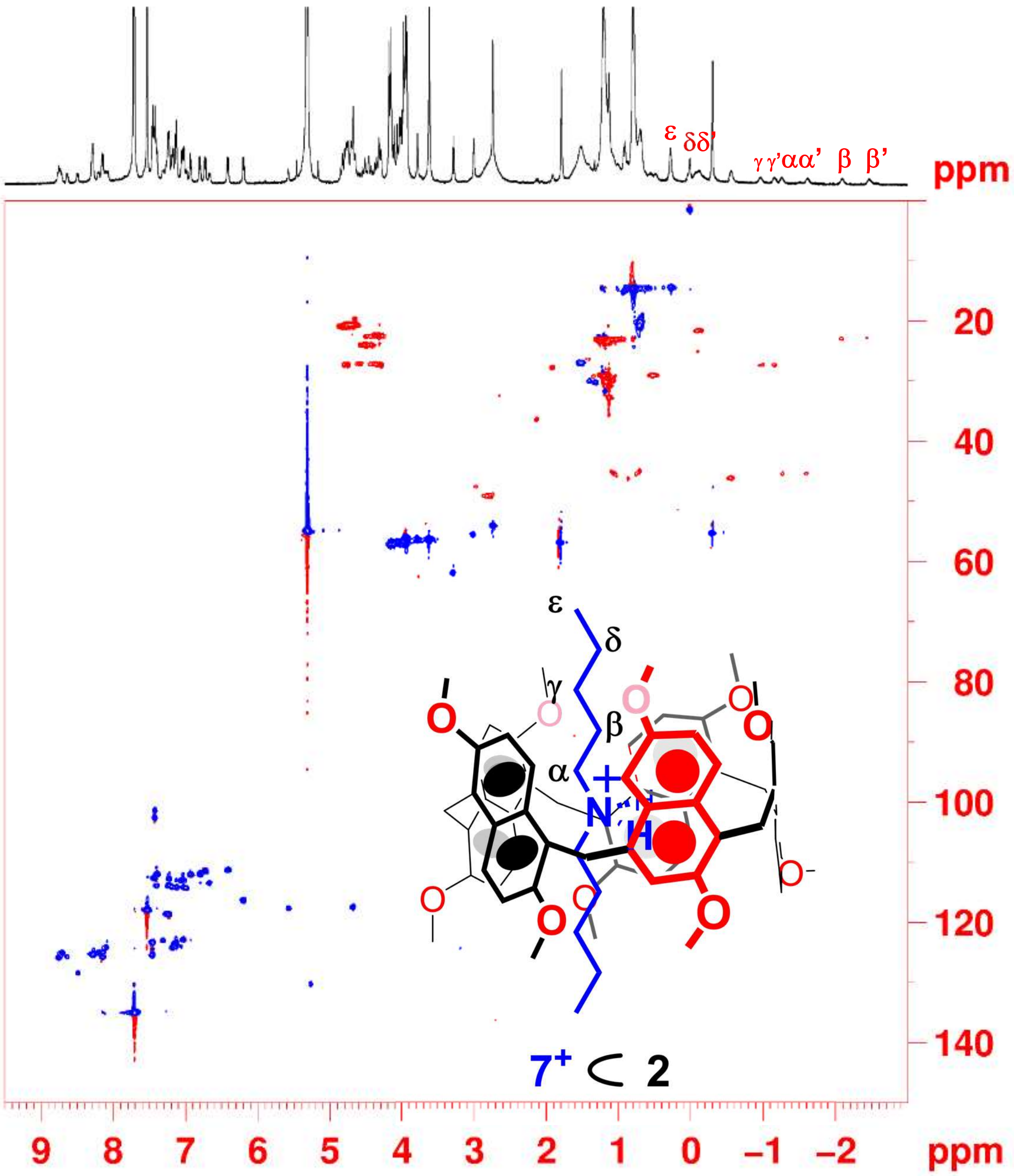

Figure S49: 2D-HSQC spectrum of $7^{+}\subset$ **2** (CD_2Cl_2, 600 MHz, 183 K).

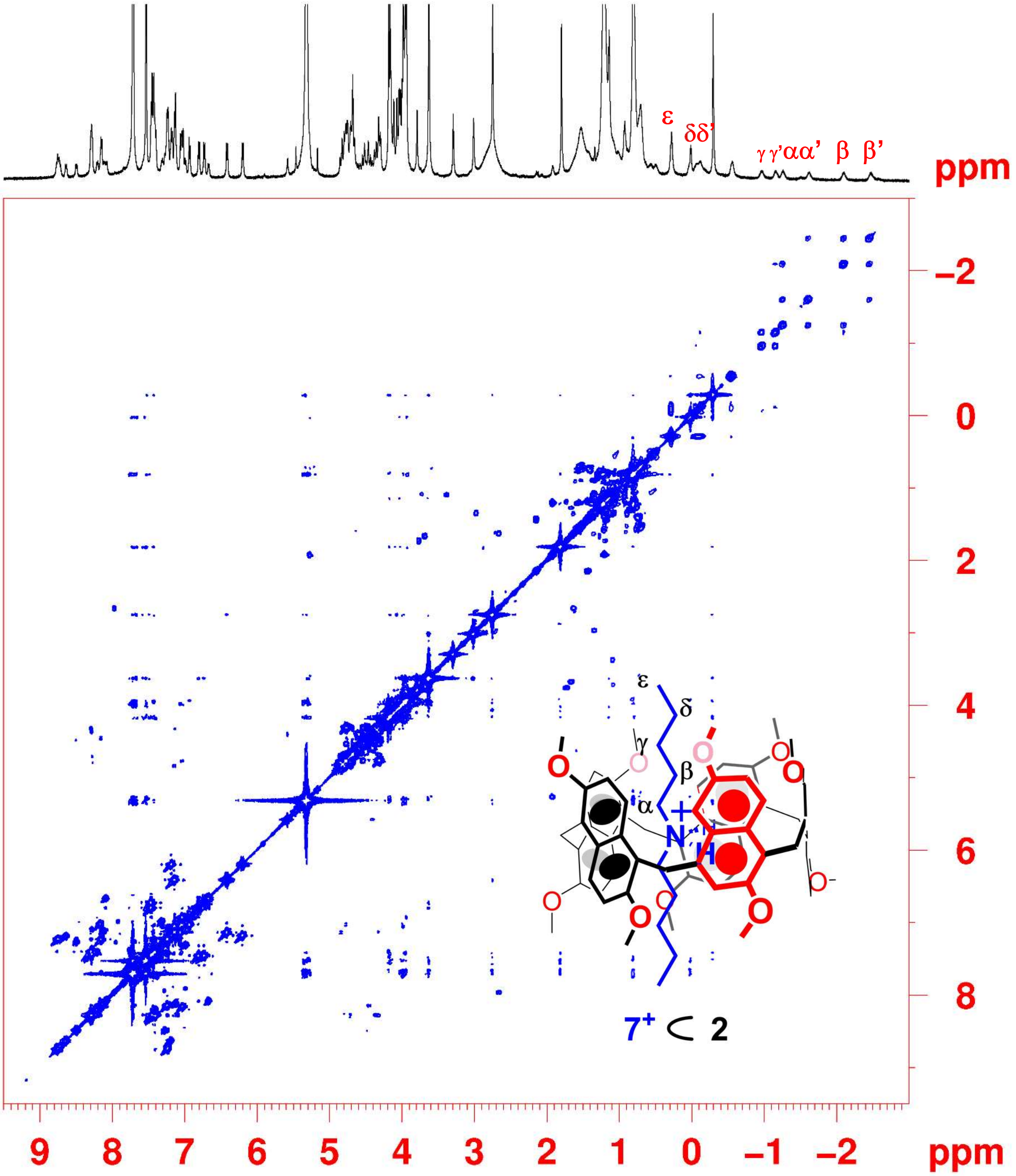

Figure S50: 2D-DQF COSY spectrum of $7^+ \subset$ **2** (CD_2Cl_2, 600 MHz, 183 K).

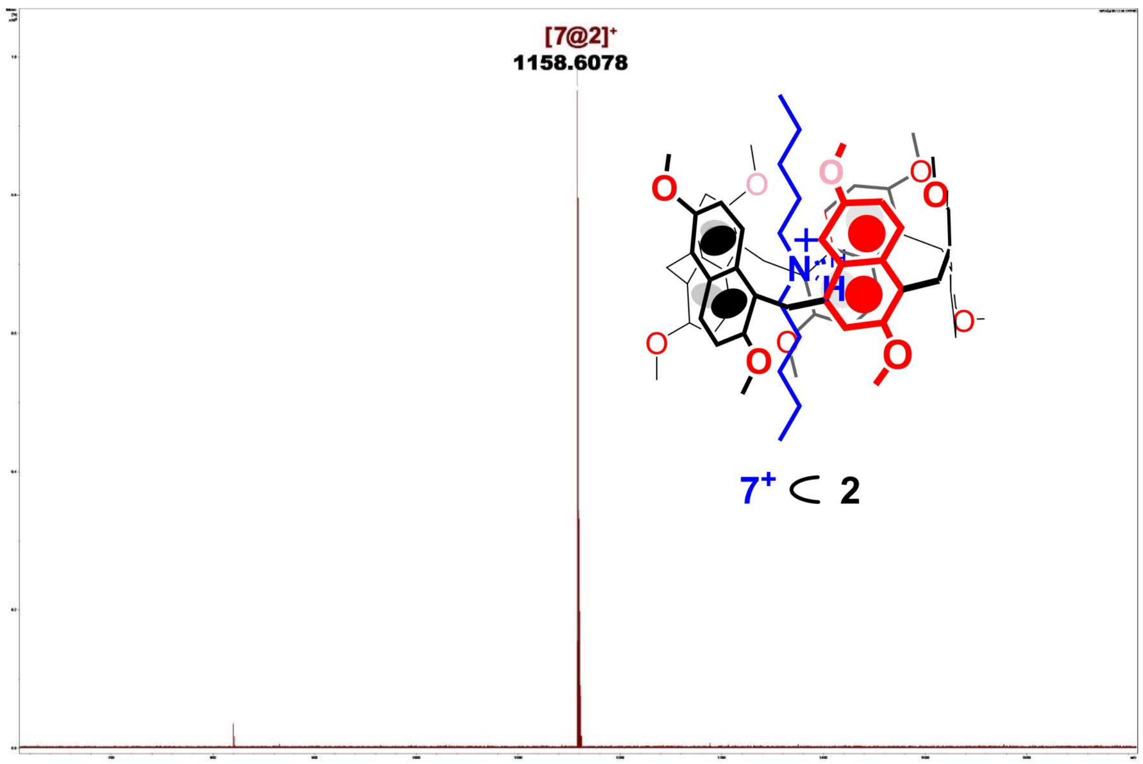

Figure S51: Significant portion of the HR ESI FT-ICR mass spectrum of **[7⊂ 2]** $^{+}$.

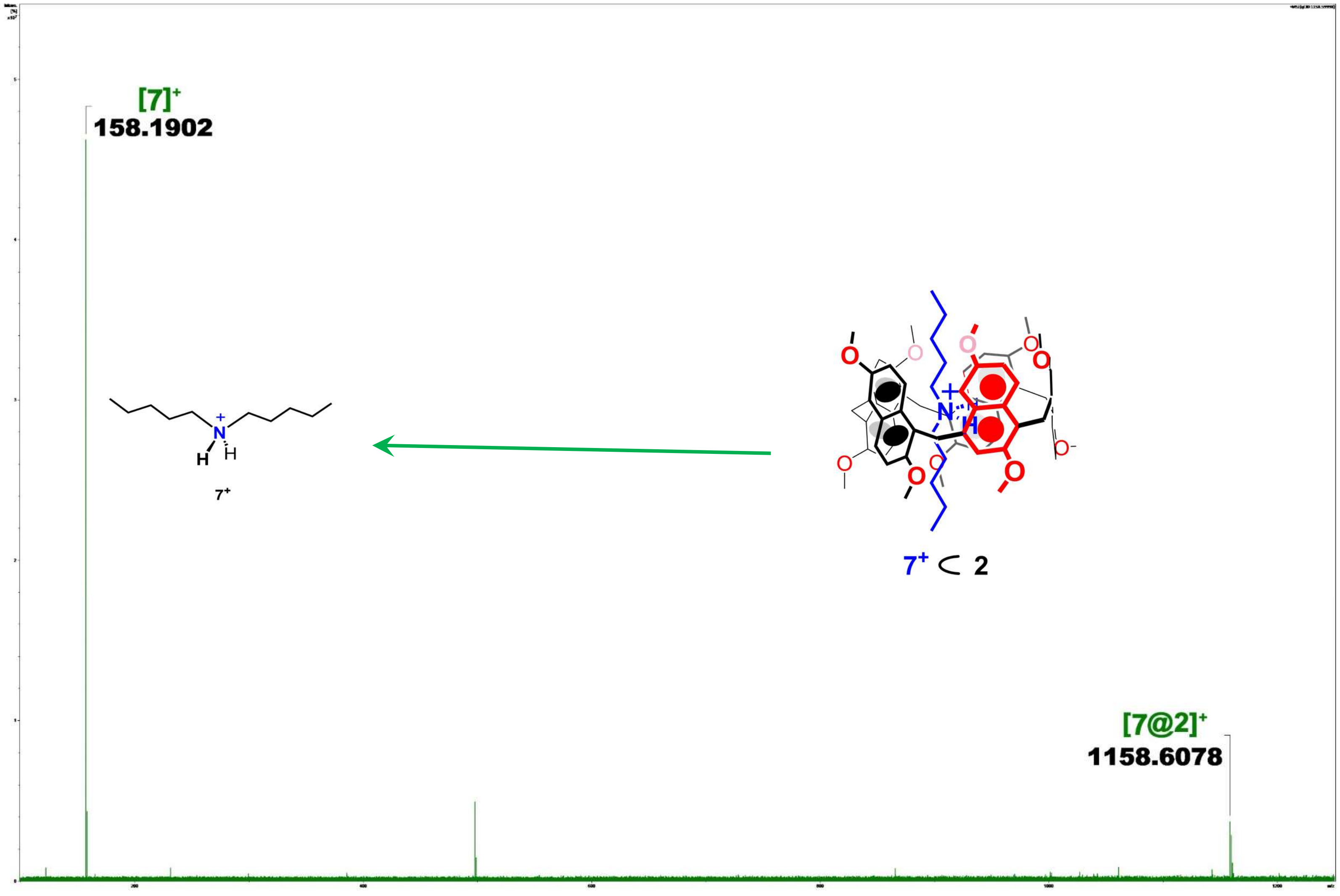

Figure S52: HR ESI-FT-ICR-CID mass spectrum of **[7⊂ 2]** $^{+}$

Copies of NMR and HR Mass Spectra of $6^{+}\subset 3$

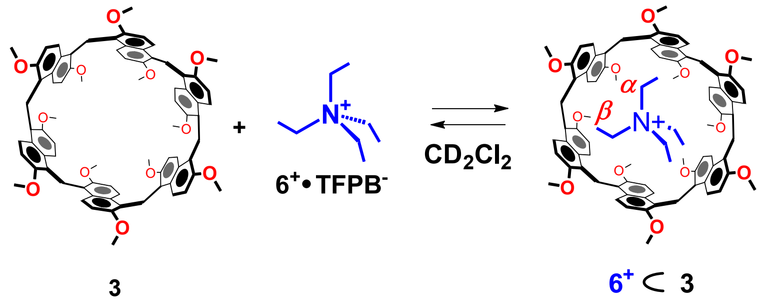

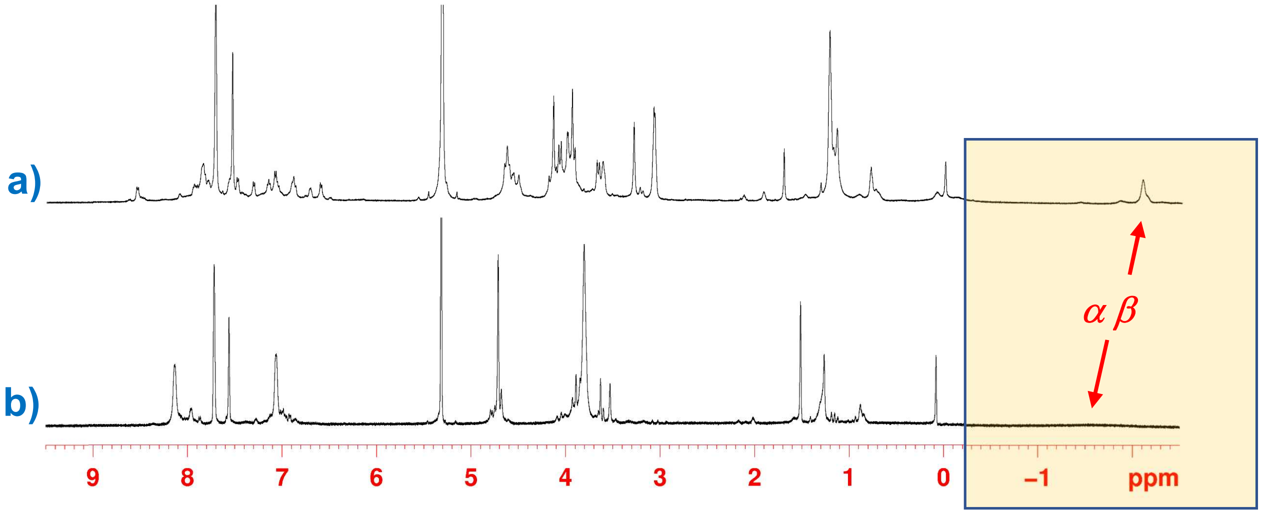

Figure S53: ^{1}H NMR spectra of an equimolar solution of **3** and **6^{+}· TFPB^{-}** (a) at 183 K and (b) at 298 K (600 MHz, CD_2Cl_2).

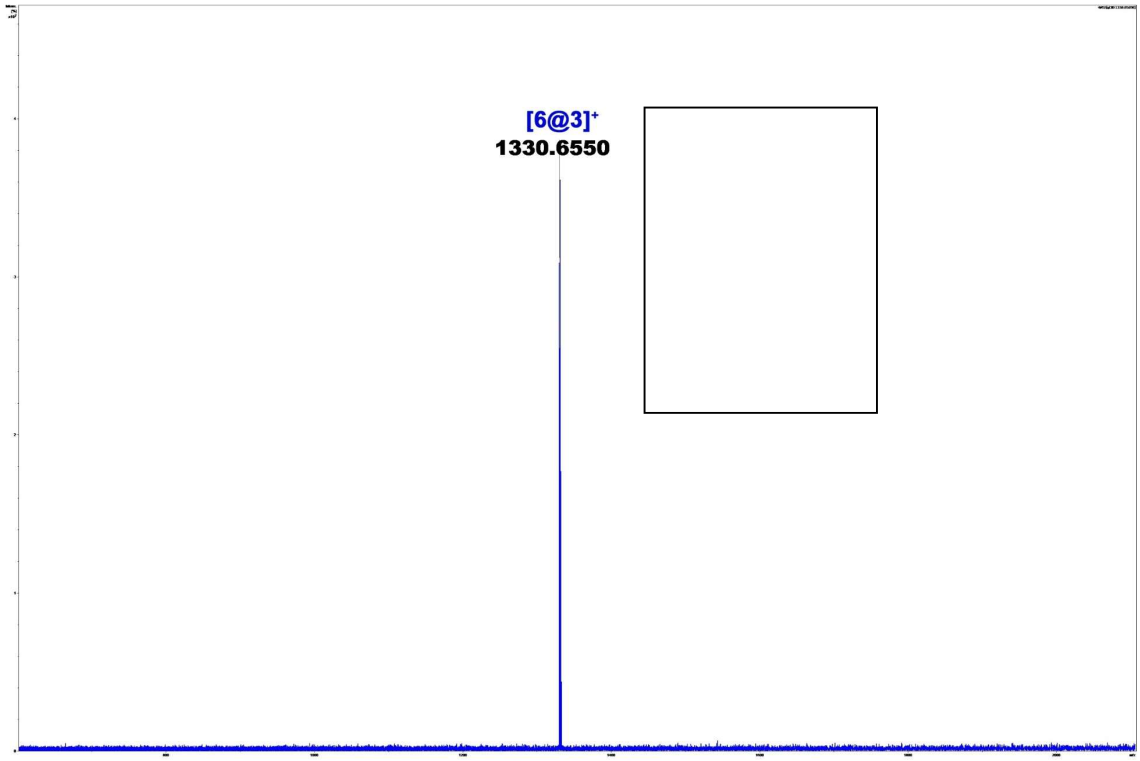

Figure S54: Significant portion of the HR ESI FT-ICR mass spectrum of **$[6\subset 3]^{+}$**.

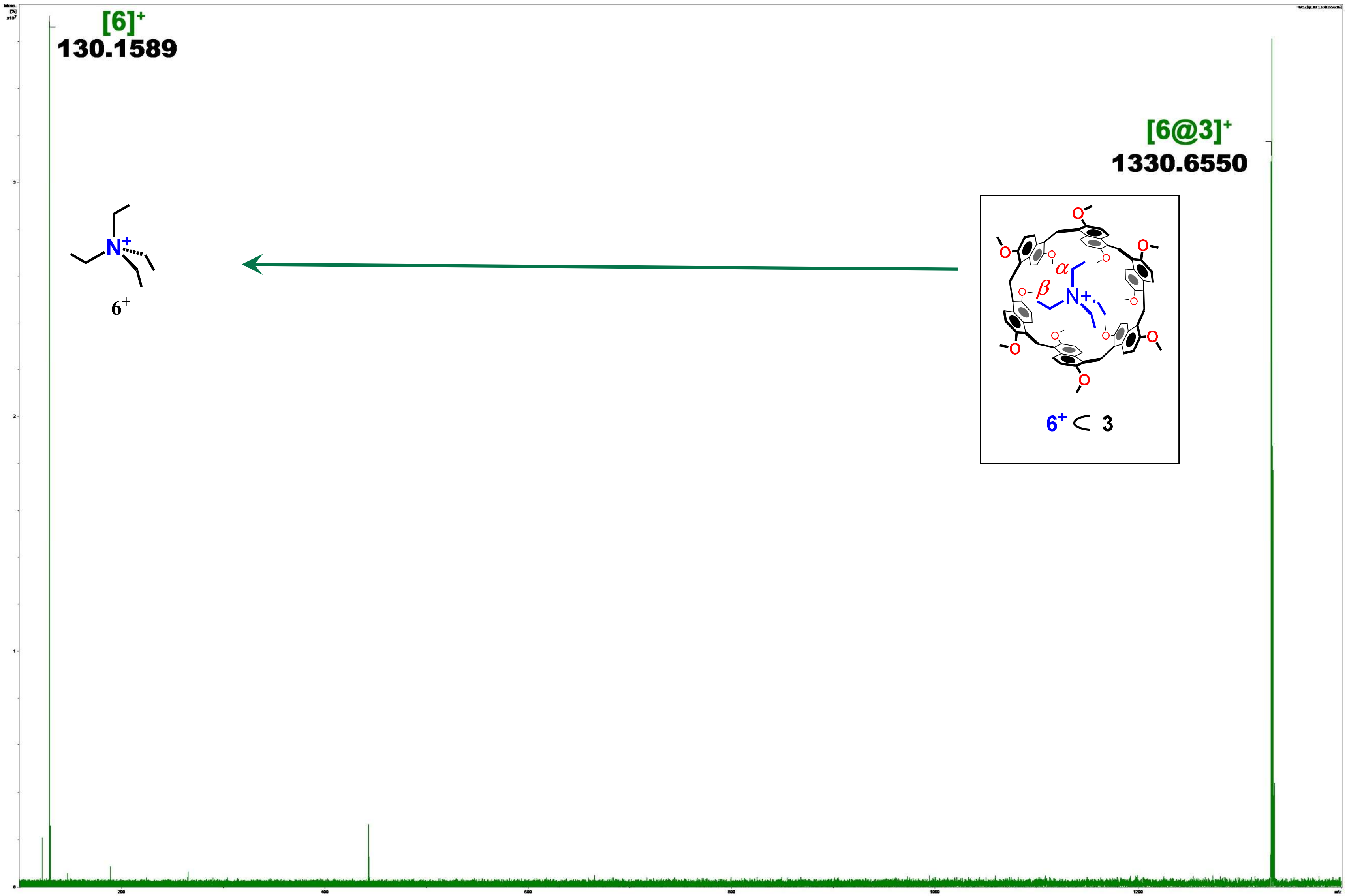

Figure S55: HR ESI-FT-ICR-CID mass spectrum of **[6⊂ 3]**$^+$.

Copies of HR Mass Spectra of prismarenes complexes

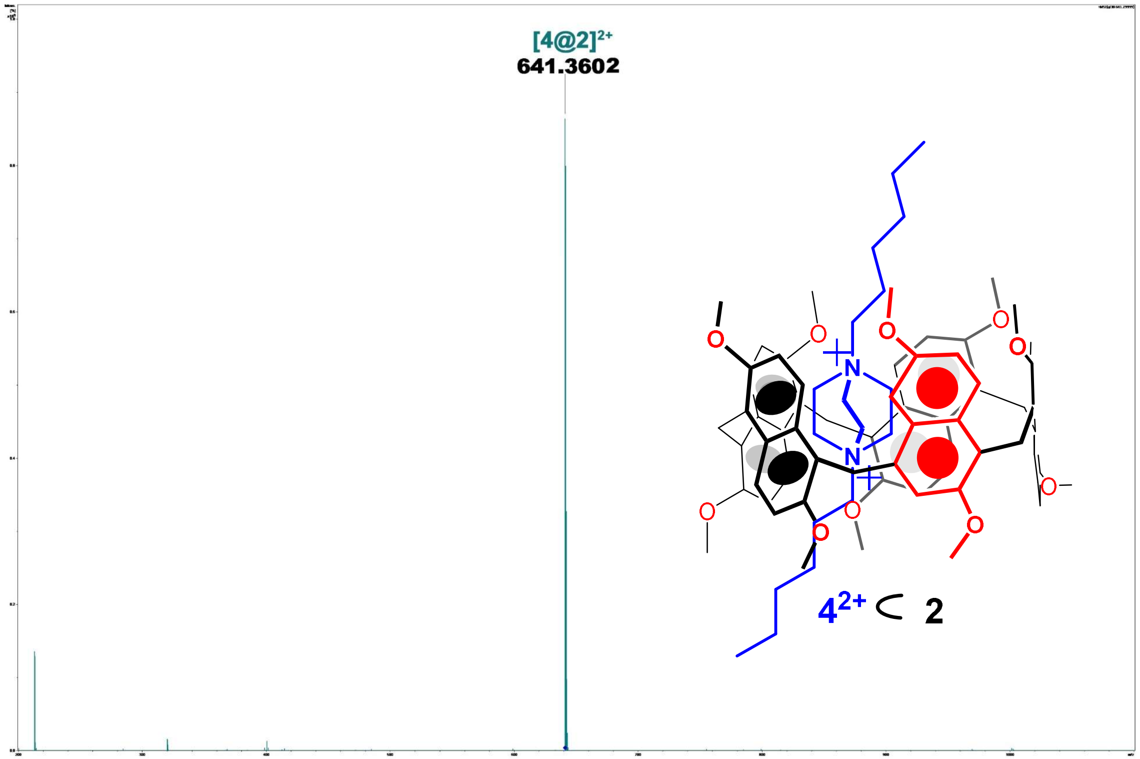

Figure S56: Significant portion of the HR ESI FT-ICR mass spectrum of **[4⊂ 2]** $^{2+}$.

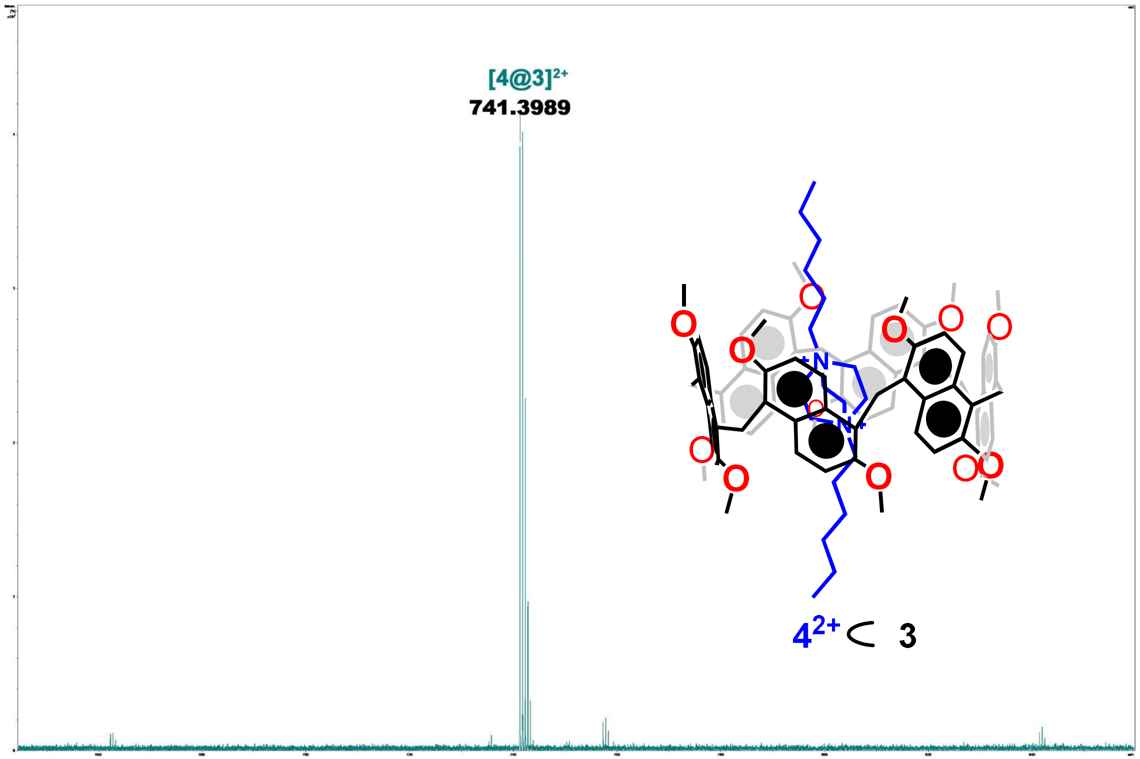

Figure S57: Significant portion of the HR ESI FT-ICR mass spectrum of **[4⊂3]$^{2+}$**.

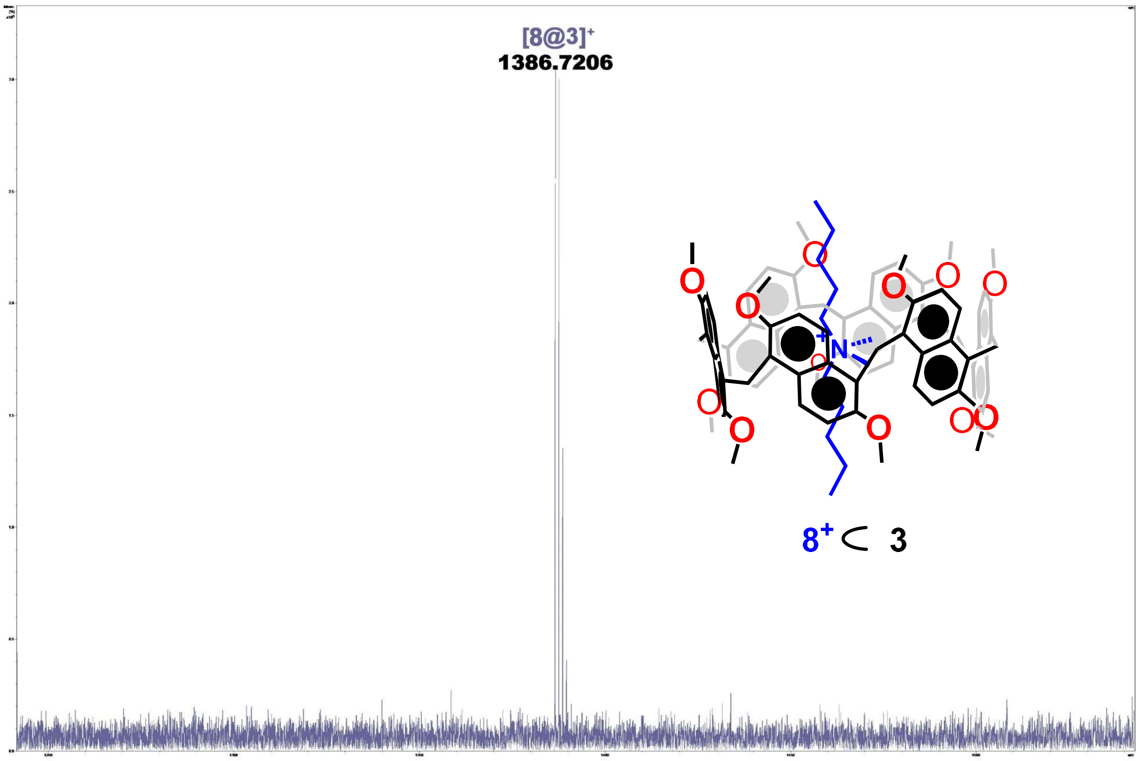

Figure S58: Significant portion of the HR ESI FT-ICR mass spectrum of **[8⊂3]**$^{+}$.

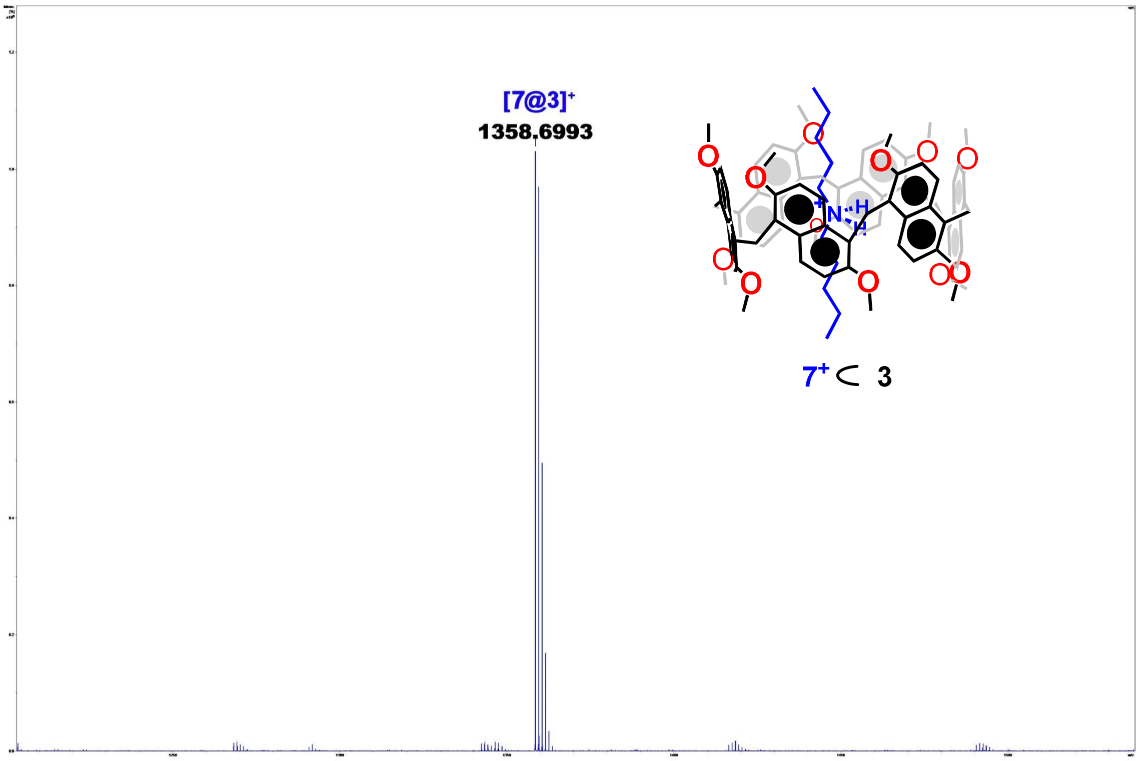

Figure S59: Significant portion of the HR ESI FT-ICR mass spectrum of **[7⊂3]**$^+$.

^{1}H NMR determination of K_{ass} values.[3]

The association constant values of complexes were calculated by means of three methods:

a) Integration of free and complexed ^{1}H NMR signals of host or guest. In this case, an equimolar solution (2.85 mM) of hosts and guests was solubilized in CD_2Cl_2 and equilibrated in a NMR tube after mixing for 24 h at 40 °C.
b) ^{1}H NMR competition experiments. In this case, was performed an analysis of a 1:1:1 mixture of host, and two guests in an NMR tube using 0.7 mL of CD_2Cl_2 as solvent.
c) Quantitative ^{1}H NMR experiments using TCE as the internal standard[4]. In this case, ^{1}H NMR experiments were carried out on a 1:1 mixture of host and guest in 0.7 mL of CD_2Cl_2 containing 1 μL or 0.5 μL of 1,1,2,2-tetrachloroethane (d= 1.59 g/mL) as internal standard.

Table S2. Association constant (K_{ass}, M^{-1}) values for the formation of the complexes between the ammonium **4**$^{2+}$ - **12**$^{+}$ cations as TFPB$^-$ salts and the prism[n]arenes **1**–**3**. Determined by ^{1}H NMR experiments in CD_2Cl_2 (600 MHz). Errors < 15% calculated as mean values of three measures.

	**1**	**2**	**3**
**4**$^{2+}$	$3.9 \cdot 10^{7}$[d]	470[f]	50[f]
**5**$^{+}$	$6.4 \cdot 10^{4}$[e]	20[f]	-
**6**$^{+}$	90[f]	–	2700[f]
**7**$^{+}$	9600[f]	190[f]	310[f]
**8**$^{+}$	$2.0 \cdot 10^{4}$[f]	–	100[f]
**9**$^{2+}$	$1.8 \cdot 10^{7}$[c]	1400[f]	–
**10**$^{2+}$	$4.7 \cdot 10^{6}$[b]	400[f]	–
**11**$^{+}$	150[f]	100[f]	70[f]
**12**$^{+}$	$4.6 \cdot 10^{4}$[a]	50[f]	-

[a]Calculated by quantitative ^{1}H NMR spectroscopy at 298 K analysis using TCE as internal standard (Fig S64), the calculated K_{ass} value was confirmed by competition experiment .[b] Calculated by competition experiment at 298 K with **12**$^{+}$ (Fig S65).[c] Calculated by competition experiment at 298 K with **10**$^{2+}$ (Fig S66).[d] Calculated by competition experiment at 298 K with **9**$^{2+}$ (Fig S67).[e] Calculated by quantitative ^{1}H NMR study at 183 K analysis using TCE as internal standard, the calculated K_{ass} value was confirmed by competition experiment. [f]Calculated at 183 K by integration of ^{1}H NMR signals of free and complexed species.

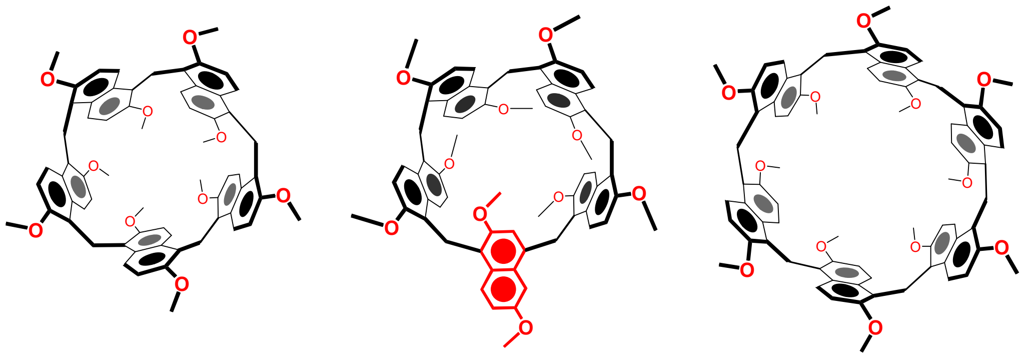

1 2 3

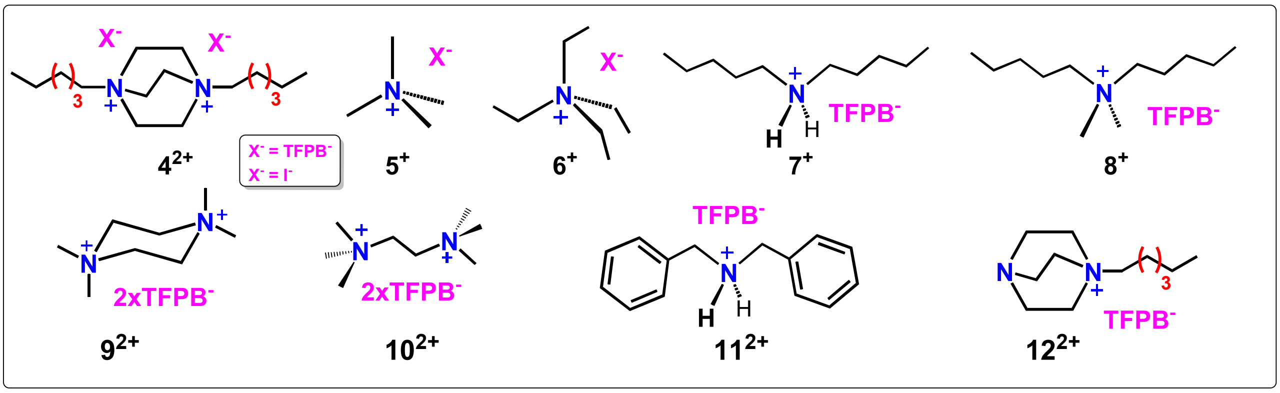

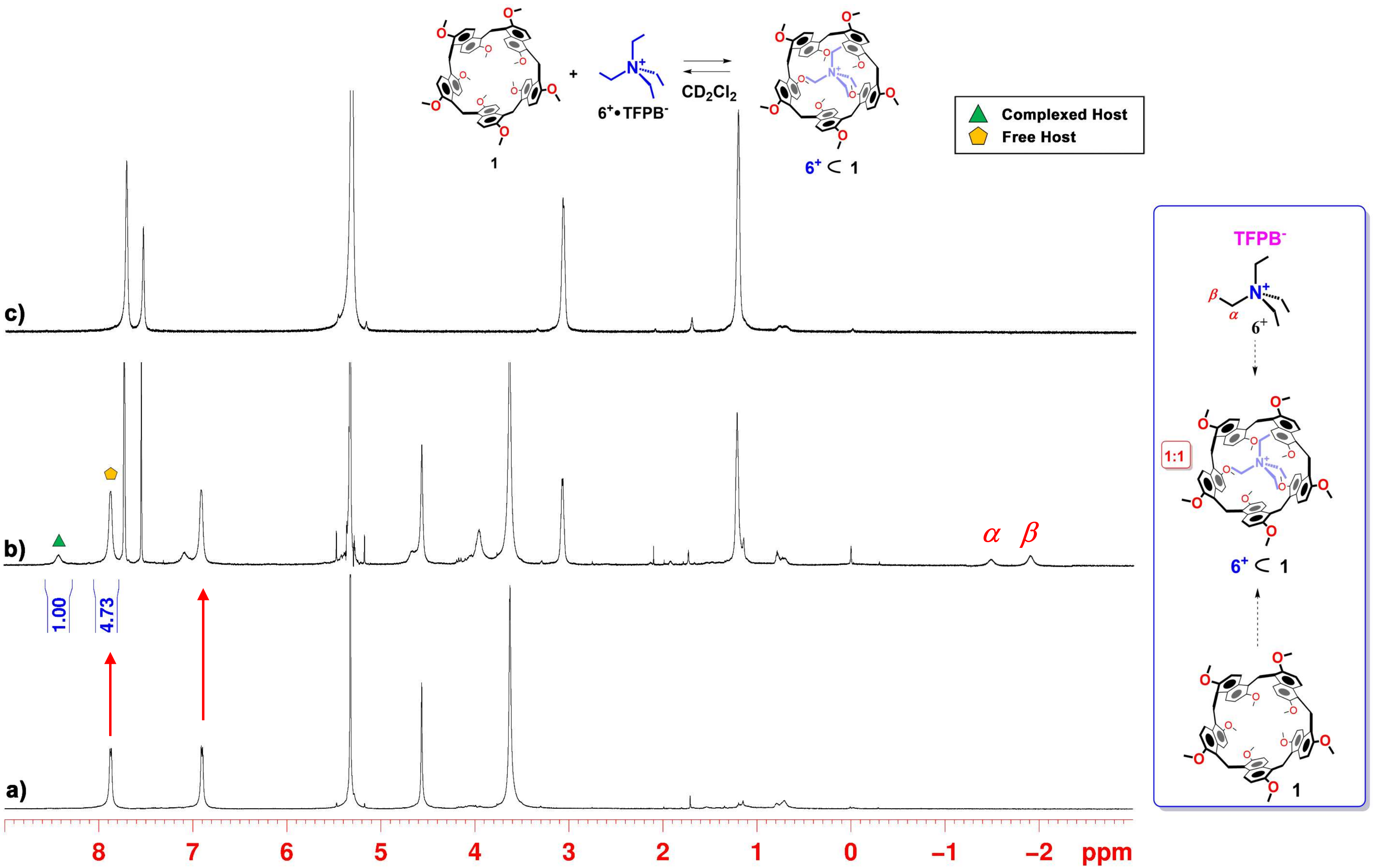

Figure S60: ^{1}H NMR spectra (600 MHz, CD_2Cl_2, 183 K) of: (a) a solution of **1** (b) an equimolar solution (2.85 mM) of **1** and **6^+·TFPB$^-$** in 0.7 mL of CD_2Cl_2 and (c) a solution of **6^+·TFPB$^-$**.

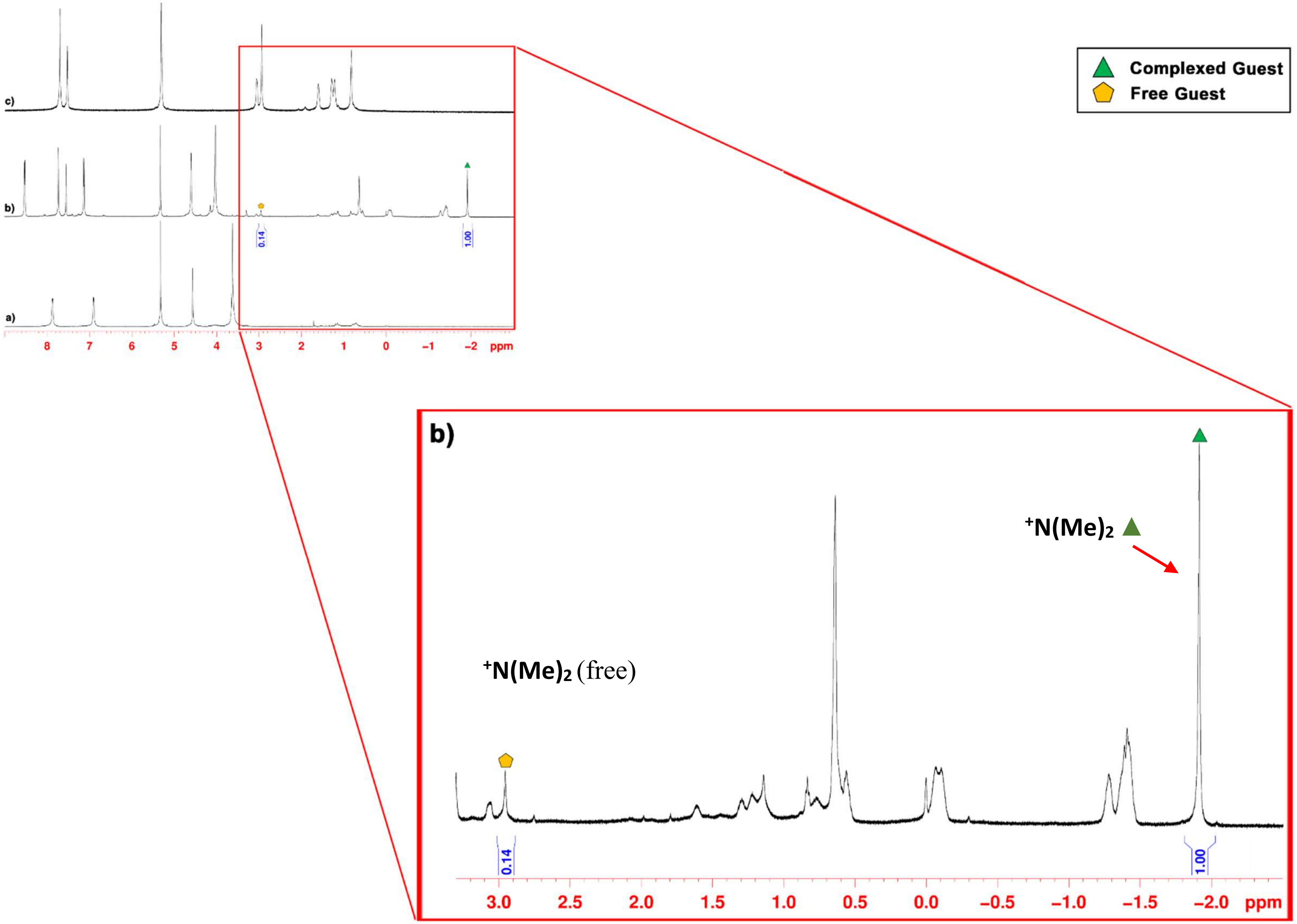

Figure S61: ^1H NMR spectra (600 MHz, CD_2Cl_2, 183 K) of: (a) a solution of **1** (b) an equimolar solution (2.85 mM) of **1** and **8^+·TFPB$^-$** in 0.7 mL of CD_2Cl_2 and (c) a solution of **8^+·TFPB$^-$**.

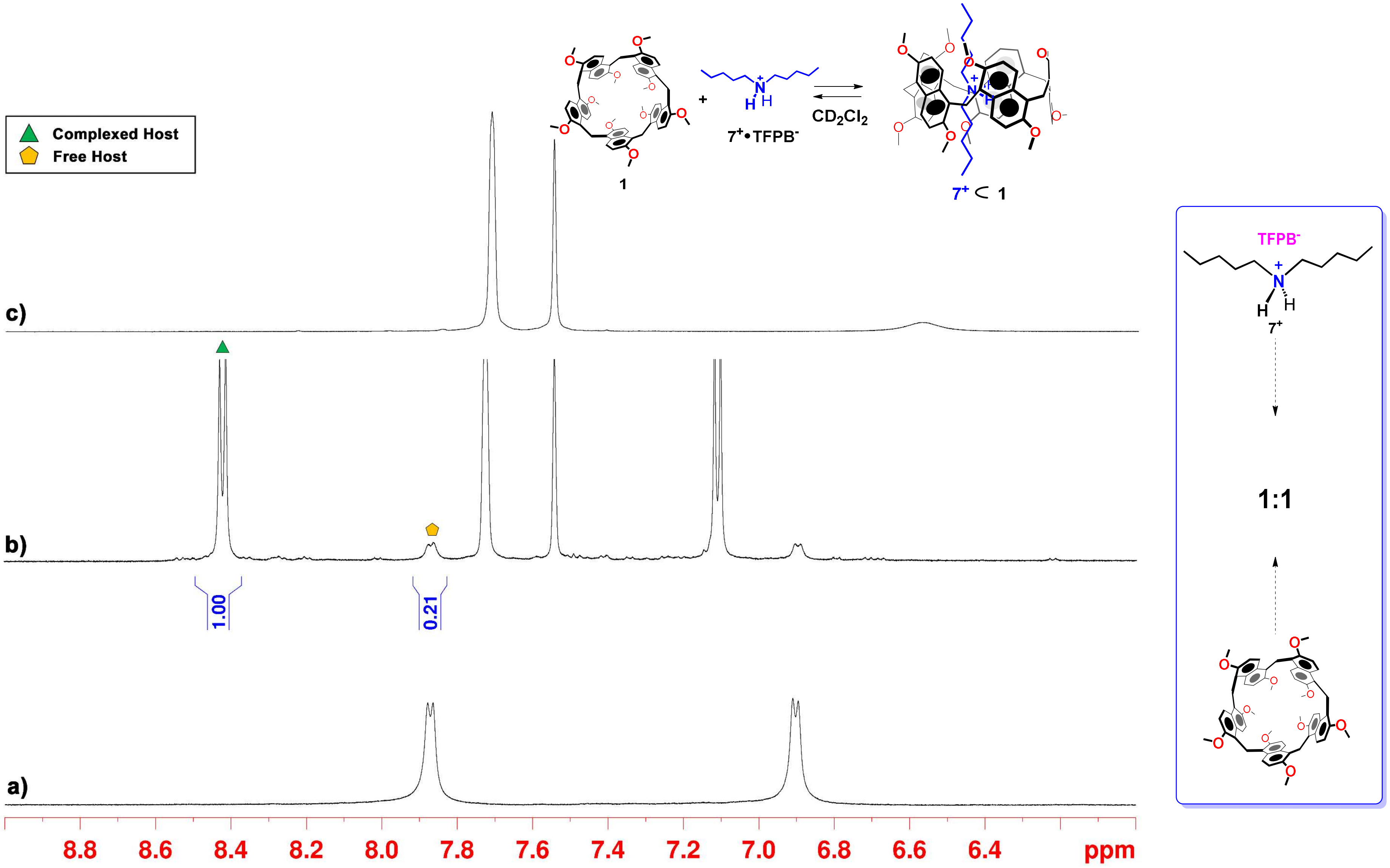

Figure S62: Significant portion of the ^{1}H NMR spectra (600 MHz, CD_2Cl_2, 183 K) of: (a) a solution of **1** (b) an equimolar solution (2.85 mM) of **1** and $\mathbf{7^+}$·**TFPB**$^-$ in 0.7 mL of CD_2Cl_2 and (c) a solution of $\mathbf{7^+}$·**TFPB**$^-$.

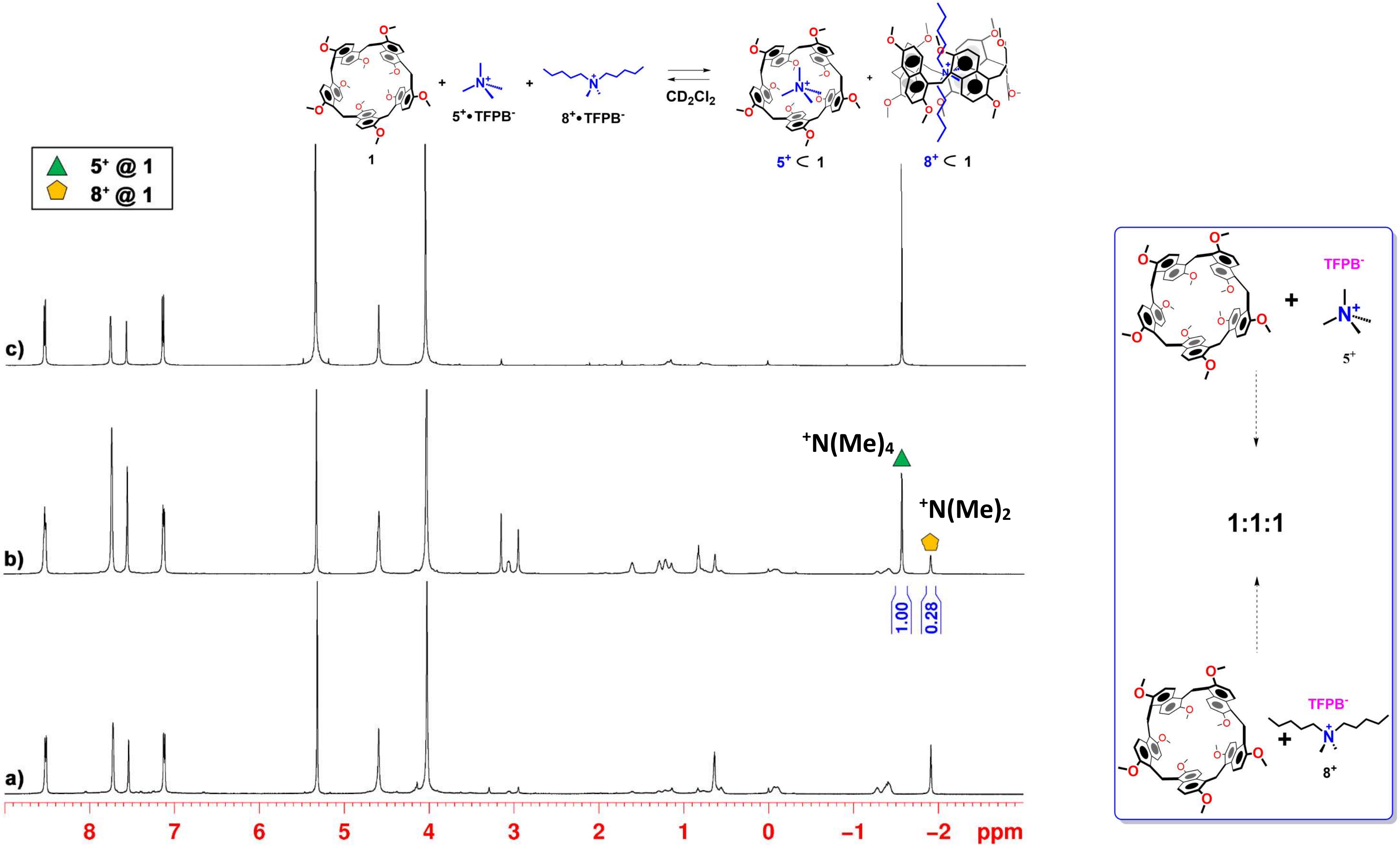

Figure S63: ^{1}H NMR spectra (600 MHz, CD_2Cl_2, 183 K) of: (a) an equimolar solution (2.85 mM) of **1** and **8$^+$·TFPB$^-$** in 0.7 mL of CD_2Cl_2, (b) of **1** in thepresence of 1 equivalent of **8$^+$·TFPB$^-$** and 1 equivalent of **5$^+$·TFPB$^-$** and (c) an equimolar solution (2.85 mM) of **1** and **5$^+$·TFPB$^-$** in 0.7 mL of CD_2Cl_2.

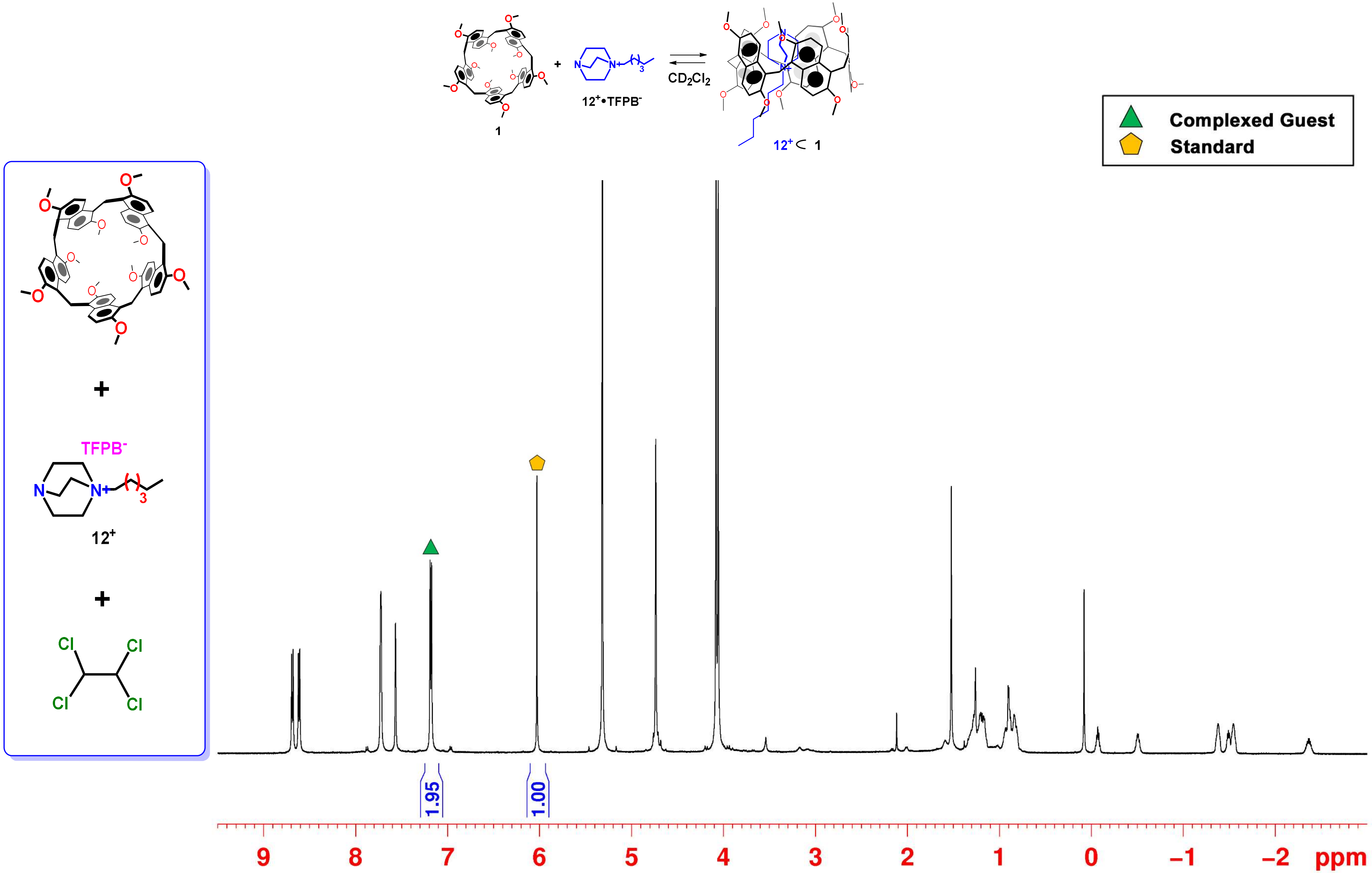

Figure S64: ^{1}H NMR spectrum (600 MHz, CD_2Cl_2, 298 K) of an equimolar solution (2.85 mM) of **1** and **12^+·TFPB$^-$** in 0.7 mL of CD_2Cl_2 containing 0.5 µL of 1,1,2,2-tetrachloroethane.

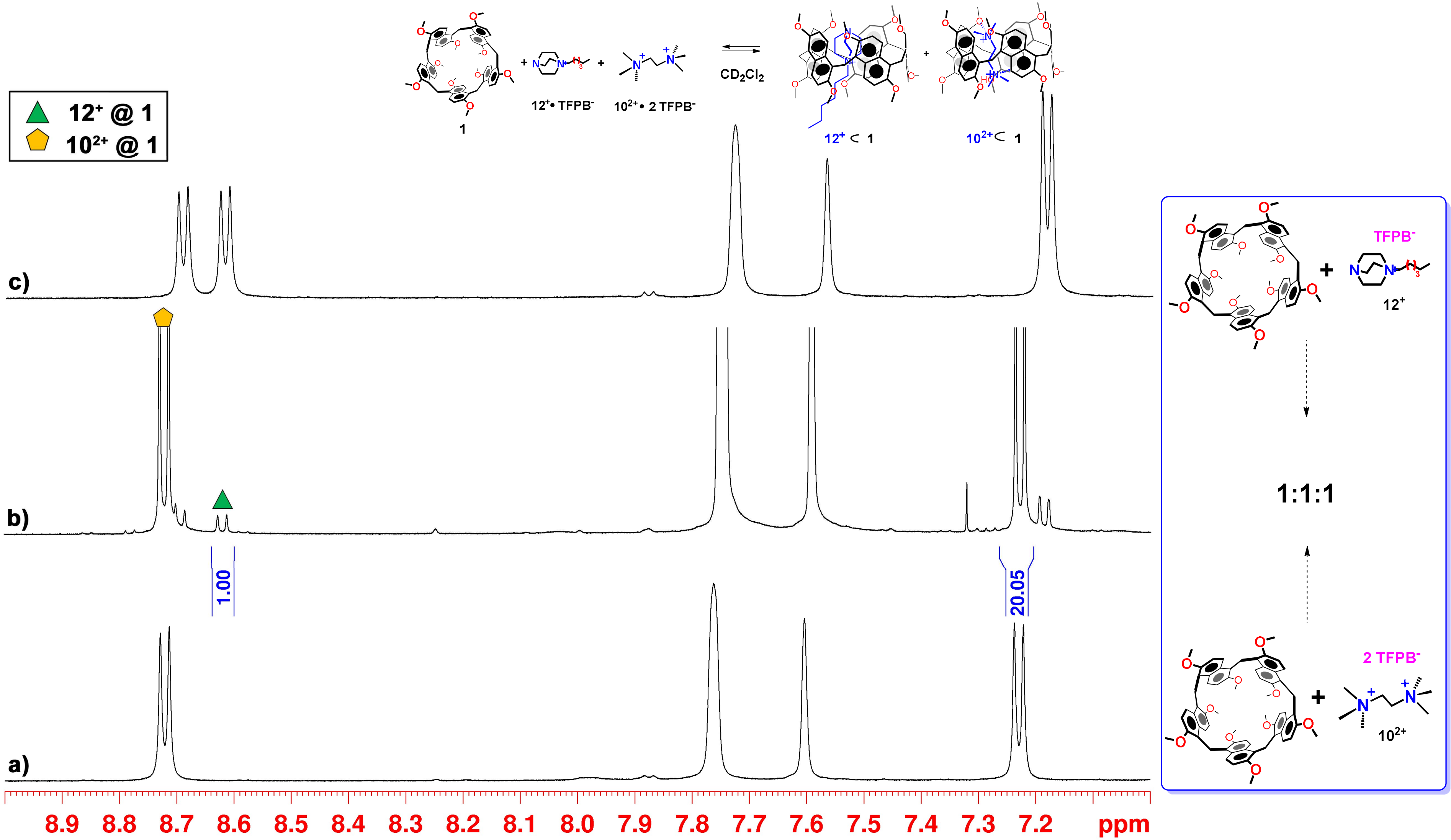

Figure S65: Significant portions of the ^{1}H NMR spectra (600 MHz, CD_2Cl_2, 298 K) of: (a) an equimolar solution (2.85 mM) of **1** and $\mathbf{10^{2+}}$**· 2 TFPB⁻** in 0.7 mL of CD_2Cl_2, (b) of **1** in the presence of 1 equivalent of $\mathbf{10^{2+}}$ **· 2 TFPB⁻** and 1 equivalent of $\mathbf{12^{+}}$**· TFPB⁻** and (c) an equimolar solution (2.85 mM) of **1** and $\mathbf{12^{+}}$ **· TFPB⁻** in 0.7 mL of CD_2Cl_2.

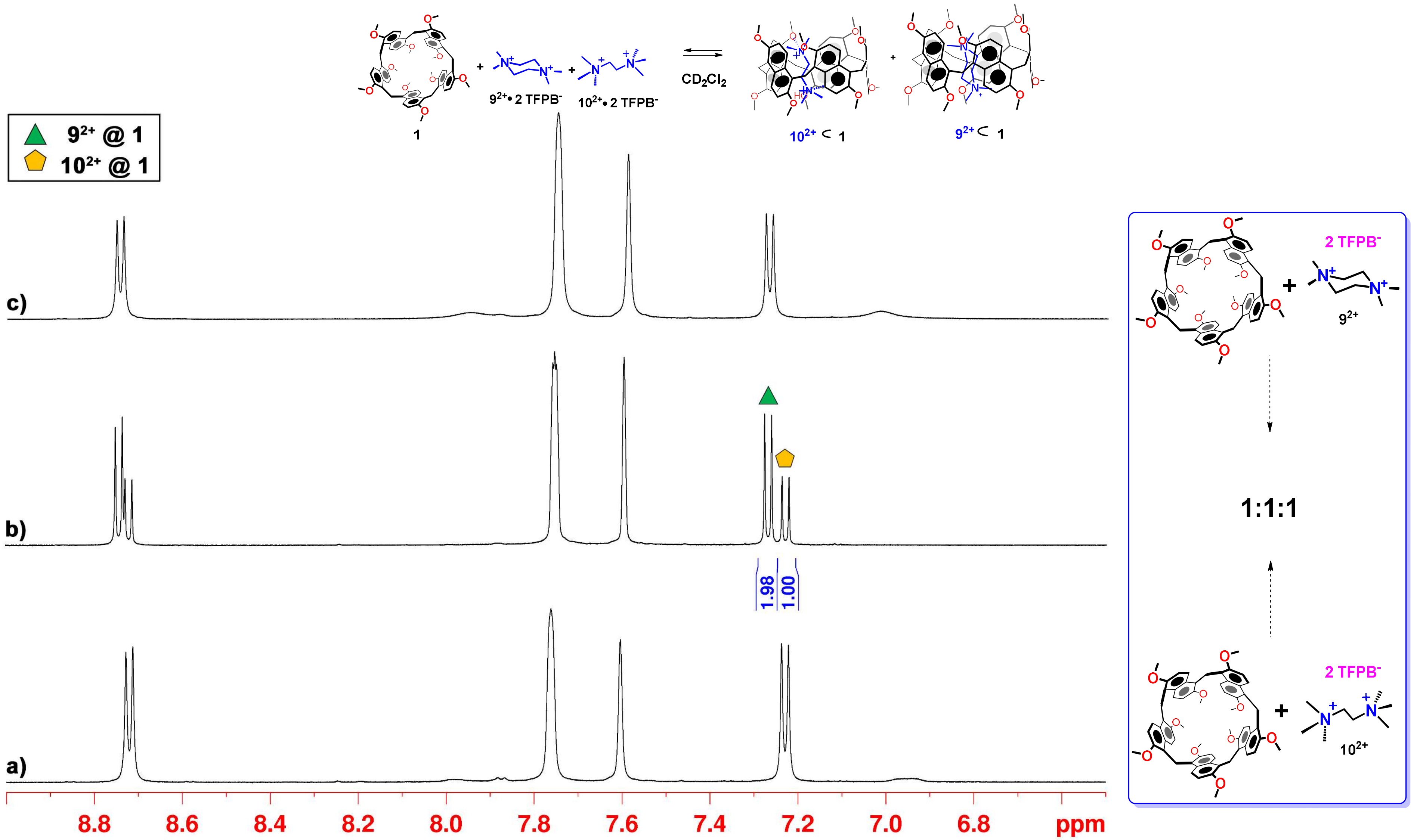

Figure S66: Significant portions of the ^{1}H NMR spectra (600 MHz, CD_2Cl_2, 298 K) of: (a) an equimolar solution (2.85 mM) of **1** and **10^{2+}· 2 TFPB$^-$** in 0.7 mL of CD_2Cl_2, (b) of **1** in the presence of 1 equivalent of **10^{2+}· 2 TFPB$^-$** and 1 equivalent of **9^{2+}· 2 TFPB$^-$** and (c) an equimolar solution (2.85 mM) of **1** and **9^{2+}· 2 TFPB$^-$** in 0.7 mL of CD_2Cl_2.

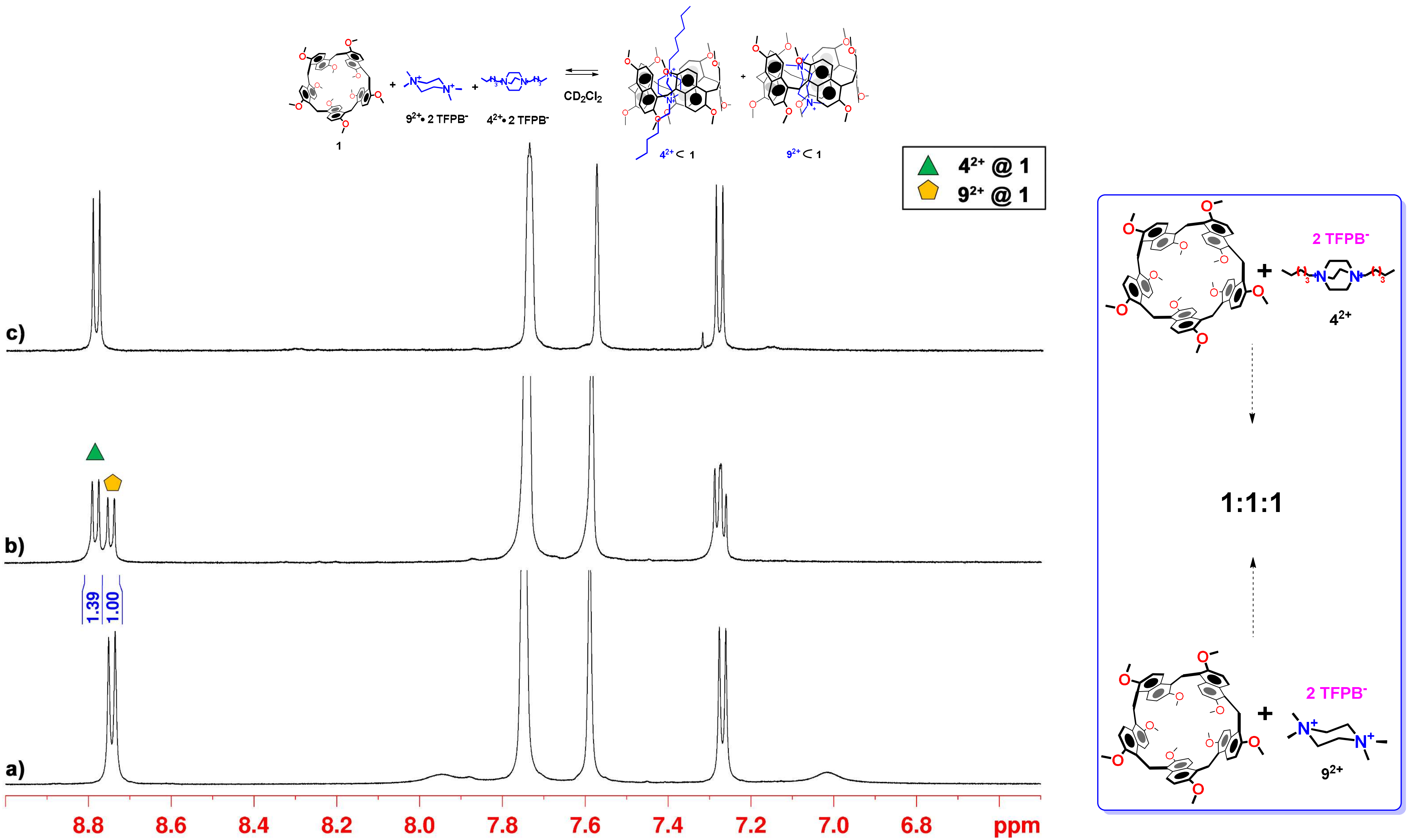

Figure S67: Significant portions of the ^{1}H NMR spectra (600 MHz, CD_2Cl_2, 298 K) of: (a) an equimolar solution (2.85 mM) of **1** and **9^{2+}· 2 TFPB$^-$** in 0.7 mL of CD_2Cl_2, (b) of **1** in the presence of 1 equivalent of **4^{2+}· 2 TFPB$^-$** and 1 equivalent of **9^{2+}· 2 TFPB$^-$** and (c) an equimolar solution (2.85 mM) of **1** and **4^{2+}· 2 TFPB$^-$** in 0.7 mL of CD_2Cl_2.

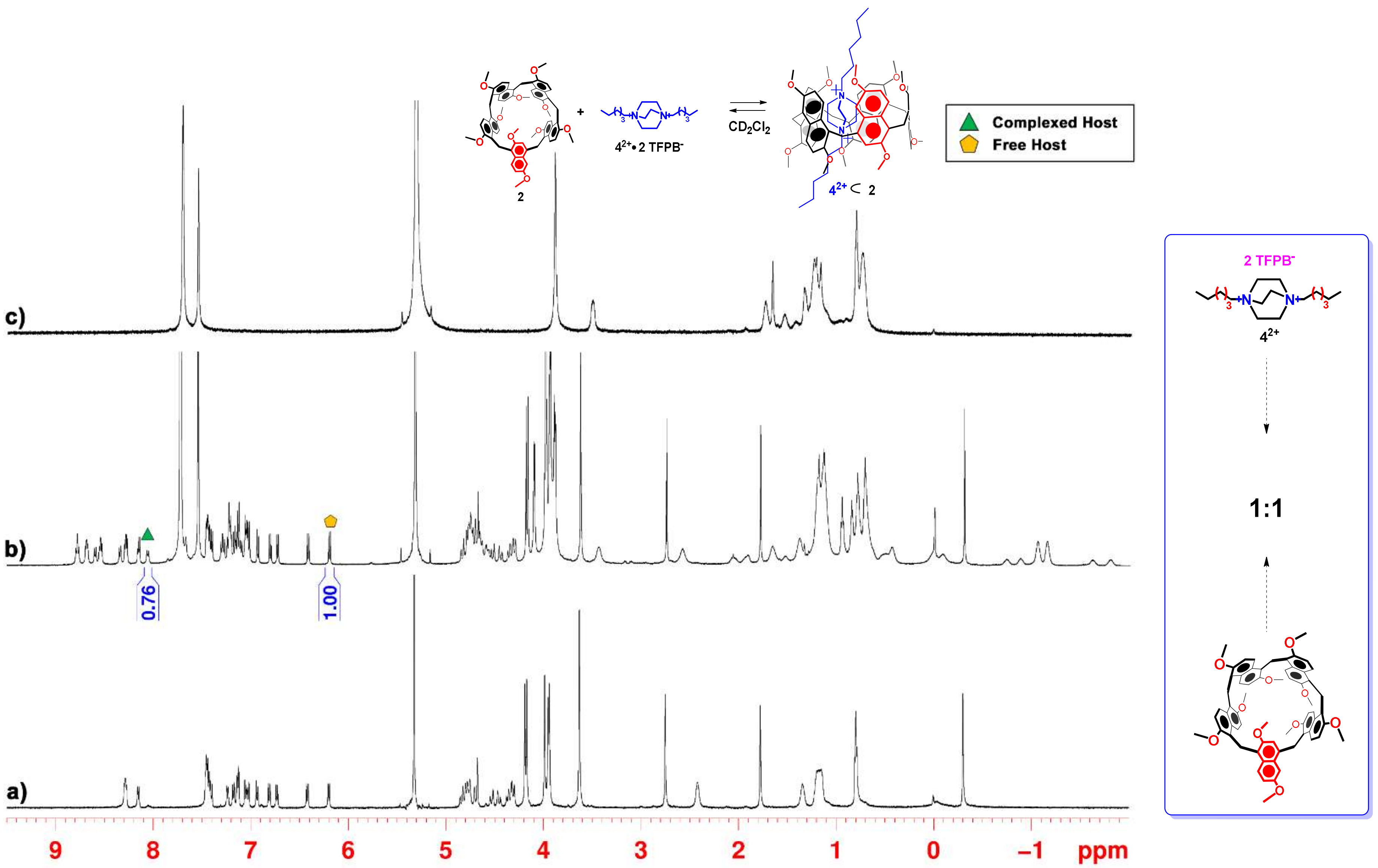

Figure S68: ^{1}H NMR spectra (600 MHz, CD_2Cl_2, 183 K) of: (a) a solution of **2** (b) an equimolar solution (2.85 mM) of **2** and **4^{2+}· 2 TFPB$^-$** in 0.7 mL of CD_2Cl_2 and (c) a solution of **4^{2+} · 2 TFPB$^-$**.

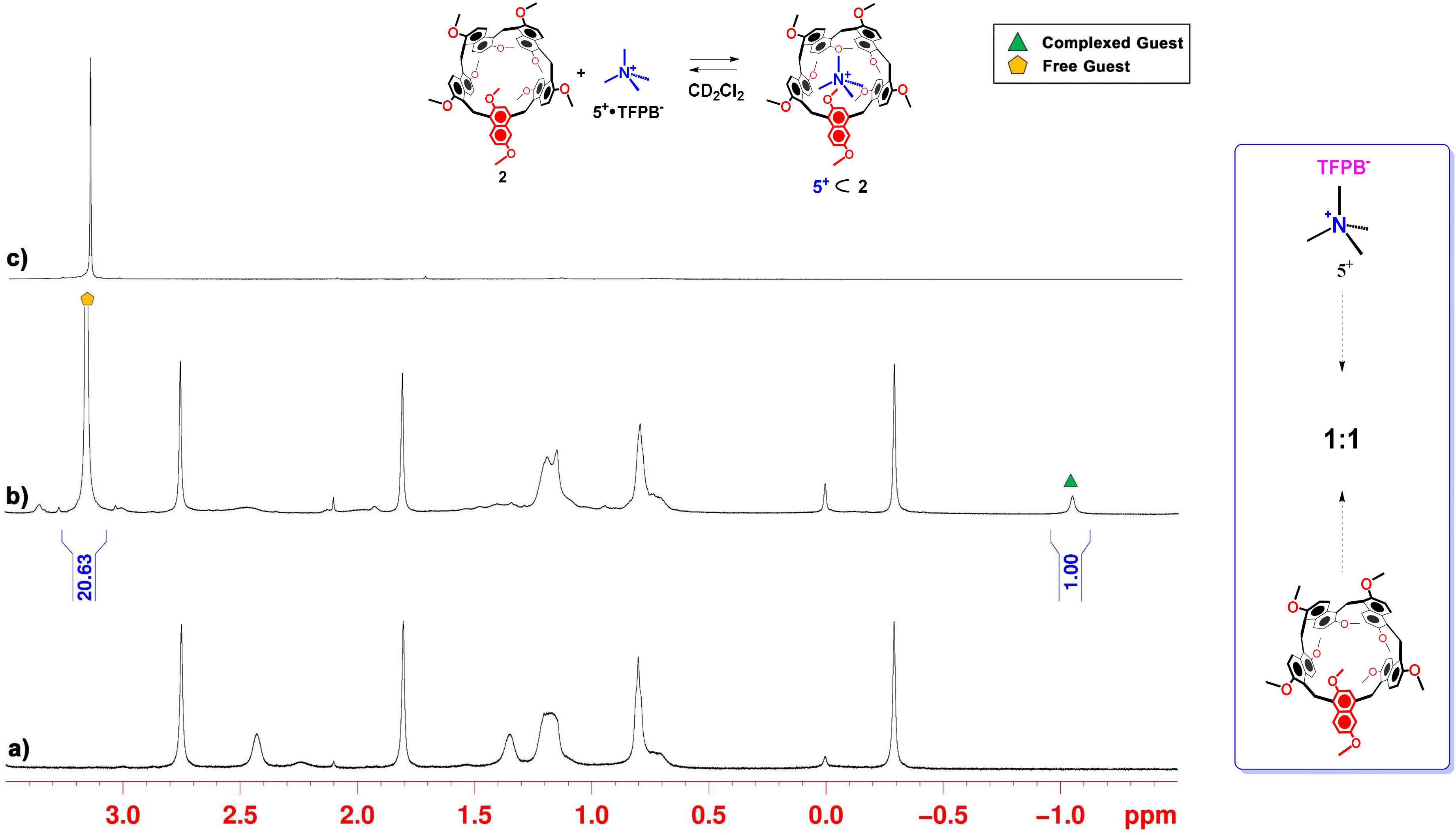

Figure S69: Significant portions of the ^{1}H NMR spectra (600 MHz, CD_2Cl_2, 183 K) of: (a) a solution of **2** (b) an equimolar solution (2.85 mM) of **2** and **5^+· TFPB$^−$** in 0.7 mL of CD_2Cl_2 and (c) a solution of **5^+· TFPB$^−$**.

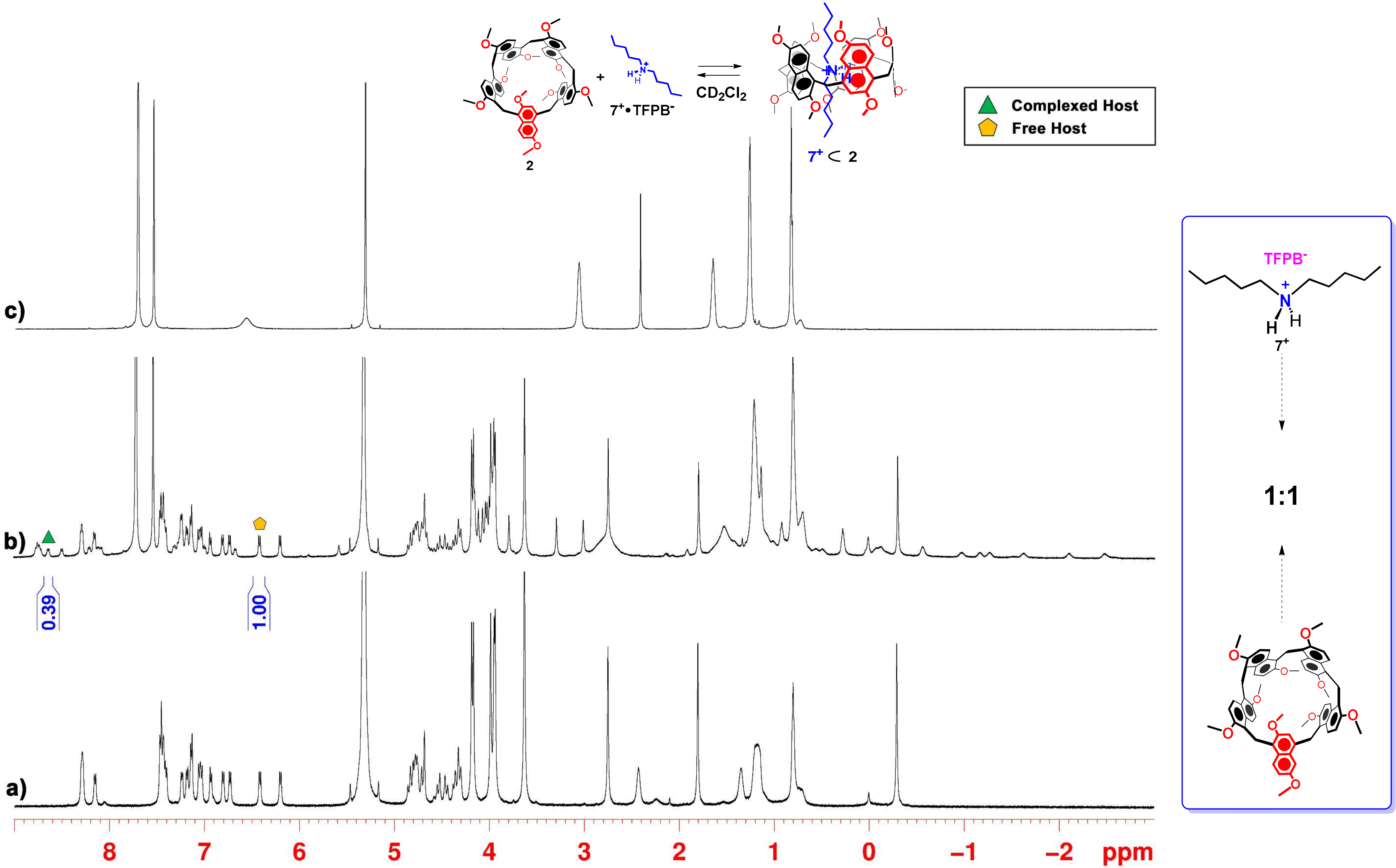

Figure S70: ^{1}H NMR spectra (600 MHz, CD_2Cl_2, 183 K) of: (a) a solution of **2** (b) an equimolar solution (2.85 mM) of **2** and **7^{+}· TFPB^{-}** in 0.7 mL of CD_2Cl_2 and (c) a solution of **7^{+}· TFPB^{-}**.

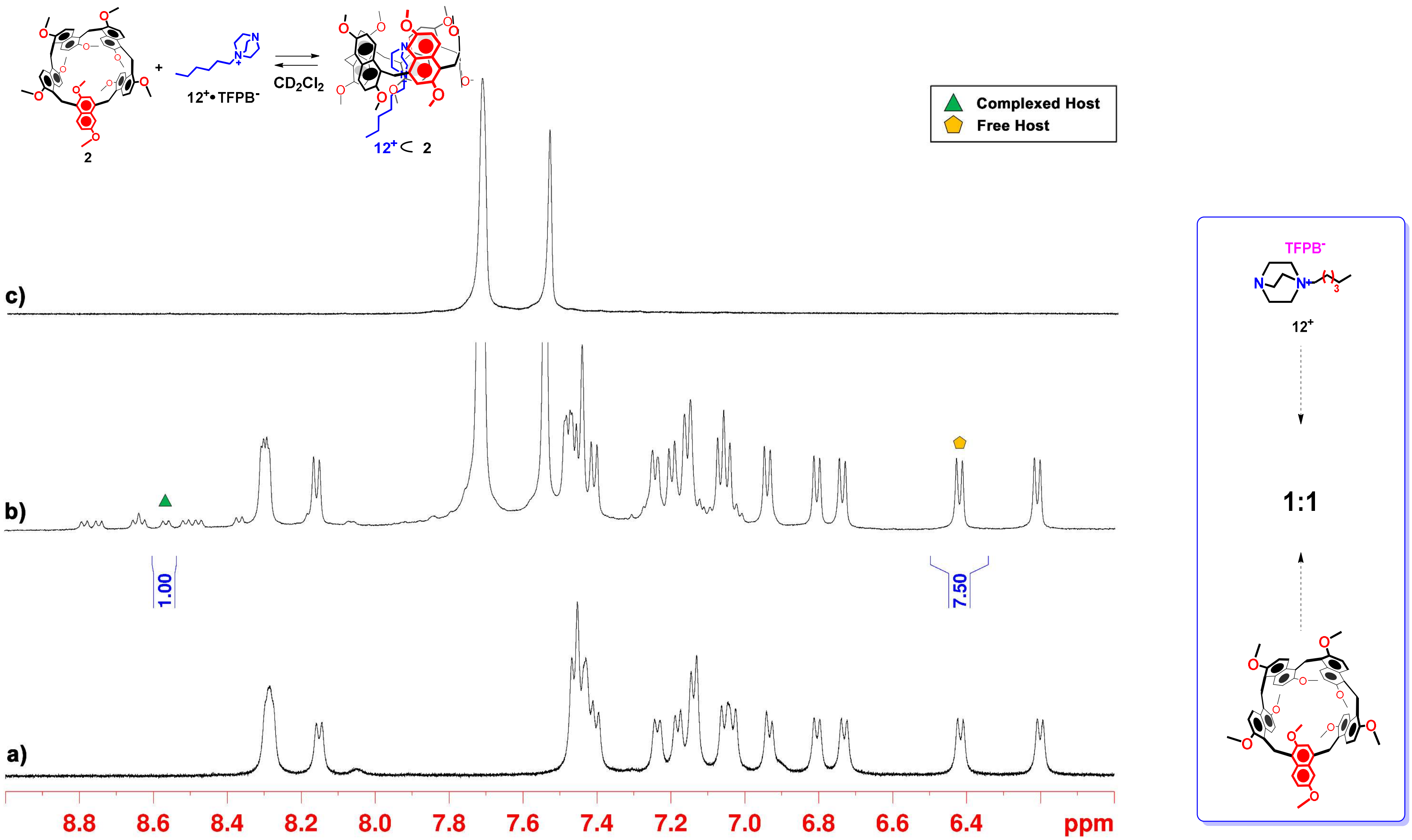

Figure S71: Significant portion of the ^{1}H NMR spectra (600 MHz, CD_2Cl_2, 183 K) of: (a) a solution of **2** (b) an equimolar solution (2.85 mM) of **2** and **12^{+}· TFPB^{-}** in 0.7 mL of CD_2Cl_2 and (c) a solution of **12^{+}· TFPB^{-}**.

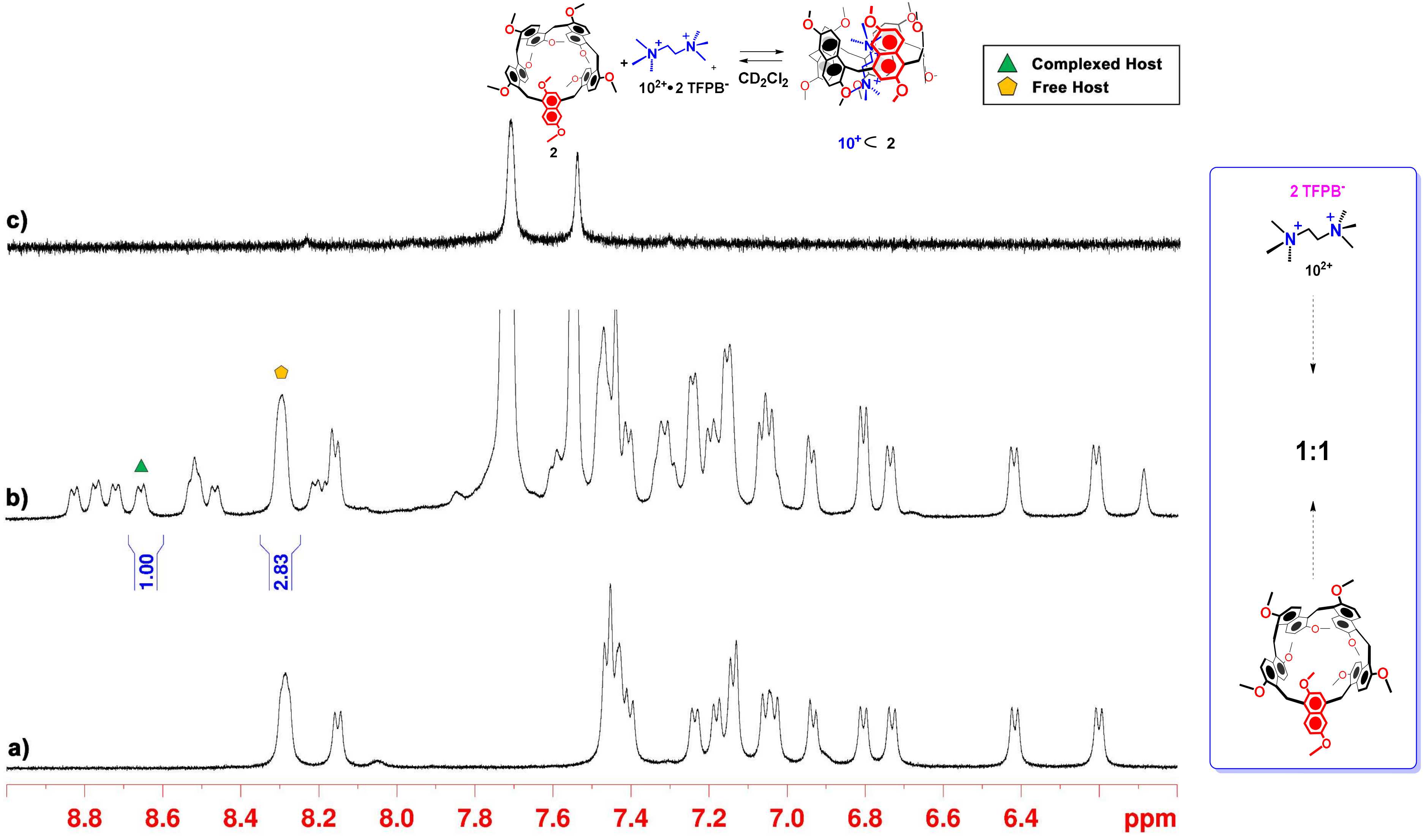

Figure S72: Significant portions of the ^{1}H NMR spectra (600 MHz, CD_2Cl_2, 183 K) of: (a) a solution of **2** (b) an equimolar solution (2.85 mM) of **2** and **10^{2+}· 2 TFPB^{-}** in 0.7 mL of CD_2Cl_2 and (c) a solution of **10^{2+}· 2 TFPB^{-}**.

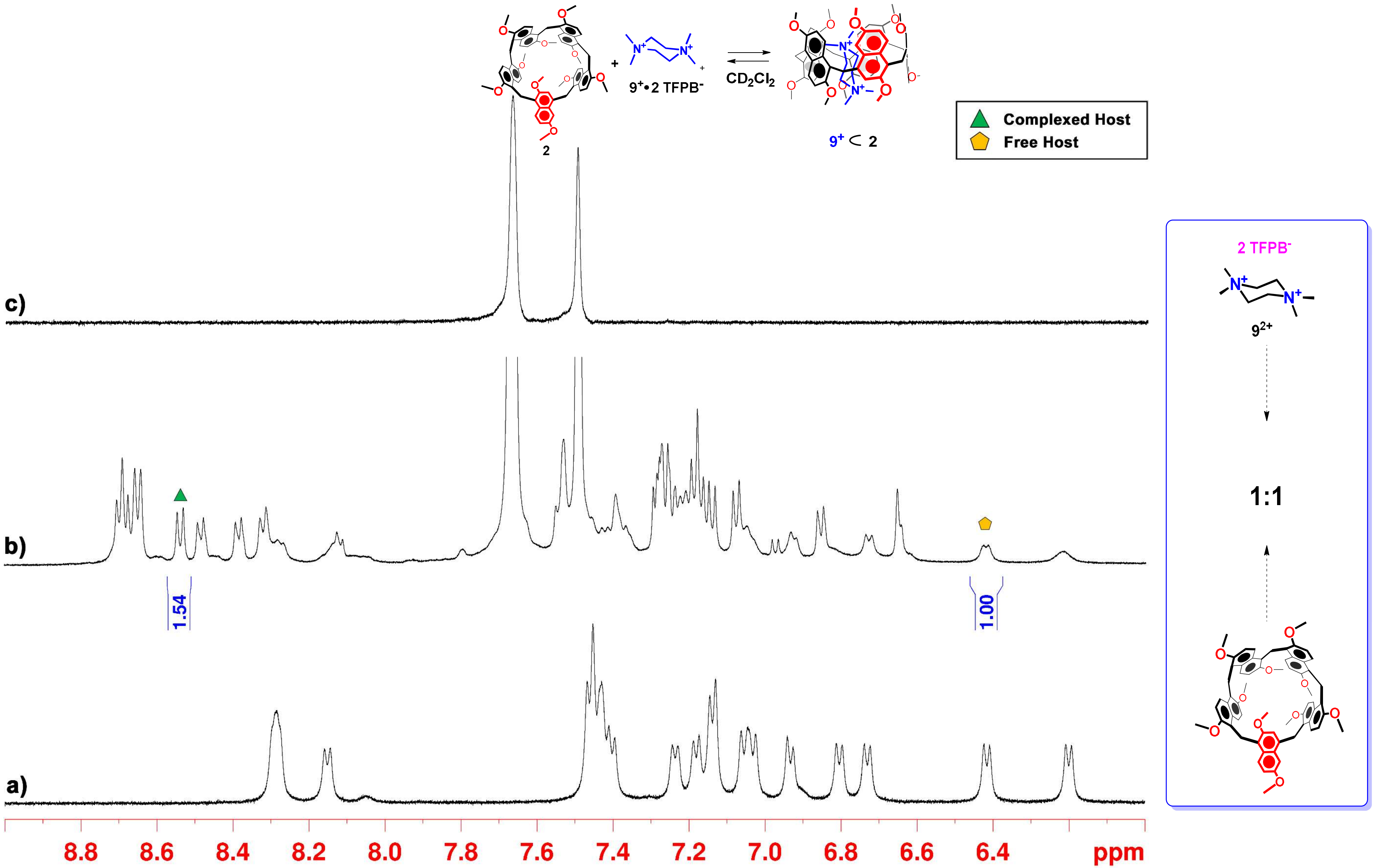

Figure S73: Significant portions of the ^{1}H NMR spectra (600 MHz, CD_2Cl_2, 183 K) of: (a) a solution of **2** (b) an equimolar solution (2.85 mM) of **2** and **9^{2+}· 2 TFPB^{-}** in 0.7 mL of CD_2Cl_2 and (c) a solution of **9^{2+}· 2 TFPB^{-}**

HPLC analysis of the cyclocondensation kinetics

All samples were analyzed on 250 × 4.6 mm Waters Spherisorb® 10µm Silica using dichloromethane-isopropanol 99.7/0.3 v/v as the mobile phase at a flow rate of 0.8 ml/min and injecting 20µL of 10µg/mL solution of reaction mixture in dichloromethane. The retention times are as follows: **2** (7.1 min) and **1** (11.0 min).

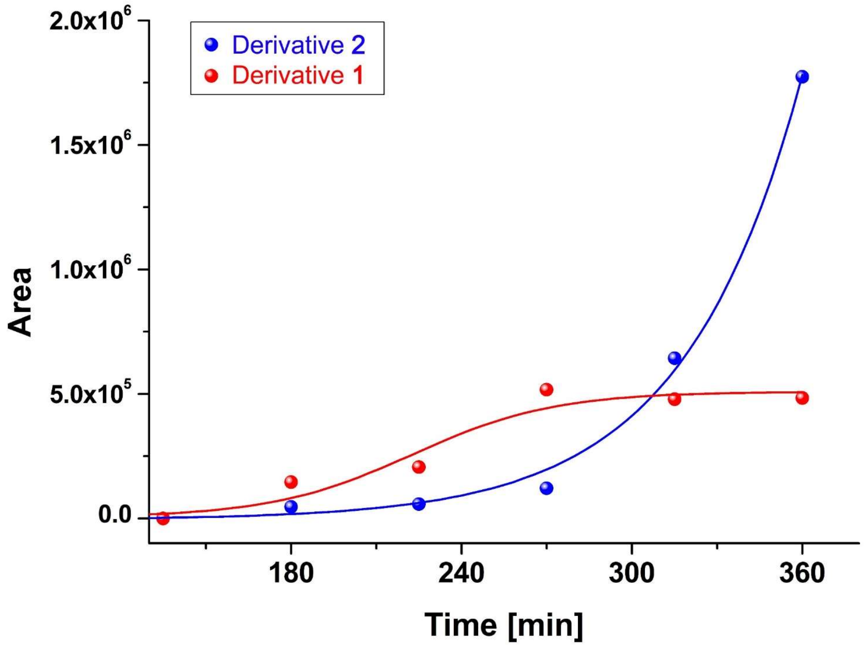

Figure S74: Relationship between time and products distribution in the cyclocondensation reactions without templanting agent.

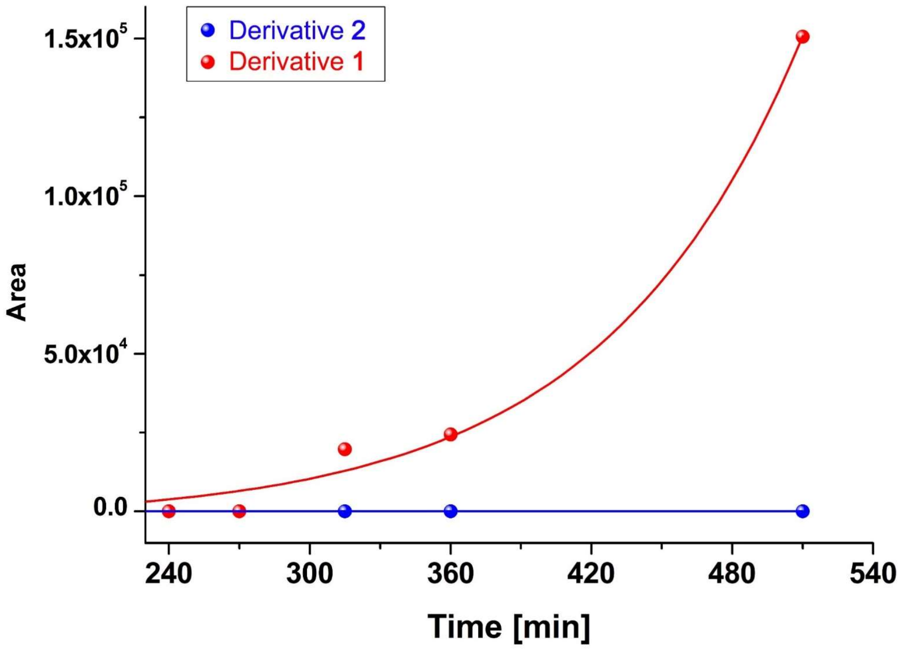

Figure S75: Relationship between time and products distribution in the cyclocondensation reactions in the presence of templating agent 4^{2+}.

Determination of the crystallographic structures of Prism[5]arenes (1) and 1,4-confused-Prism[5]arenes (2)

Single crystals suitable for X-ray investigation were obtained by slow evaporation of solutions containing prism[5]arene (**1**) or 1,4-C-prism[5]arene (**2**) in various crystallisation trials. Data collection was carried out at the Macromolecular crystallography XRD1 beamline of the Elettra synchrotron (Trieste, Italy), employing the rotating-crystal method with a Dectris Pilatus 2M area detector. Single crystals investigated were dipped in a cryo-protectant (PEG200 or Paratone), mounted on a loop and flash-frozen under a liquid nitrogen stream at 100 K. Further details of the crystallisation conditions and the cryoprotectant used are outlined below. Diffraction data were indexed and integrated using the XDS package,[5] while scaling was carried out with XSCALE.[6] The structures were solved using the SHELXT package;[7] and structure refinement was performed with SHELXL-14,[8] operating through the WinGX GUI,[9] by full-matrix least- squares (FMLS) methods on F^2.

Three pseudo-polymorphic forms of prism[5]arene (α, β and γ, space groups P-1, P-1 and C2/c, respectively) and two pseudo-polymorphic forms of 1,4-confused-prism[5]arene (α and β, space groups $P2_1/c$ and P-1, respectively) were analysed. PEG200 was used as cryoprotectant for both forms of 1,4-confused-prism[5]arene and the γ form of prism[5]arene, while paratone was used for the other two forms of prism[5]arene. For all the reported structures, the thermal parameters of the non-hydrogen atoms were refined anisotropically with the exception of atoms at or below 50% occupancy, which were refined isotropically. All hydrogen atoms were placed at the geometrically calculated positions and refined using the riding model. Crystal data and final refinement details for the structures are reported in Tables **S2** and **S3**

Prism[5]arenes

The three prism[5]arenes pseudo-polymorphs were obtained as colourless crystals in separate crystallisation trials. The solvent systems used were CH_2Cl_2 / hexane for the α form and CH_2Cl_2 / ethyl acetate for the β and γ forms. In the case of the β form, dipentylammonium hexafluoride salt was also added to the crystallisation solution. In all three crystalline forms the asymmetric unit contains one molecule of prism[5]arene **1** with a co-crystallized CH_2Cl_2 solvent molecule encapsulated in the prismarenes ring. The three pseudo-polymorphs are distinguished by a different amount of co-crystallized CH_2Cl_2 solvent molecules located between the packed molecules of **1** outside the prismarenes rings. In the case of the α form, the asymmetric unit contains two additional external CH_2Cl_2 molecules; while in the β form there is just one external site partially occupied by a disordered CH_2Cl_2. Finally, γ form contains the encapsulated CH_2Cl_2 molecule only. In the case of the β form, both CH_2Cl_2 molecules are disordered over two positions, with 90% /10% occupancy for the encapsulated molecule and 80% /10% occupancy for the partially occupied external site. For the γ form, the encapsulated CH_2Cl_2 molecule is disordered over two positions related by the crystallographic two-fold axis and it is therefore refined with 50% occupancy factor.

1,4-C-Prism[5]arenes

The two pseudo-polymorphic forms of 1,4-C-prism[5]arene, **2** were obtained as white crystals. The solvent systems used were CH_2Cl_2 / decaline for the α form and CH_2Cl_2 / pyridine for the β form. The structure refinements of both crystal forms were refined as two-component non-merohedral twins. The twin laws (1, 0, 0; 0, -1, 0; -0.052, 0, -1), which corresponds to a twofold axis about the [1 0 0] direct lattice direction in the α form, and (-1, 0, 0; -0.164, 1, -0.893; 0, 0, -1), which corresponds to a twofold axis about the [0 5 -2] direct lattice direction in the β form, were detected with PLATON TwinRoMat. The fraction of overlapped reflections was calculated at 13% and 18% for the α and β forms, respectively. The refinement as a 2-component twin with a HKLF5 reflection file reduced the R-factor significantly in both cases. The R_1 factors without twin refinement were 0.1526 and 0.1704 for the α and β forms, respectively, to compare with the twin refinement values of 0.0890 and 0.1213, respectively. The twin component is similar for the α and β forms (refined BASF factors 0.43(5) and 0.43(4) for α and β forms, respectively).

The asymmetric unit of the monoclinic α form is composed of two molecules of **2**. Both forms show a similar disorder in the position of the methoxy groups of the 1,4-naphthalene ring, corresponding to an overlap of two positions of the molecule related by a pseudo-twofold axis passing through the 1,4-naphthalene ring and the opposite methylene bridge, and which differ in the positions of the two methoxy groups of the 1,4-naphthalene ring. For both independent molecules the occupancy factor ratio of this disorder is 80/20 %. For one of the independent molecules the methyl group of one of the two methoxy substituents is further disordered over two positions, refined at 50% and 30% of full occupancy. Furthermore, the methyl group of a methoxy substituent on the other independent molecule - specifically the methoxy group of naphthalene C' (see Figure S82 for designation of naphthalene rings) tilted towards the 1,4-naphthalene ring - is disordered over two positions, refined at 70% and 30% occupancy.

The asymmetric unit of the triclinic β form is composed of one molecule of **2** with residual electron density attributed to severely disordered dichloromethane solvent molecules. The latter was not modelled but was accounted for using the Platon squeeze tool. The residual electron density of 49 electrons/cell in a total potential solvent accessible void volume of 296.3 (10.8% of the cell volume) can be attributed to a total of 1.2 dichloromethane solvent molecules per unit cell (0.6 molecules per asymmetric unit). A similar two position disorder to that observed in the α form is present also in the β form. In this case the occupancy factor ratio is 70/30%. Furthermore, there is two-position disorder (65 % and 35% occupancy factors) of the naphthalene C methyl group which points towards the 1,4-naphthalene ring.

Prismarene conformations

As all the crystalline forms of prism[5]arene, **1** and 1,4-confused-prism[5]arene, **2** are racemic mixtures, to compare the conformations of the prismarene the same S entiomer has been investigated. In Figure S82 the prismarene conformation of all crystallographically independent S enantiomers are viewed orthogonally with respect to the mean plane defined by the five methylene bridges. The dihedral angles, θ between the mean plane of the bridging methylene groups and the mean planes of the 5 naphthalene rings are reported in Table S5. The dihedral angles reported indicate the outward (> 90°) or inward (< 90°) tilt of the mean plane of the naphthalene moities, viewed from the top as in Figure 83. Intramolecular distances, *d*, between methylene bridge and the baricenter of the opposite naphthalene ring for the crystalline forms of prism[5]arene **1** and 1,4-C-prism[5]arene **2** are reported in Table S6. The naphthalene rings are labeled as in Figure S82 starting from the ring, **A** through which the twofold or pseudo-twofold axis passes, followed by the adjacent rings **B** and **B'** and the opposite rings **C** and **C'**.

In comparison with the α and β forms, the γ form of prism[5]arene, **1** shows a more open conformation of the prismarene macrocycle. In the γ form, the prism[5]arene molecules lie on a crystallographic twofold axis which passes through a naphthalene aromatic ring (**A**) and a bridging methylene group on the opposite side. As indicated by the dihedral angles, θ (Table S5), **A** is orthogonal (90°), and the adjacent **B** and **B'** rings are slightly tilted, with complementary angles, towards the centre of the cycle (9.7° tilt from orthogonality), while **C** and **C'** are significantly tilted (ca. 30 ° in opposite directions, Table S5). As clear from Figure S82 and indicated by the intramolecular distances, *d* (Table S6) the conformation of the prismarene in γ is more close to a regular pentagon with respect to the α and β forms. The overall results in a rather open prismarene ring with a hole in the centre of molecules. The α and β forms are more distorted with respect to the γ form, with partial loss of the twofold axis. The naphthalene moieties **C** and **C'** are much more inclined towards the center of the macroring in the α and β forms. In particular naphthalene 3 is tilted by more than 40°. The overall result is that the hole observed for the γ form is closed in one side by one of the methoxy groups of **C**, leading to the formation of a cavity.

A similar analysis is presented for the two pseudo-polymorphic forms 1,4-C-prism[5]arene. For these structures, **A** is also defined as the naphthalene moiety on the pseudo-twofold axis and it therefore corresponds to the 1,4-bridged naphthalene. In this case all three crystallographic independent molecules show very similar conformations. The most significant observation in all cases is the very acute angle which **A** makes with the mean plane of the prismarene macrocycles. In fact, the 1,4-bridged naphthalene lies almost flat into the centre of the macrocycle, thereby completely filling the cavity.

Table S3: Crystal data and structure refinement for the three crystalline forms of **prism[5]arene 1**

	**1**		
	α form	β form	γ form
Empirical formula	$(C_{65}H_{60}O_{10})$, 3 (CH_2Cl_2)	$(C_{65}H_{60}O_{10})$, 1.9 (CH_2Cl_2)	$(C_{65}H_{60}O_{10})$, (CH_2Cl_2)
Formula weight	1255.91	1162.49	1086.06
Temperature (K)	100(2)	100(2)	100(2)
Wavelength (Å)	0.7	0.7	0.7
Crystal system	Triclinic	Triclinic	Monoclinic
Space group	P -1	P -1	C 2/c
Unit cell dimensions (Å, °)	*a* = 13.171(3)<br>*α* = 76.093 (7)<br>*b* = 13.4040(13)<br>*β* = 80.772(6)<br>*c* = 17.6730(11)<br>*γ* = 88.492(4)	*a* = 10.3230(15)<br>*α* = 107.381(3)<br>*b* = 13.7720(8)<br>*β* = 86.09(1)<br>*c* = 21.1170(6)<br>*γ* = 99.769(10)	*a* = 26.397(2)<br>*α* = 90<br>*b* = 12.1790(3)<br>*β* = 96.210(1)<br>*c* = 16.2740(6),<br>*γ* = 90
Volume (Å^3)	2989.2(7)	2823.5(5)	5201.2(9)
Z	2	2	4
ρ_{calcd} (g/cm^3)	1.395	1.367	1.387
μ (mm^{-1})	0.330	0.249	0.188
F(000)	1312	1220	2288
Reflections collected	47654	53515	49253
Independent reflections	23371<br>[R(int) = 0.0169]	15564<br>[R(int) = 0.0184]	7601<br>[R(int) = 0.0468]
Data / restraints / parameters	13771 / 0 / 768	15564 / 0 / 765	7601 / 0 / 372
GooF	1.052	1.023	1.026
R_1 / wR_2 [I>2σ(I)]	0.0436 / 0.1143	0.044 / 0.1164	0.0528 / 0.1324
R_1 / wR_2 all data	0.0468 / 0.1149	0.0492 / 0.1209	0.0729 / 0.1477
Largest. Diff. peak/hole (e Å^{-3})	0.872 / -0.962	0.674 / -0.607	0.849 / -0.543
CCDC code	1950823	1950824	1950825

Table S4: Crystal data and structure refinement for the two crystalline forms of **1,4-C-Prism[5]arene 2**

	**2**	
	α form	β form
Empirical formula	C_{65} H_{60} O_{10}	C_{65} H_{60} O_{10}[a]
Formula weight	1001.13	1001.13
Temperature (K)	100(2)	100(2)
Wavelength (Å)	0.7	0.7
Crystal system	Monoclinic	Triclinic
Space group	P 2_1/c	P -1
Unit cell dimensions (Å, °)	a = 16.777 (8), α = 90, b = 32.962 (13), β = 91.3 (2), c = 19.166 (7), γ = 90	a = 8.593 (3), α = 62.663 (10), b = 18.778 (6), β = 84.421 (11), c = 19.167 (5), γ = 85.31 (3)
Volume (Å^3)	10596 (7)	2732.0 (15)
Z	8	2
ρ_{calcd} (g/cm^3)	1.255	1.217
μ (mm^{-1})	0.080	0.078
F(000)	4240	1060
Reflections collected	106357	25651
Independent reflections	106357	25651
Data / restraints / parameters	106357 / 0 / 1406	25651 / 0 / 709
GooF	1.020	1.021
R1 / wR2 [I>2σ(I)]	0.0890 / 0.2247	0.1213 / 0.3136
R1 / wR2 all data	0.1535 / 0.2667	0.1935 / 0.3725
Largest. Diff. peak/hole (e Å^{-3})	0.519 / -0.362	0.761 / -0.472
CCDC code	1901808	1919471

[a] The cell also contained ca. 1.2 severely disordered dichloromethane solvent molecules with partial cccupation, which were accounted for using the Platon squeeze tool.

Table S5: Dihedreal angles, θ, between the mean plane of the bridging methylene groups and the mean planes of the 5 naphthalene rings for the crystalline forms of prism[5]arene **1** and 1,4-C-prism[5]arene **2**

Crystalline Form	Dihedreal angles, θ [°]				
	A	B	B′	C	C′
			Prism[5]arene, **1**		
α	95.7	63.9	108.1	132.3	56.3
β	94.6	65.1	109.7	132.0	53.7
γ	90.0	99.7	80.3	60.3	119.7
			1,4-C-prism[5]arene, **2**		
α [a]	37.7	101.0	77.5	49.9	129.4
	37.4	103.3	78.3	49.6	128.6
β	28.5	103.7	78.0	51.0	126.7

[a] There are 2 independent molecules in the asymmetric unit. See text and Figure S82 for the designation of the 5 naphthalene moieties A, B, B', C and C'.

Table S6. Intramolecular distances, *d,* between methylene bridge and the baricenter of the opposite naphthalene ring for the crystalline forms of prism[5]arene **1** and 1,4-C-prism[5]arene **2.**

Crystalline Form	Intramolecular distances, *d* [Å]				
	A	B	B′	C	C′
			Prism[5]arene, **1**		
α	11.27	8.82	8.53	10.12	10.64
β	11.21	8.93	8.49	10.04	10.67
γ	8.93	10.77	10.77	9.72	9.72
			1,4-C-prism[5]arene, **2**		
α [a]	9.16	10.20	10.11	8.55	8.60
	9.29	10.03	10.06	8.56	8.65
β	9.41	10.38	10.38	8.87	8.90

[a] There are 2 independent molecules in the asymmetric unit.
See text and Figure S82 for the designation of the 5 naphthalene moieties A, B, B', C and C'.

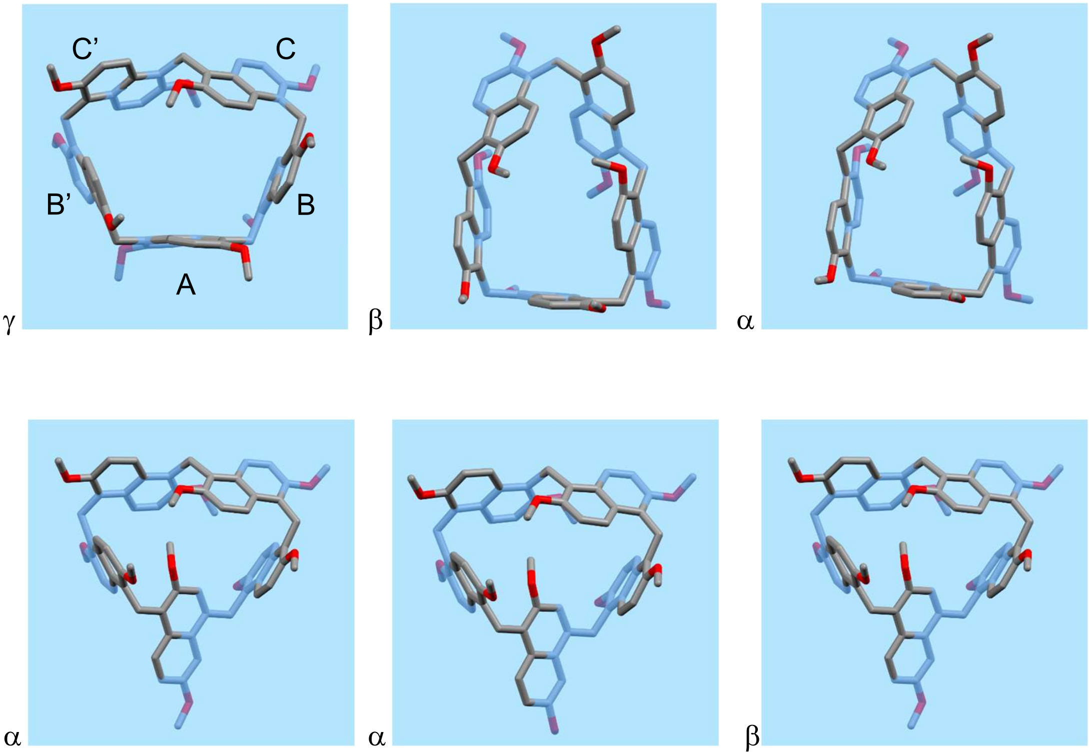

Figure S76: Prismarene conformation of all crystallographically independent S enantiomers of prism[5]arene, **1** (top row) and 1,4-C-prism[5]arene, **2** (bottom row), viewed orthogonally with respect to the mean plane defined by the five methylene bridges. Hydrogen atoms, solvent molecules and disordered groups are omitted for clarity. The naphthalene ring labeling scheme of the top left pannel is applied to all molecules.

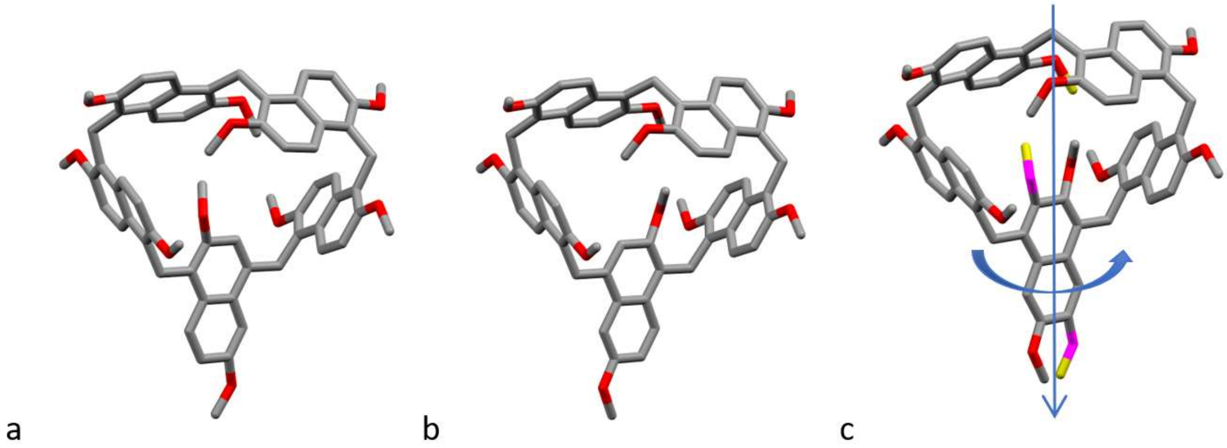

Figure S77: Two position disorder observed in both crystal forms of 1,4-C-prism[5]arene, **2.** The molecules of 1,4-C-prism[5]arene oriented as (a) and 180° rotated around the pseudo-two-fold axis (b) are overlapped in the crystal structures with an observed occupational disorder of only two methoxy groups of the 1,4-naphthalene ring (c)**.** Hydrogen atoms are omitted for clarity.

Equilibrium geometries (Cartesian coordinates, Å) and electronic energies (Hartree, include nuclear repulsion)

```
Most stable transition state for the 1,4 macrocyclization step without DCE.
Energy = -3261.216052
 C     2.432377     3.889010     0.000789
 C    -0.276461     4.493678     0.765525
 C    -0.012101     3.765697    -0.469073
 C     1.351406     3.470592    -0.835407
 C     1.566669     2.824080    -2.090656
 H     2.584805     2.627056    -2.432733
 C    -1.071001     3.380848    -1.301960
 H    -2.106446     3.611079    -1.028293
 C     3.881037     3.563417    -0.317657
 H     4.512959     4.028148     0.456373
 H     4.134528     4.066301    -1.268825
 O     3.227929     5.123315     1.853734
 C     3.032095     5.873389     3.047528
 H     2.573474     6.860125     2.831550
 H     2.397273     5.316784     3.768263
 H     4.037140     6.022423     3.477598
 O    -1.904842     2.510739    -3.309691
 C    -1.741098     2.154454    -4.685283
 H    -1.257355     1.163774    -4.796121
 H    -2.760596     2.106734    -5.103058
 H    -1.149242     2.923114    -5.220593
 C     2.149918     4.717065     1.132014
 C     0.838664     5.081042     1.445328
 C     0.516207     2.497577    -2.934958
 C    -0.824397     2.772158    -2.544485
 C     4.259520     2.093329    -0.454236
 C     4.741539     1.620151    -1.682702
 C     5.152302     0.266785    -1.822914
 H     5.557198    -0.096579    -2.772765
 C     5.053184    -0.612295    -0.758873
 H     5.400299    -1.636816    -0.901500
 C     4.548496    -0.202710     0.508534
 C     4.191085     1.194343     0.662207
 C     3.766287     1.627162     1.950405
 H     3.520717     2.678522     2.124256
 C     3.672738     0.741463     3.010092
 H     3.352119     1.119739     3.985800
 C     3.982320    -0.637966     2.841183
 C     4.399774    -1.131447     1.595290
 C     4.674444    -2.625519     1.409973
 H     5.704834    -2.764308     1.041372
 H     4.631362    -3.111005     2.398820
 O     3.894732    -1.532639     3.872675
 C     3.311207    -1.136354     5.105412
 H     2.276915    -0.759015     4.959978
 H     3.284637    -2.044308     5.733084
 H     3.919445    -0.359874     5.614485
 O     4.784921     2.512643    -2.719347
 C     5.258139     2.097238    -3.993754
 H     4.631254     1.283503    -4.414412
 H     5.189572     2.985705    -4.645361
 H     6.314109     1.760756    -3.942013
 C     3.721428    -3.320229     0.438728
 C     4.194534    -3.803353    -0.788718
 C     3.289960    -4.298030    -1.769575
 H     3.660700    -4.651453    -2.736767
 C     1.929004    -4.319524    -1.528422
 H     1.269359    -4.670380    -2.326320
 C     1.385845    -3.922901    -0.271352
 C     2.322969    -3.471389     0.740541
 C     1.814174    -3.212103     2.044752
 H     2.492547    -2.901659     2.843365
 C     0.468924    -3.363358     2.339743
 H     0.123872    -3.180935     3.362673
 C    -0.454596    -3.721047     1.321688
 C    -0.021808    -3.975772     0.010489
 C    -1.076193    -4.269104    -1.056270
 H    -0.594115    -4.643196    -1.973062
 H    -1.730942    -5.077556    -0.694729
 O    -1.796372    -3.828174     1.548676
 C    -2.326425    -3.608504     2.845333
 H    -2.109661    -2.582345     3.208351
 H    -3.419240    -3.730997     2.743631
 H    -1.934543    -4.346801     3.576273
 O     5.547035    -3.752065    -1.005453
 C     6.075718    -4.108611    -2.274476
 H     5.659594    -3.470160    -3.082246
 H     7.165438    -3.944989    -2.204497
 H     5.881888    -5.175518    -2.511251
 C    -1.905263    -3.035605    -1.408097
 C    -1.265251    -1.977453    -2.073778
 C    -1.949051    -0.764618    -2.344645
 H    -1.434879     0.051882    -2.852910
 C    -3.253852    -0.580683    -1.921813
 H    -3.724654     0.392758    -2.093229
 C    -3.955297    -1.607524    -1.229607
```

C	-3.279729	-2.875985	-1.030460
C	-4.020035	-3.909710	-0.382699
H	-3.577332	-4.901119	-0.255275
C	-5.287924	-3.693782	0.123029
H	-5.810799	-4.522543	0.610471
C	-5.879684	-2.398462	0.063396
C	-5.225596	-1.355233	-0.603736
C	-5.807958	0.038027	-0.534578
H	-5.907838	0.450946	-1.560865
H	-6.845418	-0.030218	-0.168039
O	-7.069791	-2.108016	0.667709
C	-7.754271	-3.114237	1.397924
H	-8.052417	-3.963133	0.747115
H	-7.139211	-3.497410	2.239706
H	-8.662145	-2.631583	1.801108
O	0.035204	-2.184296	-2.427679
C	0.794502	-1.151198	-3.029931
H	0.824090	-0.239857	-2.399477
H	1.818861	-1.552040	-3.130194
H	0.399193	-0.888437	-4.034382
C	-5.089675	1.125946	0.275387
C	-5.720795	2.393141	0.270843
C	-5.091748	3.527702	0.838831
H	-5.596920	4.498391	0.849847
C	-3.791988	3.439534	1.327859
H	-3.306935	4.353251	1.689538
C	-3.088874	2.213198	1.304878
C	-3.800121	1.025857	0.882489
C	-3.113460	-0.221704	1.084331
H	-3.677578	-1.151826	1.114993
C	-1.742466	-0.283069	1.224115
H	-1.242239	-1.254066	1.263328
C	-0.995239	0.919646	1.299285
C	-1.671781	2.134861	1.602571
C	-0.892936	3.213721	2.143966
H	0.079603	2.908615	2.546592
H	-1.421567	3.939241	2.776231
O	0.332736	0.979253	1.162312
C	1.040133	-0.166747	0.666376
H	1.975381	0.224219	0.243416
H	1.262713	-0.868258	1.487323
H	0.455370	-0.680702	-0.116477
O	-6.937312	2.446458	-0.336130
C	-7.631311	3.683684	-0.425115
H	-7.044673	4.436815	-0.991732
H	-8.568361	3.466359	-0.966873
H	-7.874455	4.085952	0.580376
H	0.630462	5.739789	2.294406
H	0.734719	2.040390	-3.904957
H	-1.210092	5.073572	0.770573

Most stable transition state for the 1,5 macrocyclization step without DCE.
Energy = -3261.221317

C	1.688615	-2.530204	2.208131
C	-0.436518	-2.007651	0.411943
C	-0.556258	-1.693309	1.794264
C	0.518839	-1.958466	2.679634
H	2.499403	-2.701754	2.920650
H	0.445341	-1.696388	3.739206
C	1.837687	-2.954382	0.853063
C	0.724442	-2.752817	-0.037507
C	0.806022	-3.316145	-1.335768
C	1.919172	-4.050336	-1.726722
C	3.041141	-4.188496	-0.867359
C	3.042203	-3.582535	0.401357
H	-0.030305	-3.225300	-2.036583
H	1.926280	-4.505348	-2.722159
C	4.329604	-3.456866	1.211943
H	4.212714	-3.888790	2.219763
H	5.124540	-4.034971	0.712282
O	4.165575	-4.870119	-1.228809
O	-1.703737	-1.114210	2.195335
C	4.316737	-5.322066	-2.569373
H	3.559819	-6.091400	-2.826917
H	4.248058	-4.477196	-3.286352
H	5.323796	-5.770377	-2.624709
C	-2.018261	-1.071538	3.584777
H	-3.051322	-0.697193	3.645377
H	-1.966242	-2.086885	4.025127
H	-1.343048	-0.388425	4.135808
C	-4.934606	-1.206252	-1.523305
C	-2.862034	-2.977780	-0.827196
C	-3.120700	-2.659471	-2.208209
C	-4.141527	-1.740124	-2.534386
H	-5.742521	-0.531956	-1.816659
H	-4.335668	-1.454871	-3.572516
C	-3.859495	-2.612895	0.170367
C	-4.802732	-1.578147	-0.146622
C	-5.595120	-0.978146	0.876446
C	-5.569465	-1.560234	2.160776
C	-4.742422	-2.681223	2.430943
C	-3.867275	-3.167429	1.461477
H	-4.736914	-3.137203	3.425437
H	-3.169847	-3.976915	1.710618
O	-2.295723	-3.225101	-3.091282

O	-6.335416	-0.960661	3.112453
C	-6.380652	0.299885	0.615175
H	-6.756050	0.677873	1.581883
H	-7.275336	0.073547	0.007862
C	-3.943434	3.174194	-1.570233
C	-5.163849	2.763948	-2.075946
C	-5.975700	1.847816	-1.351166
C	-5.565565	1.372803	-0.098085
H	-3.334133	3.845779	-2.180397
H	-5.483319	3.135852	-3.054514
C	-3.469297	2.737827	-0.298036
C	-4.338735	1.860916	0.463998
C	-3.935569	1.501346	1.781698
C	-2.723795	1.923739	2.299336
C	-1.835210	2.700545	1.514300
C	-2.189125	3.125522	0.226266
H	-4.601614	0.909586	2.415787
H	-2.458001	1.669990	3.328900
O	-7.143548	1.341035	-1.854205
O	-0.625796	3.117118	1.986055
H	-0.937751	4.860576	0.093017
H	-1.675727	4.405368	-1.437698
C	-1.193944	3.992779	-0.536436
C	2.399630	2.028581	-2.050114
C	0.083966	3.283818	-0.979292
C	-0.015108	2.337993	-2.011327
C	1.146127	1.728321	-2.556397
H	3.271875	1.572368	-2.519877
H	1.066513	1.027372	-3.393939
C	1.381108	3.580832	-0.435154
C	2.569225	2.926098	-0.956676
C	3.866127	3.186233	-0.393380
C	3.970684	4.101386	0.663470
C	2.818834	4.774846	1.155398
C	1.569840	4.523653	0.617444
H	2.905354	5.499013	1.971634
H	0.711967	5.048159	1.043570
O	-1.273510	2.078466	-2.475040
O	5.217538	4.301355	1.196795
C	5.134937	2.540552	-0.942898
H	5.987650	2.920440	-0.354179
H	5.290700	2.899056	-1.975393
C	5.186661	-1.816022	-1.105386
C	5.400354	-1.063931	-2.247325
C	5.376270	0.355340	-2.189648
C	5.186181	1.019860	-0.967641
H	5.218515	-2.903882	-1.188840
H	5.578041	-1.581332	-3.195658
C	5.044906	0.240724	0.227937
C	4.976644	-1.202846	0.162110
C	4.711656	-1.982414	1.338825
C	4.685257	-1.337578	2.582174
C	4.834017	0.077029	2.664167
C	4.977635	0.843113	1.519376
H	4.821363	0.578845	3.637094
H	5.076613	1.927218	1.622515
O	5.506460	1.134870	-3.307858
O	4.490601	-2.121318	3.690210
C	-7.584186	1.731203	-3.148087
H	-6.845418	1.455818	-3.929731
H	-8.525340	1.181792	-3.325166
H	-7.782191	2.821938	-3.196284
C	-6.336727	-1.462289	4.444132
H	-7.010703	-0.799994	5.014263
H	-6.723273	-2.501481	4.482407
H	-5.321852	-1.427796	4.891762
C	-2.368967	-2.879693	-4.483848
H	-1.566267	-3.455995	-4.971825
H	-3.350988	-3.170606	-4.902966
H	-2.196932	-1.795850	-4.625415
C	0.031393	2.372639	2.999188
H	0.057628	1.292948	2.744072
H	1.064704	2.762111	3.037443
H	-0.445219	2.508189	3.993345
C	-1.467143	1.171797	-3.543041
H	-2.560717	1.092384	-3.677267
H	-1.003320	1.538582	-4.483644
H	-1.056514	0.167350	-3.308442
C	4.274424	-1.509497	4.953945
H	4.071728	-2.334408	5.659530
H	5.170488	-0.951015	5.295858
H	3.401181	-0.823627	4.926990
C	5.602559	0.525948	-4.585739
H	5.649734	1.354939	-5.313897
H	6.521329	-0.090738	-4.677004
H	4.714437	-0.103973	-4.804774
C	5.377435	5.154224	2.319198
H	6.451158	5.119780	2.575559
H	5.093549	6.200978	2.081526
H	4.782293	4.801255	3.187947
H	-2.252728	-3.872180	-0.645121
C	-1.428005	-1.534657	-0.516700
H	-2.096356	-0.738751	-0.165000
H	-1.084153	-1.404411	-1.549060

Most stable transition state for the 1,4 macrocyclization step, DCE inside the cavity.
Energy = -4259.921879

```
 C    -1.618735    -3.242040    -2.304502
 C     0.957559    -2.639637    -1.318639
 C     0.615966    -2.368457    -2.670324
 C    -0.665816    -2.719301    -3.163563
 H    -2.619629    -3.434101    -2.700070
 H    -0.937034    -2.530100    -4.206299
 C    -1.330721    -3.555604    -0.941270
 C     0.025666    -3.380537    -0.493122
 C     0.369431    -3.863793     0.792503
 C    -0.586208    -4.438981     1.617948
 C   -1.948558   -4.494595    1.220341
 C   -2.346864   -4.017450   -0.043574
 H    1.399240   -3.789704    1.158156
 H   -0.278996   -4.817728    2.597243
 C   -3.825839   -3.922043   -0.408444
 H   -4.022097   -4.490737   -1.332732
 H   -4.410716   -4.411136    0.387817
 O   -2.924336   -4.984684    2.036889
 O    1.547005   -1.729869   -3.407169
 C   -2.635398   -5.229331    3.409616
 H   -1.926620   -6.074146    3.530856
 H   -2.221498   -4.321572    3.896578
 H   -3.599164   -5.493806    3.877914
 C    1.250601   -1.349126   -4.751926
 H    2.121058   -0.769110   -5.098538
 H    1.115313   -2.242116   -5.393620
 H    0.343451   -0.715893   -4.794035
 C    5.643981   -0.549837    1.363501
 C    4.294092   -3.004961    1.606429
 C    4.788329   -2.358459    2.753298
 C    5.471439   -1.114081    2.616373
 H    6.203289    0.386132    1.287058
 H    5.879516   -0.599413    3.491689
 C    4.463450   -2.440918    0.335592
 C    5.152244   -1.183993    0.180850
 C    5.356202   -0.634962   -1.121452
 C    5.049839   -1.434316   -2.268291
 C    4.319925   -2.615713   -2.132810
 C    3.828129   -3.016550   -0.846296
 H    3.995131   -3.180067   -3.010910
 H    3.443838   -4.040866   -0.754710
 O    4.584181   -2.983331    3.926548
 O    5.448313   -0.929979   -3.465333
 C    5.890353    0.770943   -1.315682
 H    5.994309    0.959150   -2.396711
 H    6.906975    0.830176   -0.887777
 C    3.507428    3.912977    0.554184
 C    4.814797    3.688236    0.945811
 C    5.590164    2.662919    0.336876
 C    5.040035    1.866101   -0.674226
 H    2.944469    4.696259    1.065139
 H    5.243630    4.310780    1.737246
 C    2.885605    3.149809   -0.478493
 C    3.694565    2.119484   -1.111385
 C    3.121880    1.402832   -2.199584
 C    1.836568    1.666806   -2.638064
 C    1.045445    2.662173   -2.008989
 C    1.540049    3.402249   -0.921167
 H    3.705612    0.652599   -2.737784
 H    1.440624    1.112461   -3.491296
 O    6.880303    2.395667    0.711114
 O   -0.199385    2.984242   -2.448163
 H    0.242515    5.085085   -1.165341
 H    1.252443    5.184020    0.275013
 C    0.643380    4.491765   -0.327292
 C   -2.541603    2.939702    2.236157
 C   -0.511164    3.986828    0.538999
 C   -0.222653    3.562052    1.845548
 C   -1.249774    3.091419    2.706300
 H   -3.297797    2.528701    2.908824
 H   -1.020678    2.806313    3.737314
 C   -1.874025    3.919219    0.080990
 C   -2.893827    3.294953    0.903493
 C   -4.213237    3.047745    0.386880
 C   -4.553922    3.587008   -0.860760
 C   -3.594635    4.311313   -1.622380
 C   -2.293793    4.448674   -1.173869
 H   -3.869844    4.751629   -2.586049
 H   -1.581604    4.976697   -1.811608
 O    1.080594    3.644060    2.241613
 O   -5.834362    3.381920   -1.306423
 C   -5.232988    2.216430    1.163916
 H   -6.197200    2.276899    0.631834
 H   -5.397121    2.668988    2.156200
 C   -4.137538   -1.968656    1.823637
 C   -4.254094   -1.089160    2.884618
 C   -4.623035    0.266434    2.669810
 C   -4.867682    0.745659    1.372947
 H   -3.866232   -3.004121    2.037319
 H   -4.056408   -1.455770    3.895935
 C   -4.796114   -0.181936    0.274539
 C   -4.401217   -1.561363    0.485242
 C   -4.319569   -2.487550   -0.611032
 C   -4.715867   -2.053734   -1.884063
```

C	-5.115223	-0.705375	-2.097185
C	-5.137725	0.202861	-1.053241
H	-5.413768	-0.367638	-3.094778
H	-5.461386	1.225387	-1.259614
O	-4.763043	1.151482	3.705372
O	-4.707050	-2.981100	-2.894647
C	7.487397	3.150458	1.751633
H	6.936331	3.038764	2.708697
H	8.506177	2.740905	1.866193
H	7.552246	4.225747	1.485851
C	5.276042	-1.712706	-4.642587
H	5.720930	-1.124607	-5.463091
H	5.803405	-2.684434	-4.550153
H	4.204318	-1.893303	-4.856280
C	5.050506	-2.392586	5.141607
H	4.756709	-3.089553	5.944503
H	6.153192	-2.280572	5.132366
H	4.573288	-1.406651	5.312297
C	-0.740662	2.368972	-3.602987
H	-0.837133	1.272912	-3.473422
H	-1.748359	2.803803	-3.726386
H	-0.127145	2.585223	-4.503351
C	1.503127	2.987375	3.426557
H	2.606224	3.052113	3.428005
H	1.107386	3.485190	4.337193
H	1.193820	1.921721	3.427372
C	-4.996939	-2.578883	-4.225309
H	-4.887619	-3.485569	-4.846036
H	-6.034656	-2.196603	-4.318575
H	-4.285328	-1.803119	-4.579036
C	-4.359021	0.774593	5.014539
H	-4.484435	1.675742	5.640311
H	-4.995953	-0.039813	5.418835
H	-3.296322	0.457006	5.031073
C	-6.201867	3.792264	-2.614316
H	-7.247121	3.463657	-2.752364
H	-6.150139	4.895555	-2.726048
H	-5.561717	3.314423	-3.385939
C	-0.802393	-0.853931	1.456822
H	0.222282	-1.112940	1.156497
Cl	-0.733528	-0.533315	3.247210
H	-1.468900	-1.718650	1.323631
C	-1.316778	0.362851	0.720349
H	-2.331908	0.633210	1.039004
H	-0.645209	1.229004	0.813165
Cl	-1.424988	-0.021077	-1.047409
H	3.777506	-3.964616	1.726721
C	2.110867	-2.004232	-0.736941
H	2.449123	-1.086461	-1.235168
H	2.084952	-1.913798	0.354021

Most stable transition state for the 1,5 macrocyclization step, DCE inside the cavity.
Energy = -4259.928701

C	2.194551	1.866851	-1.755524
C	4.663795	0.530201	-1.315809
C	3.635508	-0.060816	-2.105158
C	2.400521	0.614089	-2.300073
H	1.225112	2.345691	-1.922608
H	1.594529	0.145203	-2.870499
C	3.195710	2.541482	-0.989963
C	4.436930	1.845362	-0.742667
C	5.416846	2.518723	0.023550
C	5.208998	3.807258	0.501916
C	4.001930	4.496845	0.229824
C	2.980016	3.865907	-0.505659
H	6.373124	2.043070	0.250430
H	6.003286	4.284306	1.084124
C	1.695379	4.613129	-0.809118
H	1.842945	5.670124	-0.523611
H	1.554663	4.614404	-1.902040
O	3.763962	5.763629	0.651636
O	3.910543	-1.240925	-2.686884
C	4.697696	6.415592	1.500629
H	4.850912	5.846860	2.441556
H	5.675047	6.565635	0.995866
H	4.256067	7.399440	1.736551
C	3.142613	-1.647993	-3.823572
H	2.097378	-1.853551	-3.538983
H	3.182628	-0.868924	-4.610948
H	3.617204	-2.574347	-4.185892
C	3.392749	-1.161936	1.369888
C	6.032473	-1.404409	0.443707
C	5.711927	-0.458088	1.476442
C	4.351760	-0.263394	1.812201
H	2.350780	-0.970405	1.650722
H	4.084666	0.582353	2.452825
C	5.080340	-2.487859	0.192426
C	3.727804	-2.372232	0.663529
C	2.822184	-3.464592	0.522799
C	3.293906	-4.651678	-0.084218
C	4.601902	-4.733857	-0.607768
C	5.480446	-3.655656	-0.473775
H	4.951736	-5.648679	-1.096113
H	6.506268	-3.741818	-0.855155
O	6.601785	0.329620	2.079790

O	2.408587	-5.680082	-0.116966
C	1.447932	-3.486619	1.154131
H	1.397050	-4.425236	1.741932
H	1.371942	-2.675199	1.898302
C	-2.256106	-3.318122	-1.175427
C	-1.039702	-3.301965	-1.822734
C	0.182561	-3.361917	-1.097106
C	0.187973	-3.434789	0.295261
H	-3.170908	-3.285634	-1.769616
H	-1.002119	-3.233966	-2.916237
C	-2.326757	-3.407549	0.246926
C	-1.082318	-3.488638	0.981796
C	-1.167235	-3.643095	2.394423
C	-2.388167	-3.742895	3.040572
C	-3.603244	-3.639026	2.313088
C	-3.588574	-3.418015	0.927025
H	-0.260616	-3.699079	3.003608
H	-2.401580	-3.883796	4.125763
O	1.366413	-3.354899	-1.795359
O	-4.827626	-3.712455	2.918990
H	-5.730284	-3.285467	0.874846
H	-5.023791	-3.890933	-0.626744
C	-4.888735	-3.146077	0.175416
C	-4.889447	0.828383	-1.640155
C	-4.954260	-1.752082	-0.444903
C	-5.042496	-1.598691	-1.835426
C	-5.011410	-0.304974	-2.424770
H	-4.861882	1.802845	-2.132639
H	-5.072844	-0.189534	-3.511676
C	-4.903967	-0.574028	0.373995
C	-4.831050	0.744757	-0.220101
C	-4.728513	1.918435	0.602899
C	-4.788754	1.767051	1.997088
C	-4.895652	0.478249	2.585015
C	-4.945535	-0.655251	1.794859
H	-4.952048	0.367192	3.672442
H	-5.058423	-1.622929	2.286105
O	-5.140614	-2.741910	-2.585480
O	-4.742585	2.906878	2.757539
C	-4.603914	3.313313	-0.003886
H	-4.716480	4.046203	0.813315
H	-5.449961	3.482236	-0.690889
C	-0.959824	4.303623	-2.207350
C	-2.184264	4.247732	-2.851061
C	-3.367884	3.929717	-2.132790
C	-3.315312	3.629193	-0.762331
H	-0.080911	4.564556	-2.805885
H	-2.226736	4.461175	-3.923652
C	-2.046651	3.691659	-0.095264
C	-0.834905	4.040386	-0.814029
C	0.428530	4.116595	-0.119349
C	0.459197	3.826549	1.246469
C	-0.724868	3.492530	1.953610
C	-1.942372	3.447481	1.306512
H	-0.666573	3.309553	3.032714
H	-2.839934	3.249737	1.892107
O	-4.597127	3.899007	-2.732401
O	1.642080	3.950886	1.936153
C	1.500392	-4.360053	-2.799653
H	1.060276	-4.039193	-3.766896
H	2.584256	-4.527438	-2.936799
H	1.022212	-5.304175	-2.475252
C	2.816827	-6.948891	-0.613553
H	1.943457	-7.613210	-0.496343
H	3.092824	-6.893630	-1.686435
H	3.671345	-7.352136	-0.031748
C	8.012116	0.100873	1.927409
H	8.499486	0.812191	2.613591
H	8.268887	-0.937538	2.212026
H	8.338827	0.304666	0.890560
C	-4.912724	-3.791258	4.335034
H	-4.430695	-2.916052	4.819255
H	-5.990824	-3.793093	4.573194
H	-4.452636	-4.725584	4.718861
C	-5.092089	-2.661655	-4.001976
H	-5.135534	-3.703277	-4.366186
H	-5.957582	-2.097779	-4.408462
H	-4.148993	-2.189629	-4.349915
C	2.128133	2.759818	2.544864
H	3.132691	2.996960	2.939075
H	1.477220	2.417441	3.376416
H	2.206725	1.946799	1.793830
C	-4.709698	4.103256	-4.133200
H	-5.783218	3.996554	-4.368435
H	-4.370999	5.119351	-4.424763
H	-4.132189	3.344314	-4.701637
C	-4.544040	2.806993	4.160883
H	-4.422436	3.842769	4.523938
H	-5.417896	2.345408	4.666195
H	-3.629638	2.222571	4.396712
C	-0.444761	0.332897	-0.153819
H	0.321411	-0.456481	-0.228619
Cl	-1.236721	0.436684	-1.784881
H	0.026941	1.311532	0.031894
C	-1.492950	0.008233	0.891582
H	-2.254603	0.798867	0.974836

```
 H      -1.971197   -0.966018    0.714126
 Cl     -0.672764   -0.099707    2.514608
 C       5.915848   -0.163486   -1.180150
 H       6.093248   -0.975307   -1.895133
 H       6.806663    0.436523   -0.960141
 H       7.085726   -1.672526    0.300157
```

Minimum energy conformer of 1,4 product with DCE inside the cavity
Energy = -4263.379155

```
 C    -2.527010    -3.741173     0.251287
 C     0.285544    -3.985207     0.823860
 C    -0.138545    -3.551286    -0.473570
 C    -1.551034    -3.506604    -0.768688
 C    -1.927015    -3.243755    -2.111265
 H    -2.971929    -3.284170    -2.383567
 C     0.779904    -3.200060    -1.489919
 H     1.837490    -3.155536    -1.277895
 C    -4.020294    -3.605744    -0.032273
 H    -4.571941    -3.963944     0.839677
 H    -4.288680    -4.267098    -0.859608
 O    -3.008145    -4.397495     2.475393
 C    -2.581553    -4.498133     3.840202
 H    -1.984225    -5.402481     4.009736
 H    -2.005283    -3.614236     4.140591
 H    -3.499143    -4.557363     4.428692
 O     1.348512    -2.510746    -3.657205
 C     0.946563    -2.089720    -4.964944
 H     0.255808    -1.238463    -4.914190
 H     1.864127    -1.784566    -5.471390
 H     0.476121    -2.908596    -5.524720
 C    -2.063014    -4.117795     1.508415
 C    -0.678559    -4.255244     1.773277
 C    -1.008220    -2.946948    -3.095558
 C     0.363931    -2.875780    -2.768530
 C    -4.471862    -2.191155    -0.378215
 C    -4.924699    -1.877581    -1.658195
 C    -5.232449    -0.543507    -2.013556
 H    -5.561805    -0.308797    -3.019768
 C    -5.118345     0.474652    -1.094868
 H    -5.356803     1.483700    -1.404079
 C    -4.745922     0.213540     0.247200
 C    -4.456866    -1.152857     0.606024
 C    -4.190146    -1.431909     1.969708
 H    -3.969538    -2.444563     2.280626
 C    -4.227834    -0.440192     2.921947
 H    -4.044094    -0.699319     3.958424
 C    -4.489233     0.901473     2.558742
 C    -4.691030     1.257061     1.225652
 C    -4.882448     2.716725     0.829465
 H    -5.832512     2.824034     0.301378
 H    -4.969245     3.313394     1.740599
 O    -4.555184     1.908702     3.498349
 C    -4.107510     1.632204     4.831855
 H    -3.077097     1.255185     4.833051
 H    -4.149370     2.588223     5.356657
 H    -4.765280     0.912278     5.334060
 O    -5.064759    -2.918933    -2.552073
 C    -5.294201    -2.612048    -3.932878
 H    -4.506797    -1.958567    -4.329630
 H    -5.270215    -3.571635    -4.452353
 H    -6.273958    -2.142077    -4.082446
 C    -3.777475     3.301622    -0.046800
 C    -4.056245     3.745158    -1.336526
 C    -3.029371     4.263509    -2.160904
 H    -3.256303     4.602224    -3.165783
 C    -1.731517     4.340313    -1.710156
 H    -0.974720     4.710377    -2.388542
 C    -1.373361     3.929319    -0.400566
 C    -2.441305     3.444821     0.446593
 C    -2.129654     3.136528     1.792952
 H    -2.913808     2.832553     2.470786
 C    -0.849119     3.251234     2.275363
 H    -0.649897     3.017696     3.314712
 C     0.201638     3.666184     1.429662
 C    -0.028255     4.002518     0.094946
 C     1.154158     4.451301    -0.765134
 H     0.786815     4.942136    -1.666706
 H     1.715741     5.206168    -0.211703
 O     1.480450     3.811478     1.900478
 C     1.868583     3.077612     3.068764
 H     1.576977     2.025161     2.983047
 H     2.956653     3.156124     3.109182
 H     1.435659     3.510766     3.979737
 O    -5.365132     3.665231    -1.765662
 C    -5.647929     3.895156    -3.150783
 H    -5.060708     3.225426    -3.792029
 H    -6.710916     3.678401    -3.271189
 H    -5.455812     4.937828    -3.432961
 C     2.077693     3.307887    -1.170066
 C     1.710947     2.492102    -2.241527
 C     2.466154     1.344503    -2.574200
 H     2.150322     0.695980    -3.383282
 C     3.599283     1.021192    -1.862547
 H     4.135555     0.119495    -2.126670
 C     4.071716     1.842814    -0.808130
 C     3.314209     3.035035    -0.500305
```

C	3.877752	3.932393	0.442349
H	3.376487	4.863223	0.669628
C	5.046784	3.645805	1.106655
H	5.439555	4.368230	1.813591
C	5.725712	2.426984	0.873769
C	5.257743	1.520337	-0.074884
C	6.065247	0.259882	-0.337003
H	6.628414	0.398746	-1.264623
H	6.815548	0.175751	0.454802
O	6.868827	2.081304	1.564505
C	7.310878	2.934785	2.624447
H	7.612776	3.920376	2.247591
H	6.534347	3.057452	3.390619
H	8.177183	2.434113	3.060791
O	0.595325	2.865183	-2.944800
C	0.269531	2.164504	-4.147055
H	0.040689	1.112335	-3.947438
H	-0.623303	2.658173	-4.535561
H	1.082909	2.236304	-4.881111
C	5.328364	-1.062429	-0.438196
C	5.584443	-1.898130	-1.524448
C	4.943050	-3.151895	-1.637321
H	5.173796	-3.815921	-2.462567
C	4.010542	-3.545912	-0.705892
H	3.542674	-4.514853	-0.836586
C	3.667745	-2.727144	0.402118
C	4.403311	-1.494124	0.561520
C	4.185036	-0.747582	1.747603
H	4.726754	0.176768	1.905280
C	3.303109	-1.176678	2.714229
H	3.183087	-0.590635	3.618604
C	2.543762	-2.354667	2.522445
C	2.661162	-3.098692	1.349211
C	1.746121	-4.293508	1.151760
H	1.751002	-4.886818	2.071615
H	2.137483	-4.943024	0.365485
O	1.665776	-2.831768	3.462946
C	1.661740	-2.232255	4.761255
H	1.345868	-1.183197	4.723029
H	0.936200	-2.803256	5.343583
H	2.650862	-2.300613	5.232325
O	6.474673	-1.443265	-2.472533
C	6.634520	-2.201631	-3.676758
H	5.675760	-2.333403	-4.194312
H	7.310817	-1.616467	-4.302438
H	7.082595	-3.183342	-3.476964
H	-0.353486	-4.591467	2.750466
H	-1.356858	-2.747918	-4.103171
C	-1.196880	-0.240232	1.074138
Cl	-0.371588	-0.226219	2.709826
H	-2.037343	0.444206	1.159984
H	-1.542805	-1.260191	0.922488
C	-0.224135	0.198081	0.007131
Cl	-1.168028	0.240385	-1.559628
H	0.591715	-0.509640	-0.139115
H	0.153787	1.207027	0.165680

Minimum energy conformer of 1,5 product with DCE inside the cavity
Energy = -4263.374956

O	-1.276614	-3.176772	-2.532367
O	4.825638	-2.083647	3.396127
O	4.909197	2.130721	-3.129499
O	-1.636596	3.109217	-2.327302
O	4.815563	-4.376813	-1.703386
O	4.722682	4.497786	1.906512
O	-1.225741	3.160034	2.544379
O	-7.117804	1.434522	-2.212350
O	-1.631641	-3.077275	2.421455
O	-7.084595	-1.426714	2.317065
C	4.800129	-0.683148	0.004376
C	4.756203	-1.629968	1.074898
C	-4.550362	1.736131	0.399706
C	4.757380	0.735743	0.264719
C	4.745128	1.153751	1.618675
H	4.742024	2.208620	1.857994
C	4.787507	-1.149019	2.382922
C	-2.568922	-3.236702	-0.561606
C	-4.196834	-1.143541	-1.567613
H	-4.781505	-0.321784	-1.960506
C	4.706286	-3.129026	0.801448
H	5.577767	-3.413192	0.206974
H	4.801551	-3.652764	1.755746
C	3.473983	4.362334	1.335813
C	-0.401475	3.250825	-1.750965
C	4.746421	1.678992	-0.811391
C	-3.766543	2.855183	-0.073449
C	2.124474	3.578569	-0.520973
C	3.553858	-4.241578	-1.163209
C	2.163640	-3.529851	0.692839
C	-3.096939	1.607376	2.351356
H	-2.857705	1.145294	3.302354
C	-2.328800	-2.667818	-1.812439
C	-4.539403	-1.716326	-0.316898
C	4.873108	1.198310	-2.114622
C	3.401205	3.699771	0.113505
C	-2.591894	3.272287	0.632130

C	-3.735669	-2.819306	0.158747
C	-5.657310	1.242867	-0.360925
C	-0.359396	3.869182	-0.500197
C	-2.300951	2.663969	1.853521
C	1.072026	4.710337	1.370241
H	0.203347	5.078550	1.898802
C	-1.665194	4.366715	0.116918
H	-2.185020	4.962033	-0.636042
H	-1.441947	5.048144	0.938719
C	0.918049	4.052111	0.122747
C	4.914371	-1.100470	-1.345112
H	4.985212	-2.153659	-1.580990
C	2.003515	2.996481	-1.805740
H	2.888718	2.686477	-2.342182
C	0.967947	-3.958114	-0.002939
C	-0.323674	-3.792938	0.599598
C	4.669308	3.178645	-0.552967
H	5.521088	3.469408	0.066655
H	4.796531	3.693056	-1.508754
C	4.777329	0.240437	2.647939
H	4.793243	0.601268	3.670499
C	-6.044520	1.936017	-1.505106
C	-3.138825	-1.619632	-2.307641
H	-2.926440	-1.176498	-3.274301
C	-5.343028	3.091492	-1.922546
H	-5.666367	3.640995	-2.799513
C	-5.643439	-1.229577	0.451578
C	1.148122	-4.550273	-1.279738
H	0.287522	-4.869057	-1.851612
C	4.959599	-0.188854	-2.373738
H	5.057781	-0.549034	-3.391412
C	2.306666	4.865945	1.957201
H	2.374172	5.378612	2.910405
C	2.396763	-4.695864	-1.839094
H	2.482537	-5.156243	-2.817097
C	-1.614674	-4.305560	-0.043267
H	-2.127391	-4.923002	0.697493
H	-1.362699	-4.970140	-0.870518
C	0.777872	2.840399	-2.408686
H	0.724997	2.389048	-3.392228
C	2.018552	-3.015111	2.003163
H	2.893023	-2.748681	2.578308
C	3.456172	-3.633104	0.085552
C	-6.013656	-1.921311	1.602334
C	-4.178353	1.148263	1.634652
H	-4.753576	0.322075	2.031311
C	-6.434813	0.002070	0.045670
H	-7.096295	-0.262748	-0.784856
H	-7.098455	0.260136	0.876572
C	-4.235620	3.525666	-1.232804
H	-3.707185	4.390893	-1.609533
C	0.780566	-2.857864	2.574998
H	0.706990	-2.452872	3.577926
C	-5.298007	-3.068910	2.017417
H	-5.611377	-3.621566	2.896026
C	-0.387244	-3.217577	1.869804
C	-4.188753	-3.491003	1.323142
H	-3.649034	-4.350492	1.696224
C	-1.201192	-2.883080	-3.930395
H	-1.001534	-1.821220	-4.109585
H	-0.359932	-3.469108	-4.305140
H	-2.123291	-3.182202	-4.445278
C	4.935860	-4.798968	-3.066867
H	4.379503	-4.133179	-3.738849
H	6.001534	-4.743473	-3.295993
H	4.588688	-5.831163	-3.199960
C	4.653204	-1.639820	4.747222
H	3.709642	-1.092615	4.867874
H	4.629284	-2.548597	5.351081
H	5.489717	-1.008604	5.071732
C	4.817155	5.017525	3.237284
H	4.232076	4.414044	3.943099
H	5.875544	4.960319	3.497797
H	4.486927	6.062782	3.285095
C	-7.468917	2.056700	-3.452486
H	-6.631214	2.038571	-4.161731
H	-8.296436	1.466949	-3.851168
H	-7.799948	3.092399	-3.302923
C	-0.874419	2.573642	3.798484
H	-0.653168	1.505598	3.691156
H	0.027028	3.098486	4.120243
H	-1.669028	2.713919	4.543192
C	-1.778097	2.257174	-3.467558
H	-1.359838	1.262143	-3.273911
H	-2.854313	2.176233	-3.632079
H	-1.301904	2.689444	-4.357308
C	4.812590	1.672848	-4.484088
H	3.908952	1.069044	-4.634458
H	4.755592	2.576855	-5.092882
H	5.697111	1.092918	-4.775251
C	-1.790545	-2.245255	3.574060
H	-1.346316	-1.257017	3.407662
H	-2.869410	-2.145714	3.708368
H	-1.350492	-2.704385	4.468907
C	-7.419420	-2.047079	3.562715
H	-6.575145	-2.020418	4.263797

H	-8.247251	-1.461754	3.967189
H	-7.744526	-3.085542	3.419765
C	1.583006	-0.377151	-1.039050
H	2.242800	0.486369	-1.014472
Cl	1.278331	-0.718608	-2.815294
H	2.080359	-1.258122	-0.641867
C	0.267705	-0.099085	-0.353116
H	-0.372451	-0.978673	-0.291344
H	-0.259860	0.742563	-0.797288
Cl	0.651447	0.399935	1.366240

Minimum energy conformer of 1,4 product without DCE
Energy = -3264.280189

C	0.531766	2.195517	0.638817
C	1.256987	3.345197	0.921311
C	-1.262578	3.436682	-0.465766
C	0.686529	4.603502	0.529449
C	-0.701934	2.249942	-0.056898
C	-0.566814	4.657219	-0.185109
C	1.319728	5.841958	0.814692
H	-1.236100	1.323980	-0.238930
H	-2.019208	5.969742	-1.131941
C	0.791499	7.057391	0.422432
H	2.247653	5.856007	1.375460
H	1.320381	7.969653	0.675277
C	-0.425682	7.095909	-0.294396
C	-1.085794	5.911590	-0.582391
C	-2.609852	3.475924	-1.170958
H	-2.467315	3.854064	-2.187886
H	-3.245886	4.213675	-0.664119
C	2.604525	3.324664	1.627854
H	2.463314	3.531301	2.693998
H	3.201537	4.158433	1.240686
C	-3.445137	1.461344	-2.430696
C	-3.344585	2.160372	-1.231355
C	-4.715287	-0.305681	-1.357663
C	-3.927088	1.602703	-0.052540
C	-4.171865	0.247518	-2.495536
C	-4.580010	0.319497	-0.090867
C	-3.858633	2.272906	1.195648
H	-4.296155	-0.266516	-3.441980
H	-5.259986	-1.236587	-1.442790
C	-4.382900	1.711271	2.337881
H	-3.371315	3.238864	1.266005
C	-4.971850	0.425153	2.301668
C	-5.050554	-0.294202	1.110098
C	3.827200	1.366024	2.618712
C	3.444775	2.077098	1.495466
C	4.979751	-0.293829	1.281284
C	3.890376	1.625720	0.212088
C	4.607222	0.192422	2.512506
C	4.612340	0.386786	0.086170
C	3.606256	2.354659	-0.967820
H	4.911459	-0.317623	3.423642
H	5.586160	-1.187910	1.226939
C	4.004753	1.895508	-2.204382
H	3.061584	3.290237	-0.906921
H	3.784823	2.488500	-3.084894
C	4.667784	0.652266	-2.325900
C	4.944507	-0.128494	-1.203635
C	-5.583421	-1.718282	1.115461
H	-6.531942	-1.752754	0.572786
H	-5.814521	-1.993104	2.148051
C	5.532534	-1.523590	-1.361513
H	6.512269	-1.566584	-0.879274
H	5.704062	-1.712475	-2.424032
C	5.155377	-3.456256	0.225677
C	4.658343	-2.623689	-0.774187
C	2.987125	-4.496425	0.525674
C	3.302866	-2.792765	-1.195310
C	4.316553	-4.395893	0.868185
C	2.421875	-3.703929	-0.504112
C	2.784853	-2.075361	-2.307378
H	4.708085	-5.031514	1.654285
H	2.369233	-5.193900	1.076811
C	1.467122	-2.196773	-2.674829
H	3.440350	-1.447755	-2.895444
H	1.091316	-1.667872	-3.546903
C	0.589571	-3.026570	-1.941728
C	1.032911	-3.777183	-0.861587
C	-4.651748	-2.759107	0.511812
C	-2.914959	-4.590117	-0.764744
C	-3.299889	-2.899461	0.957296
C	-4.235458	-4.505998	-1.140500
C	-2.384487	-3.786768	0.277389
C	-2.807962	-2.169050	2.068686
H	-4.596955	-5.143156	-1.939635
H	-2.273328	-5.275078	-1.304222
C	-1.490877	-2.256261	2.460844
H	-3.478365	-1.540888	2.638699
H	-1.155892	-1.693828	3.325521
C	-0.584556	-3.070485	1.744984
C	-1.004116	-3.825711	0.650112
H	-4.316100	2.256794	3.272344
O	-1.021600	8.252135	-0.733664
C	-0.371291	9.497324	-0.445884

H	-0.284382	9.661547	0.635750
H	-1.009423	10.268524	-0.880415
H	0.623897	9.543954	-0.906182
C	-5.105699	-3.582460	-0.514972
O	3.468040	1.812234	3.888704
C	2.356690	1.094763	4.458139
H	2.154563	1.555168	5.428287
H	2.605259	0.034883	4.602646
H	1.473780	1.174476	3.812269
O	1.002487	0.985817	1.093196
C	0.874056	-0.139930	0.203513
H	-0.118004	-0.596928	0.281272
H	1.626891	-0.863783	0.514172
H	1.070740	0.157744	-0.830458
O	-2.818912	2.008778	-3.525970
C	-2.925974	1.332181	-4.783794
H	-2.352770	1.936052	-5.489282
H	-3.969281	1.270710	-5.118536
H	-2.496137	0.324009	-4.732869
O	5.033468	0.134285	-3.548668
C	4.624200	0.822214	-4.737290
H	4.968911	0.201098	-5.565732
H	5.089231	1.813374	-4.806592
H	3.532415	0.923050	-4.782817
O	-5.442159	-0.197308	3.437417
C	-5.233439	0.437010	4.704799
H	-4.165957	0.606811	4.894454
H	-5.630065	-0.258076	5.446801
H	-5.775488	1.388662	4.770909
O	0.737803	-3.179840	2.087061
C	1.268632	-2.317904	3.097677
H	0.857781	-2.558234	4.087014
H	1.076108	-1.265288	2.865171
H	2.345292	-2.497409	3.093922
O	-6.415470	-3.422241	-0.913954
C	-6.884629	-4.166675	-2.043956
H	-6.300569	-3.937673	-2.944598
H	-7.919159	-3.851550	-2.190880
H	-6.856460	-5.246644	-1.852133
O	6.475766	-3.288397	0.580446
O	-0.721418	-3.167365	-2.363139
C	6.982483	-4.015257	1.706467
H	6.416742	-3.783226	2.617802
H	8.015944	-3.685198	1.824455
H	6.963348	-5.097179	1.525977
C	-1.512348	-1.966016	-2.340655
H	-1.607006	-1.588553	-1.316618
H	-2.499855	-2.244944	-2.707993
H	-1.086035	-1.185133	-2.982117
C	0.032166	-4.642640	-0.106415
H	-0.460175	-5.306934	-0.816820
H	0.555005	-5.286630	0.602025

Minimum energy conformer of 1,5 product without DCE
Energy = -3264. 272065

C	2.145279	2.152569	-1.966570
C	2.581572	3.020522	-0.975929
C	4.119459	0.766220	-1.763100
C	3.849235	2.760019	-0.354746
C	2.935406	1.061936	-2.393661
C	4.591926	1.570239	-0.691967
C	4.405896	3.611799	0.631572
H	2.589748	0.460929	-3.229636
C	5.562213	3.284638	1.302262
H	3.904819	4.533052	0.899696
C	6.215985	2.055803	1.048687
C	5.733183	1.183711	0.074056
C	6.415613	-0.150415	-0.135208
H	7.195682	-0.257488	0.624486
C	1.698842	4.197263	-0.581755
H	1.446662	4.756195	-1.483208
H	2.262640	4.883451	0.052641
C	6.095237	-2.486309	-0.923053
C	5.585708	-1.432493	-0.158831
C	4.171287	-3.871090	-0.439359
C	4.346828	-1.628969	0.530531
C	5.394442	-3.705445	-1.047642
C	3.583844	-2.845643	0.339091
C	3.799980	-0.643624	1.399924
H	5.804370	-4.516896	-1.638153
H	3.644088	-4.802501	-0.601503
C	2.541279	-0.782636	1.928007
H	4.375664	0.238093	1.642292
C	1.758176	-1.922536	1.643522
C	2.261101	-2.970786	0.888021
C	0.535233	3.318228	1.460240
C	0.427521	3.806658	0.157518
C	-1.865650	3.073448	1.649180
C	-0.876593	3.964355	-0.409340
C	-0.610654	2.957004	2.202654
C	-2.051177	3.567925	0.334285
C	-1.085089	4.528251	-1.694706
H	-0.513006	2.595213	3.220407
H	-2.723404	2.810176	2.251915
C	-2.347930	4.697129	-2.212752

H	-0.238732	4.813510	-2.305617
H	-2.457188	5.141573	-3.195663
C	-3.489549	4.291616	-1.481864
C	-3.359910	3.697524	-0.229107
C	1.387123	-4.198899	0.668103
H	1.129374	-4.612300	1.643907
H	1.950088	-4.974782	0.148863
C	-4.596727	3.242786	0.533610
H	-5.477646	3.517832	-0.051711
H	-4.660761	3.805517	1.468174
C	-4.754536	1.346786	2.197476
C	-4.677093	1.759252	0.868046
C	-4.859653	-0.986193	1.543374
C	-4.730992	0.765276	-0.158909
C	-4.846120	-0.024898	2.528514
C	-4.794883	-0.638552	0.170403
C	-4.765537	1.117587	-1.531926
H	-4.917322	-0.334541	3.565455
C	-4.846157	0.158696	-2.516403
H	-4.761117	2.159019	-1.822745
H	-4.890123	0.473584	-3.553253
C	-4.880431	-1.216070	-2.185463
C	-4.832285	-1.633601	-0.856637
C	0.124837	-3.897299	-0.122596
C	-2.136040	-3.147243	-1.660111
C	-1.191264	-4.016752	0.434921
C	-0.875004	-3.103057	-2.204994
C	-2.343016	-3.594823	-0.326537
C	-1.421001	-4.526407	1.736116
H	-0.736914	-2.801292	-3.240010
H	-2.983005	-2.875006	-2.274604
C	-2.691381	-4.636123	2.253893
H	-0.588146	-4.839625	2.353642
H	-2.821141	-5.049940	3.247448
C	-3.811624	-4.204031	1.507356
C	-3.654285	-3.645675	0.239735
H	2.136975	-0.021070	2.587496
O	7.320540	1.650367	1.762392
C	7.786009	2.478457	2.833733
H	8.124020	3.455934	2.466968
H	8.631439	1.943250	3.269580
H	7.009599	2.619972	3.596377
C	0.250404	-3.476204	-1.438041
O	1.802792	3.228560	1.974274
C	1.974564	2.889970	3.350887
H	1.565682	1.898362	3.580395
H	1.505343	3.634616	4.006944
H	3.054055	2.880769	3.512097
O	0.932344	2.394113	-2.592116
C	-0.059524	1.371727	-2.368370
H	-0.988582	1.744681	-2.803493
H	-0.201799	1.207299	-1.293711
H	0.222575	0.427550	-2.849593
O	7.300702	-2.268670	-1.550256
C	7.801449	-3.270156	-2.443758
H	8.721580	-2.855670	-2.859348
H	7.091048	-3.473324	-3.255105
H	8.030004	-4.202790	-1.912684
O	-4.767742	4.446139	-1.976327
C	-4.933066	4.898488	-3.324952
H	-4.559398	5.921969	-3.454009
H	-6.008799	4.879845	-3.508245
H	-4.428134	4.230020	-4.034021
O	0.490332	-2.023083	2.194043
C	-0.455253	-1.066179	1.671186
H	-0.150626	-0.036266	1.892149
H	-1.410889	-1.280380	2.153586
H	-0.560316	-1.191129	0.587136
O	-5.095425	-4.289574	2.000188
C	-5.284488	-4.699754	3.359925
H	-4.957436	-5.735312	3.515074
H	-6.358626	-4.627549	3.538560
H	-4.751082	-4.036646	4.052790
O	1.506425	-3.488597	-2.019292
C	1.976134	-2.209466	-2.474052
H	1.947095	-1.481851	-1.657089
H	1.384100	-1.840314	-3.321452
H	3.011404	-2.356707	-2.787130
O	-4.741598	2.331599	3.162652
O	-4.960696	-2.197939	-3.150209
C	-4.653257	1.944407	4.538621
H	-5.548305	1.397157	4.859675
H	-4.578833	2.877829	5.099258
H	-3.761091	1.332567	4.723369
C	-4.832009	-1.820751	-4.526027
H	-5.671880	-1.193661	-4.850264
H	-4.841218	-2.757170	-5.086438
H	-3.886766	-1.293108	-4.706604
C	-4.863244	-3.118897	-0.520601
H	-4.971888	-3.678381	-1.452752
H	-5.757947	-3.329534	0.070381
H	-4.957810	-2.022516	1.834477
H	4.691417	-0.098307	-2.080294
H	6.958339	-0.113050	-1.086899
H	5.948683	3.969946	2.048303

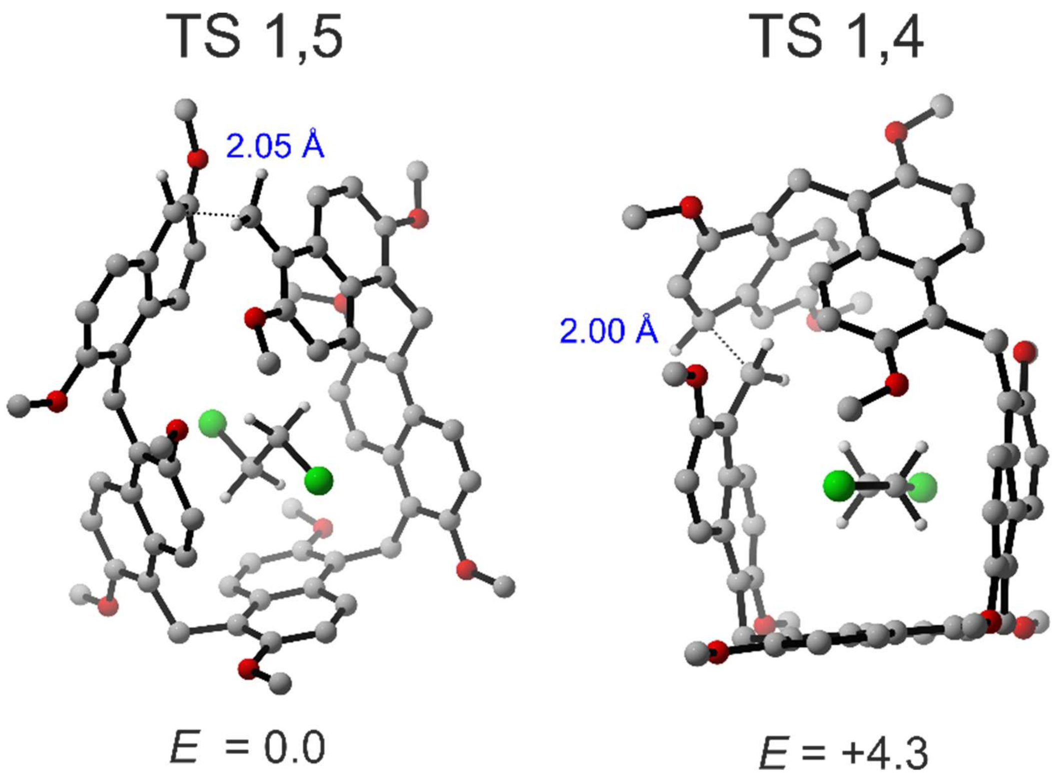

Figure S78: The most stable transition states for the macrocyclization step leading to 1,5 and 1,4 products. Noncritical hydrogen atoms have been omitted for clarity. Relative energy is expressed in kcal/mol, The forming bond distances (Å) are highlighted in blue.

.

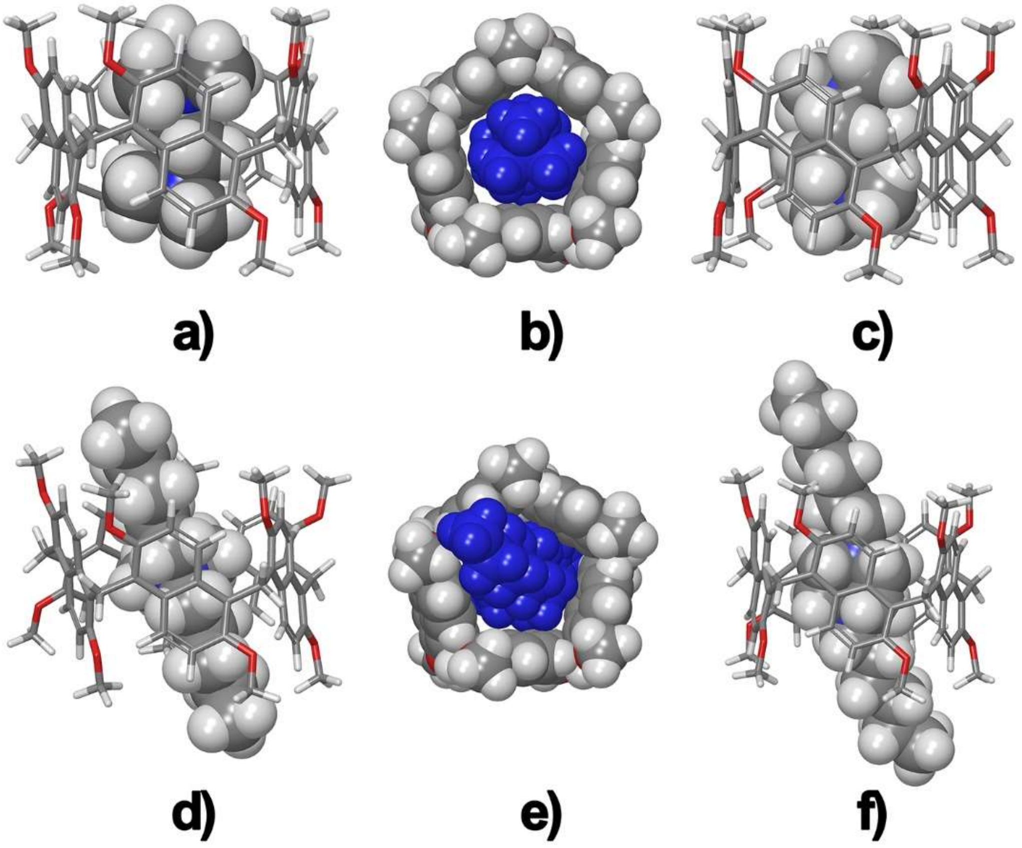

Figure S79. DFT-optimized structures (B97D3/SVP/SVPFIT) of the: a and b) different views of the $\mathbf{10}^{2+} \subset \mathbf{1}$ endo-complex, stabilized by C–H··· π interactions (C–H··· $\pi^{centroids}$ distance of 2.80 Å. c) $\mathbf{9}^{2+} \subset \mathbf{1}$ endo-complex. d) $\mathbf{8}^{+} \subset \mathbf{1}$ pseudorotaxane. The N^{+}-methyl groups of $\mathbf{8}^{+}$ are involved in C–H··· π interactions (C–H··· $\pi^{centroids}$ distance of 2.45 Å and C–H··· $\pi^{centroids}$ angle of 160°). e, f) Different views of the $\mathbf{4}^{2+} \subset \mathbf{1}$ pseudorotaxane. C–H··· π interactions were detected between the ethylene groups $^{+}NCH_2CH_2N^{+}$ and the aromatic walls of **1** (C–H··· $\pi^{centroids}$ distance of 2.72 Å).

References

[1] Williams, D. B. G.; Lawton, M. Drying of Organic Solvents: Quantitative Evaluation of the Efficiency of Several Desiccants. *J. Org. Chem.* **2010**, *75*, 8351-8354.

[2] Fulmer, G. R.; Miller, A. J. M.; Sherden, N. H.; Gottlieb, H. E.; Nudelman, A.; Stoltz, B.M.; Bercaw, J.E.; Goldberg, K.I. NMR Chemical Shifts of Trace Impurities: Common Laboratory Solvents, Organics, and Gases in Deuterated Solvents Relevant to the Organometallic Chemist. *Organometallics* **2010**, *29*, 2176–2179.

[3] Gaeta, C.; Troisi, F.; Neri, P. endo-Cavity Complexation and Through-the-Annulus Threading of Large Calixarenes Induced by Very Loose Alkylammonium Ion Pairs. *Org. Lett.* **2010**, *12*, 2092-2095.

[4] Bakić, M. T.; Iuliano, V.; Talotta, C.; Geremia, S.; Hickey, N.; Spinella, A.; De Rosa, M.; Soriente, A.; Gaeta, C.; Neri, P. Threading of Conformationally Stable Calix[6]arene Wheels Substituted at the Methylene Bridges. *J. Org. Chem.* **2019**, *84*, 11922-11927.

[5] Kabsch, W. XDS. *Acta Crystallogr.* **2010**, *D66*, 125-132.

[6] Kabsch, W. Integration, Scaling, Space-Group Assignment and Post-Refinement. *Acta Crystallogr.* **2010**, *D66*, 133-144.

[7] Sheldrick, G. M. SHELXT - Integrated Space-Group and Crystal-Structure Determination. *Acta Crystallogr.* **2015**, *A71*, 3-8.

[8] Sheldrick, G. M. A Short History of SHELX. *Acta Crystallogr.* **2008**, *A64*, 112-122.

[9] Farrugia, L.J. WinGX and ORTEP for Windows: an Update. *J. Appl. Cryst.* **2012**, *45*, 849−854.
